# QTL Mapping in Three Connected Populations Reveals a Set of Consensus Genomic Regions for Low Temperature Germination Ability in *Zea mays* L.

**DOI:** 10.3389/fpls.2018.00065

**Published:** 2018-01-31

**Authors:** Xuhui Li, Guihua Wang, Junjie Fu, Li Li, Guangyao Jia, Lisha Ren, Thomas Lubberstedt, Guoying Wang, Jianhua Wang, Riliang Gu

**Affiliations:** ^1^Center of Seed Science and Technology, Beijing Key Laboratory of Crop Genetics and Breeding, Innovation Center for Seed Technology (Ministry of Agriculture), China Agricultural University, Beijing, China; ^2^Institute of Crop Sciences, Chinese Academy of Agricultural Sciences, Beijing, China; ^3^Department of Agronomy, Iowa State University, Ames, IA, United States

**Keywords:** low temperature, seed vigor, germination, QTL mapping, maize

## Abstract

Improving seed vigor in response to cold stress is an important breeding objective in maize that allows early sowing. Using two cold tolerant inbred lines 220 and P9-10 and two susceptible lines Y1518 and PH4CV, three connected F_2:3_ populations were generated for detecting quantitative trait locus (QTL) related to seed low-temperature germination ability. At 10°C, two germination traits (emergence rate and germination index) were collected from a sand bed and three seedling traits (seedling root length, shoot length, and total length) were extracted from paper rolls. Significant correlations were found among all traits in all populations. Via single-population analysis, 43 QTL were detected with explained phenotypic variance of 0.62%∼39.44%. Seventeen QTL explained more than 10% phenotypic variance; of them sixteen (94.12%) inherited favorable alleles from the tolerant lines. After constructing a consensus map, three meta-QTL (mQTL) were identified to include at least two initial QTL from different populations. *mQTL1-1* included seven initial QTL for both germination and seedling traits; with three explaining more than 30% phenotypic variance. *mQTL2-1* and *mQTL9-1* covered two to three initial QTL. The favorable alleles of the QTL within these three mQTL regions were all inherited from the tolerant line 220 and P9-10. These results provided a basis for cloning of genes underlying the mQTL regions to uncover the molecular mechanisms of maize cold tolerance during germination.

## Introduction

Vigorous and uniform seedlings are necessary for achieving a high yield in crop production. However, various abiotic stresses occurring after sowing impair seedling establishment (Basnet et al., 2015; Liu et al., 2017). Maize (*Zea mays* L.) is one of the most important crops, accounting for 40% of the world’s cereal food production (Bouis and Welch, 2010). As maize originated from tropical and subtropical regions, it is sensitive to low temperature, particularly during early growth stages, where a relatively high temperature threshold for germination is required (Greaves, 1996; Verheul et al., 1996; Rodriguez et al., 2008; He et al., 2017; Pandit et al., 2017). Although global warming increases Earth surface temperature by 0.6–0.9°C in the past decades, low temperature stress still occurs in anytime of early spring at high-latitude regions, which results in failure of maize seedling establishment and yield loss (Stirling et al., 1991; Leipner et al., 1999; Hansen et al., 2000; Hu et al., 2016). Thus, improving a maize cultivar’s low-temperature germination ability (LTGA) is vital for maize yield production and global food security. Moreover, low temperature is one of the main ecological factor limiting maize distribution. High LTGA seed could be not only sown at high latitudes to extend the maize planting area, but also sown in early spring to extend crop growing season with benefit to crop rotation and annual yield output (Leipner et al., 2008; Frascaroli and Landi, 2017).

Plant low-temperature acclimation is a complex inherited quantitative trait controlled by several minor genes, and easily influenced by environment. Quantitative trait locus (QTL) mapping is a powerful approach to study and manipulate complex traits important in agriculture. QTL for low-temperature acclimation has been conducted in rice, wheat and bean (Fujino et al., 2004; Baga et al., 2007; Lu et al., 2014; Sallam et al., 2016; Zhang W.B. et al., 2017). In rice, dozens of QTL of cold adaption were identified, and two major QTL were successfully used for enhancing low-temperature acclimation in breeding programs (Fujino et al., 2008; Zhang Z. et al., 2017). In maize, traits at germination and seedling stages were conducted for mapping of QTL associated with low temperature using different populations under different temperatures. Using an F_2:3_ population, Rodriguez et al. (2014) identified three important genomic regions controlling seedling development under 15°C. Hund et al. (2004) found a large number of independently inherited loci for controlling seedling development at 15/13°C (day/night). Using recombinant inbred line (RIL) populations, Shi et al. (2016) identified 5 meta-QTL (mQTL) from 26 initial seed vigor related QTL at 18°C. Hu et al. (2016) detected 6 QTL for LTGA at 18/12°C (day/night). In addition, QTL related to leaf traits of seedling, such as photosynthesis related parameters, leaf area and weight, and nitrogen content had been investigated to reflect seed vigor under cold conditions (Fracheboud et al., 2002; Jompuk et al., 2005; Guerra-Peraza et al., 2011; Allam et al., 2016). Of these identified QTL, few were common in the same genomic regions across experiments, suggesting that QTL for maize cold tolerance are mainly determined by the specific genetic backgrounds and environmental conditions. Therefore, analysis of QTL using different genetic resources is necessary to enrich number of QTL and extract promising QTL for further fine-mapping or molecular breeding of LTGA in maize.

In this study, we employed three F_2:3_ populations, derived from four inbred lines, two tolerant and two susceptible to cold stress, to detect the QTL related to maize LTGA under sand bed and paper roll germination conditions. The objectives of this study were: (1) to analyze low temperature seed emergence and seedling performance of the three F_2:3_ populations and the four parental lines; (2) to identify QTL for LGTA from each population; (3) to integrate QTL from different populations and pinpoint mQTL for further fine-mapping or molecular breeding.

## Materials and Methods

### Plant Materials

**FIGURE 1:**
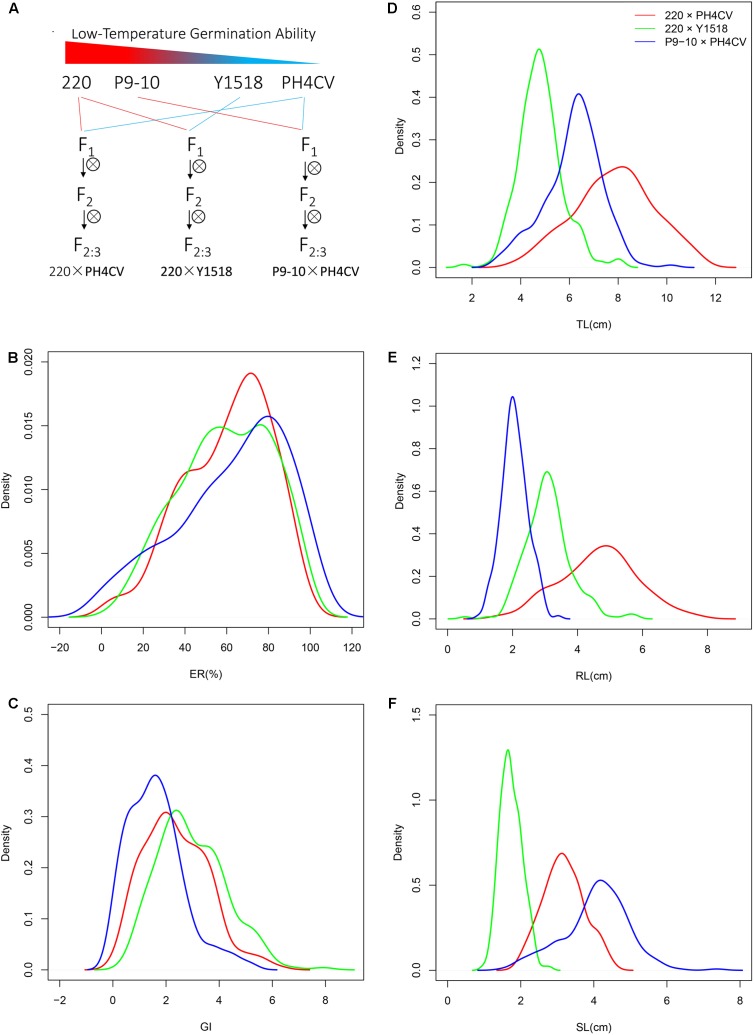
The density of frequency distribution of cold germination related traits in populations. **(A)** Strategy for constructing the three connected populations 220 × PH4CV, 220 × Y1518, and P9-10 × PH4CV. Traits emergence rate (ER, **B**) and germination index (GI, **C**) were collected from sand bed experiment, and total length (TL, **D**), root length (RL, **E**) and shoot length (SL, **F**) were collected from paper rolls experiment. Line in red, green, and blue represented trait performance in population 220 × PH4CV, 220 × Y1518, and P9-10 × PH4CV, respectively.

Based on a germplasm screening program targeting at seed vigor, inbred lines 220 and P9-10 were selected as cold tolerant lines, whereas PH4CV and Y1518 were susceptible. PH4CV was the paternal parent of XY335, a widely cultivated hybrid generated by the Pioneer Technology Co., Tieling, Jilin Province, China (Gu et al., 2017). The P9-10 was derived from the hybrid PN78599 (also called P78599) by using bicyclic breeding strategy (Wang et al., 2017). Another elite inbred line Y1518, a perfect material for maize transformation, was also applied in this study (Zhou et al., 1999). Besides that, an inbred line 220, which the pedigree was untraceable was collected from northeast China. Among that, 220 and P9-10 are flint-type maize, while PH4CV and Y1518 belong to dent-type. Three F_2:3_ populations, 220 × PH4CV, 220 × Y1518 and P9-10 × PH4CV, were generated by crossing a tolerant to a susceptible line (**Figure 1A**). F_2_ plant was grown in Shunyi, Beijing, China (116°.65’E longitude, 40’.13’N latitude) in 2014. After self-pollination, the F_2:3_ seed was harvested and used for vigor tests. By standard germination test (at 25°C) according to the International Seed Testing Association [ISTA] protocol (International Seed Testing Association [ISTA], 2015), 650 F_2:3_ family lines had initial germination percentages higher than 98%, representing 223, 212, and 215 lines for 220 × PH4CV, 220 × Y1518 and P9-10 × PH4CV, respectively. These lines were used for phenotypic evaluation and QTL mapping.

### Phenotype Evaluation

**FIGURE 2:**
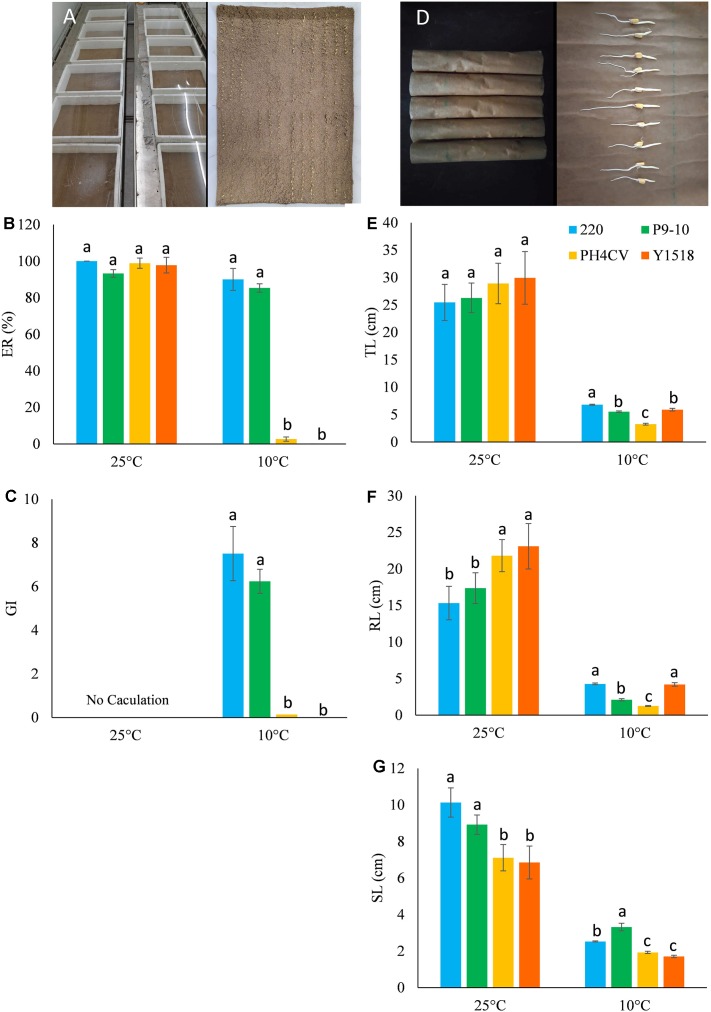
Phenotypic performance of the four parental lines (220, P9-10, PH4CV, and Y1518) germinated under low (10°C) and normal (25°C) temperature in two germination systems of sand bed and paper rolls. **(A–C)** Plant germinated in sand bed. **(A)** Picture of sand bed; **(B)** ER; **(C)** GI. **(D–G)** Plant germinated in paper rolls. **(D)** Picture of paper rolls; **(E)** TL; **(F)** RL; **(G)** SL. Different small letters within a temperature treatment indicated significant differences among genotypes.

Germination experiments were performed in both sand bed and paper roll experiments in a dark chamber at 10°C. In sand bed, after sieving and washing to remove soil, nutrient and other contaminations, sand was dried at 130°C for 5 h, moistened using distilled water to 16% content, and then sprayed in a plastic box to form a 2 cm thick bed (**Figure 2A**). After sterilizing with 1% sodium hypochlorite (NaClO) for 5 min and washing with distilled water, 30 seeds from each line were sown in the bed with the embryo side up, and then covered with additional 1 cm sand.

Once a shoot broke through the sand and became visible, it was counted as emerged plant. Emergence of plants was counted from 17 to 25 days after sowing (DAS) at 2-day intervals (in total five records). Emergence rate (ER) was expressed as percentage of emerged plants at 25th DAS to the total seed used. The germination index (GI) was calculated as

$$GI=\Sigma \frac{Gt}{Dt},$$

where *Gt* is the number of emerged plant at a given day (*Dt*, the days after sowing).

In paper rolls, 10 sterilized seeds were sown in a moist brown germination paper (Anchor Ltd., St. Paul, MN, United States) and another sheet of humid paper was used as a cover. Then the germination paper was rolled and put erectly in a sealed plastic bag (**Figure 2D**). After incubation at 10°C for 25 days, total length (TL), root length (RL), and shoot length (SL) of germinated seedlings was measured by a ruler. Both sand bed and paper roll tests were conducted in three independent experiments for each line.

### Data Analysis, QTL Detection, and Meta-QTL Analysis

The data were analyzed using the IBM Corp (2011) SPSS20.0 (IBM corp., Armonk, NY, United States) and the R statistical package (R Core Team, 2016). Mean of replicates was used for analysis of variance (ANOVA) and QTL mapping. Broad-sense heritability was calculated from an ANOVA fitting effect of genotype (G) and environment (E), as *H*^2^(%) = σ^2^_G_/(σ^2^_G_+σ^2^_E_/*r*) × 100%, where *H*^2^ is broad sense heritability, σ^2^_G_ is genotypic variance, σ^2^_E_ is error variance, *r* is the number of replications (Nyquist, 1991). The coefficients of variation (CV, %) were calculated as follows: CV = *s*/*x*, where *s* is the standard deviation and *x* is the mean of each trait within a population. The phenotypic correlation coefficients (Pearson’s) were determined by linear regressions at significant level *p* = 0.05.

Shoots from 30 plants for each line were sampled for genomic DNA extraction following the cetyltrimethylammonium bromide (CTAB) method (Murray and Thompson, 1980). Genotyping was undertaken by using a 6K SNP chip (Illumina Inc., San Diego, CA, United States). Polymorphic makers between each parental pair were used for genetic linkage map construction and QTL mapping using the software QTL IciMapping 4.1. Inclusive composite interval mapping (ICIM) algorithm was conducted for QTL mapping by scanning the genome every 1 cM (Li et al., 2007). The threshold likelihood of odds (LOD) value was determined with 1,000 permutations at a *P* = 0.05 level (Churchill and Doerge, 1994). The resulting LOD values were 3.70–6.83 for all trials (Supplementary Table S1).

Based on the SNP markers shared by different populations, an integrated map was built according to the user manual of QTL IciMapping software (Chardon et al., 2004; Meng et al., 2015). In brief, SNP makers overlapped on at least two genetic maps were selected as anchor markers and used to integrate corresponding linkage groups on individual linkage maps. The marker order and marker positions were calculated after calculating the order and the relative position (within each genetic map) of the anchored markers, followed by integrating of all the detected markers into one map. Then all QTL identified from the three populations were projected onto the integrated map based on their confidence interval. If QTL confidence intervals overlapped, they were considered as mQTL (Blanc et al., 2006; Cui et al., 2014; Han et al., 2014).

### Candidate-Gene Selection, RNA Extraction, and Quantification

Genes with well annotation involving in low-temperature adaption were collected from *Oryza sativa, Arabidopsis thaliana*, and *Sorghum bicolor*, and performed BLAST analysis in MaizeGDB^1^. Six genes located in our confident QTL interval were selected for quantitative PCR (qPCR) validation.

Two groups of seedlings growing in paper rolls were cultivated under 10°C and 25°C germination conditions, respectively, shoots were collected for RNA isolation once they have similar seedling length at 15 DAS (grown under 10°C) and 3 DAS (grown under 25°C), respectively. Ten shoots in each replication were pooled and grinded in liquid nitrogen for total RNA extraction using RNAprep pure Plant Kit [Tiangen Biotech (Beijing)]. RNA samples were treated with RNase-free DNase Kit (Invitrogen) to remove DNA contamination. Followed by reverse transcription reaction of cDNA with RT MasterMix (Applied Biological Materials Inc.), qPCR analysis was performed on Applied Biosystems QuantStudio 6 (Thermo Fisher) using qPCR MasterMix solution (Applied Biological Materials Inc.). The primers used in qPCR were listed in Supplementary Table S2. The maize *ZmGAPDH* gene was used as an internal control (Gu et al., 2013). The mean value from three replications was used as final gene expression.

## Results

### Low-Temperature Germination Ability in the Parental Lines

Germination (ER and GI) and seedling (TL, RL, and SL) performance of the four parental lines was evaluated in sand bed and paper rolls, respectively (**Figure 2**). At optimal temperature, the four parents had similar TL (25.46–29.95 cm) and ER (>95.0%), while genotypes 220 and P9-10 had significantly higher SL, but lower RL than PH4CV and Y1518. At low temperature, 220 and P9-10 showed increased ER and GI, and 1.5–2 times elevated SL compared to Y1518 and PH4CV. Genotypes 220 and Y1518 had similar RL (4.19–4.28 cm), which was significantly higher than RL (1.26–2.10 cm) of P9-10 and PH4CV. Genotype 220 displayed the longest TL (6.80 cm), followed by P9-10 (5.53 cm), Y1518 (5.90 cm), and PH4CV (3.26 cm). Taken together, 220 and P9-10 are cold tolerant, and Y1518 and PH4CV are cold susceptible lines.

### Low-Temperature Germination Ability in F_2:3_ Populations

The average values of ER among the three populations were similar, while those of the other four traits showed differences (**Figure 1**). Average GI in P9-10 × PH4CV was lower than that in 220 × PH4CV and 220 × Y1518. Average TL in 220 × PH4CV was higher than that in the other two populations, which was consistent with the respective parent means (**Figures 1D**, **2E**).

Coefficients of variation (CV) and broad-sense heritabilities (*H^2^*) were similar among these three populations for all traits (**Table 1**). CV for ER (35.5–42.15%) and GI (37.7–65.76%) were higher than for TL (19.03–21.05%), RL (19.46–25.71%) and SL (17.72–22.03%). *H^2^* estimates for ER and GI were lower (0.81–0.84) than for TL, RL, and SL (0.91–0.94). Higher CV and lower *H^2^* in ER and GI suggested that germination traits collected from sand bed might be more susceptible to environmental factors than seedling traits obtained from paper rolls.

**Table 1:** Mean and heritability (*H^2^*) estimates for traits related to low-temperature germination in population 220 × PH4CV, 220 × Y1518, and P9-10 × PH4CV.

Population	Trait^a^	Mean ± SD	CV (%)	Range	Kurtosis	Skewness	*H^2^*^b^
220 × PH4CV	ER (%)	60.48	34.50	4.00–98.00	-0.38	-0.50	0.84
	GI	2.38	51.08	0.07–6.29	0.00	0.50	0.83
	TL (cm)	7.84	21.05	3.73–11.37	-0.41	-0.13	0.93
	RL (cm)	4.66	25.71	1.58–7.78	-0.17	-0.05	0.94
	SL (cm)	3.18	17.69	1.87–4.55	-0.43	0.12	0.91
220 × Y1518	ER (%)	59.65	37.70	4.44–97.78	-0.86	-0.27	0.85
	GI	2.98	42.70	0.48–6.72	-0.42	0.39	0.82
	TL (cm)	4.82	19.15	1.66–8.08	1.64	0.55	0.93
	RL (cm)	3.09	23.17	0.53–5.78	1.95	0.53	0.92
	SL (cm)	1.73	17.72	0.96–2.80	0.13	0.40	0.94
P9-10 × PH4CV	ER (%)	62.67	42.15	0–100.00	-0.50	-0.62	0.82
	GI	1.61	65.76	0–5.23	0.88	0.87	0.81
	TL (cm)	6.16	19.03	2.96–10.16	0.45	-0.32	0.94
	RL (cm)	2.06	19.46	0.97–3.40	0.11	0.17	0.94
	SL (cm)	4.06	22.03	1.53–7.36	0.66	-0.23	0.93


*^*a*^Trait ER, GI, TL, RL, and SL represented emergence rate, germination index, total length, root length, and shoot length, respectively. ER and GI were evaluated in sand bed experiment, and TL, RL, and SL were evaluated in paper rolls experiment. ^*b*^*H^2^* represented broad-sense heritability.*

Within a population, the phenotypic distribution of all five traits were approximately consistent with normal distributions based on low values of skewness and kurtosis (below 1, except for kurtosis for TL and RL in 220 × Y1518; **Table 1** and **Figure 1**). Pairwise correlation coefficients among the five traits were significant and positive for all three populations, with closer correlations between traits within an assay (sand-bed versus paper-roll experiment, *r* = 0.78–0.97) than across those assays (*r* = 0.32–0.64) (**Table 2**).

**Table 2:** Phenotypic correlation between emergence rate (ER), germination index (GI), total length (TL), root length (RL), and shoot length (SL) in population 220 × PH4CV, 220 × Y1518 and P9-10 × PH4CV.

Populations	Traits	ER	GI	TL	RL	SL
220 × PH4CV	ER	1				
	GI	0.85 ^∗^	1			
	TL	0.55 ^∗^	0.49 ^∗^	1		
	RL	0.46 ^∗^	0.39 ^∗^	0.97 ^∗^	1	
	SL	0.64 ^∗^	0.59 ^∗^	0.86 ^∗^	0.72 ^∗^	1
220 × Y1518	ER	1				
	GI	0.93 ^∗^	1			
	TL	0.41 ^∗^	0.47 ^∗^	1		
	RL	0.32 ^∗^	0.36 ^∗^	0.96 ^∗^	1	
	SL	0.51 ^∗^	0.58 ^∗^	0.77 ^∗^	0.57 ^∗^	1
P9-10 × PH4CV	ER	1				
	GI	0.87 ^∗^	1			
	TL	0.52 ^∗^	0.48 ^∗^	1		
	RL	0.54 ^∗^	0.53 ^∗^	0.78 ^∗^	1	
	SL	0.42 ^∗^	0.39 ^∗^	0.95 ^∗^	0.56 ^∗^	1


*ER and GI were evaluated in sand bed experiment, and TL, RL, and SL were evaluated in paper rolls experiment. ^∗^Indicated significant difference at *p* < 0.05.*

### QTL Identification within Populations

A total of 5,179 SNP markers were scanned and resulted in 1,382, 1,500, and 1,419 markers that fit the expected 1:2:1 distribution ratio in F_2:3_ lines, and were polymorphic between the two parents of the population 220 × PH4CV, 220 × Y1518 and P9-10 × PH4CV, respectively (Supplementary Table S3). Based on these markers, three linkage maps were constructed with TLs of 1,689.8, 1,741.2, and 1,880.0 cM, and average interval sizes of 1.3, 1.3 and 1.5 cM for 220 × PH4CV, 220 × Y1518 and P9-10 × PH4CV, respectively (Supplementary Table S4).

**FIGURE 3:**
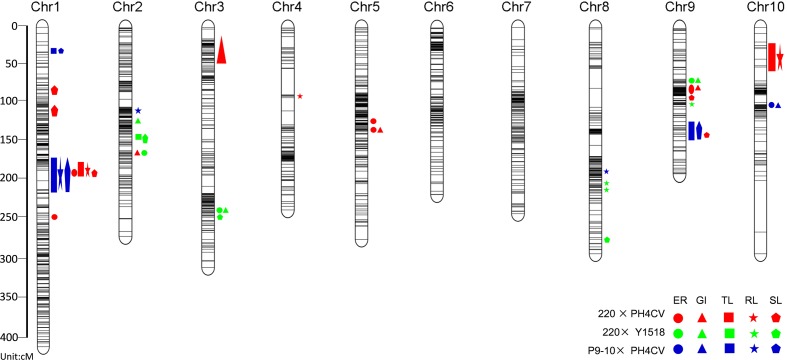
QTL for low temperature germination related traits represented in the consensus linkage map of the three populations of 220 × PH4CV, 220 × Y1518, and P9-10 × PH4CV. The circle, triangle, square, star and pentagon represented traits of ER, GI, TL, RL, and SL. ER and GI were collected from sand bed experiment, and TL, RL, and SL were collected from paper rolls experiment. Color red, green and blue indicated QTL detected from population 220 × PH4CV, 220 × Y1518, and P9-10 × PH4CV, respectively.

A total of 43 QTL were identified to be associated with LTGA with 19, 13, and 11 from 220 × PH4CV, 220 × Y1518 and P9-10 × PH4CV, respectively (**Figure 3** and Supplementary Table S5). The number of QTL identified for each trait ranged from 1 to 5 within a population. LOD values for individual QTL ranged from 3.82 to 36.28, and the explained phenotype variances varied from 0.62 to 39.44%. Three QTL (*qp1TL1-1*, *qp3TL1-2*, and *qp3SL1-2*) explained phenotypic variance of more than 30% and had LOD values exceeding 22. The other 13 QTL explained 10–30% of phenotype variance (Supplementary Table S5).

### QTL Analysis across Different Populations

Taking advantage of the same SNP chip used for genotyping, we integrated the three maps into a consensus linkage map (cMap) based on the markers shared by populations (**Figure 3** and Supplementary Table S5). The final cMap consisted of 2,693 SNP makers with a TL of 1,814.47 cM and an average marker interval length of 1.05 cM.

After projecting the 43 initial QTL on the cMap, 12 QTL (27.9%) overlapped, resulting in 3 mQTL (**Table 3**). *mQTL1-1* is located within the physical interval 175–184 Mb (base on B73 RefGen_v2) on chromosome 1, containing one and six QTL for germination and seedling traits, respectively, from population 220 × PH4CV and P9-10 × PH4CV. Like *mQTL1-1*, the *mQTL9-1* region (position 149–151 Mb on chromosome 9) harbored initial QTL from population 220 × PH4CV and P9-10 × PH4CV. This region only harbored three seedling specific QTL (one for TL and two for SL). The favorable alleles for QTL located in *mQTL1-1* and *mQTL9-1* were all inherited from the tolerant lines 220 or P9-10. The explained phenotypic variance for QTL in *mQTL1-1* was high (10.18–39.44%); while that for QTL in *mQTL9-1* was relatively low (3.02–5.04%).

**Table 3:** Meta-QTL (mQTL) detected from the consensus linkage map.

Name	Flanking Makers	CI (cM)	Initail QTL^b^	Favorable Allele	Reference^c^
*mQTL1-1*^a^	M1c174799835–M1c184012303	164.57–223.23	*qp1ER1-1*,	220 or P9-10	Fracheboud et al., 2002; Hund et al., 2004; Allam et al., 2016


			*qp1TL1-1*,		
			*qp1RL1-1*		
			*qp1SL1-3*,		
			*qp3TL1-2*,		
			*qp3RL1-1*,
			*qp3SL1-2*
*mQTL2-1*	M2c193833217–M2c199180638	163.75–165.86	*qp1GI2-1*,	220	Huang et al., 2013
			*qp2ER2-1*		
*mQTL9-1*	M9c149041431–M9c151277505	129.89–146.48	*qp1SL9-2*,	220 or P9-10	Jompuk et al., 2005; Allam et al., 2016; Hu et al., 2016


			*qp3TL9-1*,		
			*qp3SL9-1*		


*^*a*^A mQTL included at least two initial QTL from different populations of 220 × PH4CV, 220 × Y1518, and P9-10 × PH4CV in the consensus linkage map. ^*b*^Names of initial QTL were referred to Supplementary Table S3. P1, p2, and p3 represented initial QTL from population 220 × PH4CV, 220 × Y1518, and P9-10 × PH4CV, respectively. ^*c*^Reference indicted that the mQTL overlapped with QTL identified in this study.*

*mQTL2-1* region (position 194–199 Mb on chromosome 2) contained two germination specific QTL with a GI QTL from 220 × PH4CV and an ER QTL from 220 × Y1518 (**Table 3**). The favorable alleles for both QTL were inherited from the tolerant line 220. However, the explained phenotypic variance differed substantially with 18.21% (GI) and 4.81% (ER) explained phenotype variance, respectively.

### Quantitative PCR Validation for Candidate Genes

**FIGURE 4:**
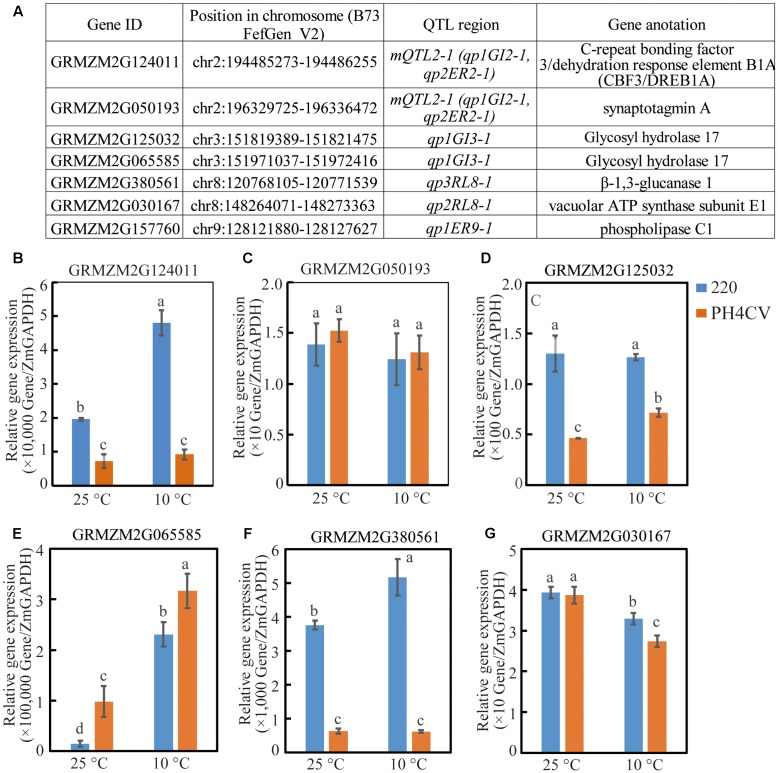
Expressions of six candidate genes in parent lines 220 and PH4CV. Expression were conducted on shoots that were collected at 15 days after sowing (DAS) and 3 DAS under low (10°C) and normal (25°C) temperature, respectively. **(A)** Candidate genes and their chromosome locations. **(B–G)** Expression for different gene.

The six candidate genes within the above detected QTL were homologous to published low-temperature adaption genes by BLAST analysis (Supplementary Tables S2, S5). Among that, three genes (GRMZM2G124011, GRMZM2G380561, and GRMZM2G125032) showed significant higher expression levels in 220 than in PH4CV in any conditions (both optimal- and low-temperatures), with that two genes (GRMZM2G124011 and GRMZM2G380561) in 220 were induced by cold stress, while in PH4CV, gene GRMZM2G125032 was induced (**Figures 4B,D,F**). Interestingly, gene GRMZM2G030167 showed significant higher expression in 220 than in PH4CV only under low-temperature condition, while showed similar expression level under optimal temperature (**Figure 4G**). There is no expression difference of the gene GRMZM2G050193 across the two parents and at the two germination conditions (**Figure 4C**). The last gene GRMZM2G065585 was a possible non-functional gene during seed germination since its expression level is 1/1,000 less than its homolog GRMZM2G125032 in the investigated tissues (**Figure 4E**).

## Discussion

### Important mQTL for Controlling Low-Temperature Germination Ability in Maize

Improvement of maize LTGA could help farmers to sow early, which has advantages in earlier harvest and longer plant life cycle: earlier harvest could extend maize growth region to higher altitudes and longer life cycle increases biomass accumulation and yield output (Frascaroli and Landi, 2017). Although seed LTGA could be improved by agronomic practices, such as seed priming or plastic sheeting (Li et al., 2017; Sheteiwy et al., 2017), the most economical and reliable method is to breed low-temperature tolerance cultivars aided by marker assisted selection (MAS). Recently, researchers identified various QTL associated with low temperature tolerance in maize (Fracheboud et al., 2004; Presterl et al., 2007; Rodriguez et al., 2008; Hu et al., 2016; Shi et al., 2016), but little is known about germination stage. Up to know, no LTGA QTL has been fine-mapped or cloned in maize. The major aim of this study is try to detect the most promising mQTL for further gene cloning and MAS.

To evaluate the reliability of QTL detected in this work, we compared our identified 43 QTL to the previous QTL released from several cold related publications in maize. According to the physical position of QTL on B73 RefGen_v2, we found that 29 QTL (67.4%) overlapped with published QTL (Supplementary Table S5). Among them six QTL (*qp3TL9-1*, *qp3SL9-1*, *qp2ER2-1*, *qp1GI2-1*, *qp1ER9-1*, and *qp1GI3-1*) overlapped with previous QTL at germination and seedling stage (Huang et al., 2013; Hu et al., 2016; Revilla et al., 2016). These results suggest that QTL detected in our study are highly reliable when used for gene cloning and MAS. In addition, a high frequency of germination QTL associated with QTL for other growth stages in diverse genetic backgrounds indicates that maize has a relatively conservative mechanism to adapt to suboptimal temperatures.

Three mQTL were found to locate in chromosome regions that have been mentioned to harbor cold related QTL in previous reports (**Table 3**). The region of *mQTL1-1* included seven initial QTL associated with four traits (ER, TL, RL, and SL) in population 220 × PH4CV and P9-10 × PH4CV, with three QTL explaining more than 30% of phenotypic variance (Supplementary Table S5). In previous studies, some QTL have also been identified in this region for cold related traits by using different populations. Fracheboud et al. (2002) identified a QTL for CO_2_ fixation and PSII of leaves at 15°C from a RIL population; Allam et al. (2016) identified a QTL associated ear height of mature plant under low temperature from another RIL population; and Hund et al. (2004) identified a root trait QTL using maize seedlings grown at low temperatures. These results indicate that this region might harbor a major gene or several effective genes with pleiotropic effect on maize low temperature response at different growth stages.

*mQTL9-1* harbored three initial QTL for seedling traits. Although the explained phenotypic variances of QTL within *mQTL9-1* were low (3.02–5.04%), this region was confirmed to harbor consistently expressed QTL at low temperatures during germination, seedling and maturity stage (Supplementary Table S5). Hu et al. (2016) found two QTL controlling seed germination rate and seedling primary RL from the B73 × Mo17 population, with favor alleles inherited from cold tolerant line Mo17. Besides germination traits, Allam et al. (2016) identified two QTL to control plant height and 100 kernel weight from populations B73 × P39 and B73 × IL14h under low temperature, with trait-increasing alleles inherited from cold tolerant line P39 and IL14h, respectively. Jompuk et al. (2005) detected a QTL in this region controlling PSII content of plant under early sowing (low temperature during early spring). The explained phenotypic variance of QTL in *mQTL9-1* region varied from moderate in Allam et al. (2016) (13.8–18.6%) and in Jompuk et al. (2005) (3.6–24.8%) to low in Hu et al. (2016) (5.25–8.41%). These results suggest that *mQTL9-1* region also contain an important QTL for cold response, which might function at multiple growth stage and exist in multiple genetic background in maize.

*mQTL2-1* harbored two initial QTL detected from populations 220 × PH4CV and 220 × Y1518 for germination traits. Trait-increasing alleles were both inherited from 220, suggesting that *mQTL2-1* allele from 220 could function in different genetic background. Although in this region there is no cold related QTL reported from bi-parental population, an SNP marker linked with a cold associated QTL (response for relative TL, the ratio of total seedling length measured under chilling stress and normal condition) was revealed from a genome wide associate study (GWAS, Huang et al., 2013). Moreover, a cold induced CBF3/DREB1A transcript factor, GRMZM2G124011, was identified to locate in this region (Maruyama et al., 2004; Chinnusamy et al., 2007). This gene showed higher expression level in tolerant line 220 than in susceptible line PH4CV, suggesting that GRMZM2G124011 might be a candidate gene in *mQTL2-1* region responding for the different cold tolerance between 220 and PH4CV (**Figure 4B**).

Quantitative trait locus with higher effect are generally easier for gene cloning or more efficient for MAS (Moreau et al., 2004; Stromberg et al., 1994; Asea et al., 2012). In this work, 16 QTL had phenotypic variance higher than 10% (Supplementary Table S5). Of them, eight belonged to single-population that located in chromosome regions other than the mQTL intervals. *qp2RL8-1* and *qp2RL8-2* explained 10.44–17.97% phenotype variations, and located in a neighboring region on Chr 8: 146.99–153.79 Mb, a region overlapped by the previously reported QTL which associated to maize seedling photosynthesis at chill condition (Jompuk et al., 2005). In addition, the gene GRMZM2G030167, encoding a vacuolar ATP synthase subunit E1, located in this QTL interval and showed higher expression in 220 than in PH4CV (**Figure 4** and Schulze et al., 2012; Zhang Z. et al., 2017). And the expression of GRMZM2G030167 in both parents decreased under cold stress (**Figure 4G**). Comparing to PH4CV, the slower decrease of *GRMZM2G030167* expression in 220 might be a reason responding for its higher cold tolerance that was achieved by maintaining a higher ATP metabolism level in 220 under cold stress. *qp2ER9-1* and *qp2GI9-1* were overlapping on Chr 9: 94.81–96.00 Mb, and explained phenotype variation of 13.72 and 21.46%, respectively (Supplementary Table S5). Of which QTL region, Leipner et al. (2008) detected a QTL responding for dry weight of ear that were subjected to chill stress during seedling stage. *qp2SL2-1* explained 12.31% phenotypic variance, and located on Chr 2: 190.93–191.82 Mb. This QTL region was overlapped with an identified QTL of controlling fresh weight grown in chill environment (Presterl et al., 2007). The last three QTL (*qp1ER5-2*, *qp2SL3-1*, and *qp3ER10-1*) with high-effect were new discovered QTL where no genes or QTL was reported in previously works (Supplementary Table S5).

### Prospects for Gene Cloning and Marker-Assisted Selection for Low-Temperature Germination Ability in Maize

The reliability of QTL analysis depends on population size, phenotypic variance, phenotyping methods and marker density, etc. In this study, we addressed some of these obstacles and detected the most promising mQTL for further gene cloning and MAS. First, we applied two different methods (sand bed and paper rolls) for germination trait evaluation at low temperature, in contrast to previous studies that only used one of the methods of sand bed, peat bed, paper rolls or field evaluation at early spring (Hund et al., 2004; Rodriguez et al., 2014; Hu et al., 2016; Shi et al., 2016). We found significant correlations between traits collected from different methods, suggesting that QTL repeatedly detected by two methods might be more reliable for further gene cloning and MAS.

Second, use of three populations for phenotyping and genotyping enabled us to identify mQTL across populations. Although a number of QTL associated with cold acclimation have been identified, only few were consistent across diverse genetic backgrounds. In this work, three mQTL were identified to contain initial QTL from two populations. Furthermore, two of the identified mQTL (*mQTL1-1* and *mQTL2-1*) included initial QTL with high explained phenotypic variance, making these mQTL attractive for gene cloning and MAS.

Third, outstanding parental lines with contrasting cold tolerance were selected for generating populations, which contributed to efficient identification of major QTL. Of the 43 initial QTL, 3 explained more than 30% and 14 explained 10–30% phenotypic variance. Cold tolerance increasing alleles were all inherited from the tolerant lines 220 or P9-10, further supporting the efficacy of selection of suitable parental lines. In contrast, phenotypic variance of QTL identified in previous reports were generally lower than 20% (Hund et al., 2004; Leipner et al., 2008; Rodriguez et al., 2014; Hu et al., 2016; Shi et al., 2016).

## Conclusion

We identified 43 QTL responsible for maize LTGA using three connected populations germinated in a sand bed and paper rolls. By constructing a consensus linkage map, three mQTL were suggested to include initial QTL that detected from different populations. In future, it is of great interest to clone genes underlying mQTL regions and uncover the molecular mechanisms of maize cold tolerance during germination.

## Author Contributions

GyW, JW, and RG designed the study; XL, GhW, GJ, LR, and LL performed the experiments; XL and JF analyzed the data; XL drafted the manuscript; TL, JW, and RG advised on data analysis and revised the manuscript.

## Conflict of Interest Statement

The authors declare that the research was conducted in the absence of any commercial or financial relationships that could be construed as a potential conflict of interest.

## Footnotes

1 http://maizegdb.org/
