# Supplementary material for: QTL Mapping in Three Connected Populations Reveals a Set of Consensus Genomic Regions for Low Temperature Germination Ability in Zea mays L
Source: Front Plant Sci. 2018 Jan 31;9:65. doi: 10.3389/fpls.2018.00065 (PMC5797882; doi:10.3389/fpls.2018.00065)
Supplement: Supplementary file 1 [file Data_Sheet_1.DOCX]

**Supplementary data**

**QTL mapping in three connected populations reveals a set of consensus genomic regions for low temperature germination ability in *Zea mays* L.**

Xuhui Li ^1^, Guihua Wang ^1^, Junjie Fu ^2^, Li Li ^1^, Guangyao Jia ^1^, Lisha Ren ^1^, Thomas Lubberstedt ^3^, Guoying Wang ^2^, Jianhua Wang ^1*^, Riliang Gu ^1*^

^1^ Center of Seed Science and Technology, Beijing Key Laboratory of Crop Genetics and Breeding, Innovation Center for Seed Technology (MOA), China Agricultural University, Beijing 100193, China.

^2^ Institute of Crop Sciences, Chinese Academy of Agricultural Sciences, Beijing 100081, China

^3^ Dept. of Agronomy, Iowa State University, 1204 Agronomy Hall, Ames, Iowa 50011, USA

*Correspondence: Jianhua Wang ([wangjh63@cau.edu.cn](mailto:wangjh63@cau.edu.cn)) or Riliang Gu ([rilianggu@cau.edu.cn](mailto:rilianggu@cau.edu.cn))

**Supplementary Table S1. LOD threshold value for emergence rate (ER), germination index (GI), total length (TL), root length (RL) and shoot length (SL) in population 220 × PH4CV, 220 × Y1518 and P9-10 × PH4CV.**

ER and GI were collected from sand bed experiment, and TL, RL and SL were collected from paper rolls experiment. QTL analyses were conducted by inclusive composite interval mapping (ICIM) mapping using IciMapping 4.1 (Zhang *et al*. 2008). Forward regression was performed using a walking speed of 1 cM. The threshold LOD value was determined with 1000 permutations at P=0.05 level for each trait (Churchill and Doerge 1994).

|  | ER | GI | TL | RL | SL |
| --- | --- | --- | --- | --- | --- |
| 220 × PH4CV | 5.28 | 3.86 | 3.79 | 3.85 | 3.70 |
| 220 × Y1518 | 4.56 | 3.95 | 4.10 | 4.09 | 3.81 |
| P9-10 × PH4CV | 6.83 | 4.25 | 3.98 | 3.85 | 3.82 |

References

Churchill, GA., Doerge, RW. (1994). Empirical threshold values for quantitative trait mapping. Genetics 138:963-971

Zhang, L., Li, H., Li, Z., Wang, J. (2008). Interactions between markers can be caused by the dominance effect of quantitative trait loci. Genetics 180:1177-1190. doi: 10.1534/genetics.108.092122

**Supplementary Table S2. Candidate gene predicted to locate within the 43 QTL regions and the corresponding qPCR primers.**

| Gene ID | Position in chromosome (B73 FefGen_V2)^a^ | Ortholog in Arabidopsis and Rice | Gene anotation | QTL region | Reference | Primer F (5'-3') | Primer R (5'-3') |
| --- | --- | --- | --- | --- | --- | --- | --- |
| GRMZM2G124011 | chr2:194485273-194486255 | AT4G25480.1;  LOC_Os09g35030.1 | CBF/DREB1A | *mQTL2-1 (qp1GI2-1,  qp2ER2-1)* | Maruyama et al., 2004; Chinnusamy et al., 2007. | TCATGGCCAGTTTTGCTCTG | ACAGGCGGAGTTCCTTATGG |
| GRMZM2G050193 | chr2:196329725-196336472 | AT2G20990.1;  LOC_Os09g36770.1 | synaptotagmin A | *mQTL2-1 (qp2ER2-1)* | Yamazaki et al., 2008. | AGCACCATACAAACCCATATGC | CTTGTTGCTGATCACGTCTG |
| GRMZM2G125032 | chr3:151819389-151821475 | AT4G16260.1; LOC_Os01g71670.1 | Glycosyl hydrolase 17 | *qp1GI3-1* | Oono et al., 2006; Moellering et al., 2010; Legrand et al., 2013. | GTCCCAGTCGTCGTGTCC | CGATTTGTCCGGGTTGAAGA |
| GRMZM2G065585 | chr3:151971037-151972416 | AT4G16260.1;  LOC_Os01g71340.1 | Glycosyl hydrolase 17 | *qp1GI3-1* | Oono et al., 2006; Moellering et al., 2010; Legrand et al., 2013. | GACAACGCGCGGAAATACAA | CTCCGATCCGCCACTCTTAA |
| GRMZM2G380561 | chr8:120768105-120771539 | AT3G57270.1;  LOC_Os05g41610.1 | β-1,3-glucanase 1 | *qp3RL8-1* | Hincha et al., 1997. | ACTTCGGCCTCTTCCAGC | TGCTACTCCAAGTCTCCAACA |
| GRMZM2G030167 | chr8:148264071-148273363 | AT4G11150.1;  LOC_Os01g46980.1 | vacuolar ATP synthase subunit E1 | *qp2RL8-1* | Schulze et al., 2012; Zhang et al., 2017. | GTGCACGAGTCGGACAAC | GTGGAGTTGGAGTCGGAGG |
| GRMZM2G157760 | chr9:128121880-128127627 | AT5G58670.1;  LOC_Os05g03610.1 | phospholipase C1 | *qp1ER9-1* | Hirayama et al. 1995; Ruelland et al.2002; Bond et al., 2011. | CTGTACTACGCGAACTTGGC | CCCAATCTGCTCTGTTTCGT |
| GRMZM2G046804 |  | Reference gene | gpc1 - glyceraldehyde-3-phosphate dehydrogenase1 |  |  | CTGGTTTCTACCGACTTCCTTG | CGGCATACACAAGCAGCAAC |

**Reference**

Bond, D. M., Dennis, E. S., and Finnegan, E. (2011). The low temperature response pathways for cold acclimation and vernalization are independent. *Plant, cell & environment* 34, 1737-1748. doi: 10.1111/j.1365-3040.2011.02370.x

Chinnusamy, V., Zhu, J., and Zhu, J. K. (2007). Cold stress regulation of gene expression in plants. *Trends in plant science* 12, 444-451. doi: 10.1016/j.tplants.2007.07.002

Hincha, D. K., Meins Jr, F., and Schmitt, J. M. (1997). β-1, 3-Glucanase is cryoprotective in vitro and is accumulated in leaves during cold acclimation. *Plant Physiology* 114, 1077-1083. doi: 10.1104/pp.114.3.1077

Hirayama, T., Ohto, C., Mizoguchi, T., and Shinozaki, K. (1995). A gene encoding a phosphatidylinositol-specific phospholipase C is induced by dehydration and salt stress in Arabidopsis thaliana. *Proceedings of the National Academy of Sciences* 92, 3903-3907. doi: 10.1073/pnas.92.9.3903

Legrand, S., Marque, G., Blassiau, C., Bluteau, A., Canoy, A. S., Fontaine, V. et al., (2013). Combining gene expression and genetic analyses to identify candidate genes involved in cold responses in pea. *Journal of plant physiology* 170, 1148-1157. doi: 10.1016/j.jplph.2013.03.014

Maruyama, K., Sakuma, Y., Kasuga, M., Ito, Y., Seki, M., and Goda, H., et al. (2010). Identification of cold-inducible downstream genes of the arabidopsis dreb1a/cbf3 transcriptional factor using two microarray systems. *Plant Journal for Cell & Molecular Biology* 38, 982-993. doi: 10.1111/j.1365-313X.2004.02100.x

Moellering, E. R., Muthan, B., and Benning, C. (2010). Freezing tolerance in plants requires lipid remodeling at the outer chloroplast membrane. *Science* 330, 226-228. doi: 10.1126/science.1191803

Oono, Y., Seki, M., Satou, M., Iida, K., Akiyama, K., Sakurai, T., et al (2006). Monitoring expression profiles of Arabidopsis genes during cold acclimation and deacclimation using DNA microarrays. *Functional & integrative genomics* 6, 212-234. doi: 10.1007/s10142-005-0014-z

Ruelland, E., Cantrel, C., Gawer, M., Kader, J. C., and Zachowski, A. (2002). Activation of phospholipases C and D is an early response to a cold exposure in Arabidopsis suspension cells. *Plant physiology* 130, 999-1007. doi: 10.1104/pp.006080

Schulze, W. X., Schneider, T., Starck, S., Martinoia, E., and Trentmann, O. (2012). Cold acclimation induces changes in Arabidopsis tonoplast protein abundance and activity and alters phosphorylation of tonoplast monosaccharide transporters. *The Plant Journal* 69, 529-541. doi: 10.1111/j.1365-313X.2011.04812.x

Yamazaki, T., Kawamura, Y., Minami, A., and Uemura, M. (2008). Calcium-dependent freezing tolerance in Arabidopsis involves membrane resealing via synaptotagmin SYT1. *The Plant Cell* 20, 3389-3404. doi: 10.1105/tpc.108.062679

Zhang, Z., Li, J., Pan, Y., Li, J., Zhou, L., Shi, H., *et al.,* (2017b). Natural variation in CTB4a enhances rice adaptation to cold habitats. *Nat. Commun.* 8, 14788. doi: 10.1038/ncomms14788

**Supplementary Table S3. SNP name, position and allele information listed in the 6K SNP chip.**

| SNP name | Chrompsome | Position in B73 RefGen_v2 | Allele |
| --- | --- | --- | --- |
| M1c3498 | 1 | 3498 | [T/C] |
| M1c526900 | 1 | 526900 | [T/G] |
| M1c983240 | 1 | 983240 | [A/C] |
| M1c1167803 | 1 | 1167803 | [T/C] |
| M1c1491139 | 1 | 1491139 | [A/C] |
| M1c2087962 | 1 | 2087962 | [T/G] |
| M1c2742603 | 1 | 2742603 | [A/C] |
| M1c3078257 | 1 | 3078257 | [T/G] |
| M1c3138346 | 1 | 3138346 | [A/G] |
| M1c3192090 | 1 | 3192090 | [T/C] |
| M1c3468972 | 1 | 3468972 | [A/C] |
| M1c4307186 | 1 | 4307186 | [A/G] |
| M1c4764981 | 1 | 4764981 | [T/C] |
| M1c4983841 | 1 | 4983841 | [T/C] |
| M1c5391225 | 1 | 5391225 | [T/G] |
| M1c5628808 | 1 | 5628808 | [T/C] |
| M1c6171432 | 1 | 6171432 | [A/G] |
| M1c6243881 | 1 | 6243881 | [T/G] |
| M1c6843871 | 1 | 6843871 | [A/G] |
| M1c7198437 | 1 | 7198437 | [T/G] |
| M1c7296984 | 1 | 7296984 | [A/G] |
| M1c7601919 | 1 | 7601919 | [T/C] |
| M1c7955274 | 1 | 7955274 | [T/C] |
| M1c8267250 | 1 | 8267250 | [T/C] |
| M1c8633823 | 1 | 8633823 | [T/C] |
| M1c9190800 | 1 | 9190800 | [A/G] |
| M1c9363075 | 1 | 9363075 | [A/G] |
| M1c9912112 | 1 | 9912112 | [T/C] |
| M1c10094942 | 1 | 10094942 | [A/G] |
| M1c10588984 | 1 | 10588984 | [T/C] |
| M1c10675406 | 1 | 10675406 | [A/G] |
| M1c10992454 | 1 | 10992454 | [A/G] |
| M1c11488397 | 1 | 11488397 | [T/C] |
| M1c11911538 | 1 | 11911538 | [T/C] |
| M1c12189099 | 1 | 12189099 | [T/C] |
| M1c12353603 | 1 | 12353603 | [A/G] |
| M1c12950549 | 1 | 12950549 | [T/C] |
| M1c13210879 | 1 | 13210879 | [A/G] |
| M1c13645072 | 1 | 13645072 | [T/C] |
| M1c13993255 | 1 | 13993255 | [T/C] |
| M1c14626607 | 1 | 14626607 | [T/C] |
| M1c15069918 | 1 | 15069918 | [T/C] |
| M1c15356376 | 1 | 15356376 | [A/G] |
| M1c15776215 | 1 | 15776215 | [A/G] |
| M1c15992182 | 1 | 15992182 | [A/G] |
| M1c16335539 | 1 | 16335539 | [A/G] |
| M1c16777255 | 1 | 16777255 | [A/G] |
| M1c16953648 | 1 | 16953648 | [T/C] |
| M1c17494038 | 1 | 17494038 | [A/C] |
| M1c17714709 | 1 | 17714709 | [A/G] |
| M1c17882976 | 1 | 17882976 | [A/G] |
| M1c18202377 | 1 | 18202377 | [A/G] |
| M1c20106697 | 1 | 20106697 | [T/C] |
| M1c20420587 | 1 | 20420587 | [T/C] |
| M1c20915191 | 1 | 20915191 | [A/G] |
| M1c21251491 | 1 | 21251491 | [T/G] |
| M1c21468153 | 1 | 21468153 | [A/C] |
| M1c21758216 | 1 | 21758216 | [A/G] |
| M1c21984239 | 1 | 21984239 | [A/G] |
| M1c22512578 | 1 | 22512578 | [T/C] |
| M1c22649836 | 1 | 22649836 | [T/C] |
| M1c23188850 | 1 | 23188850 | [A/G] |
| M1c23566918 | 1 | 23566918 | [T/C] |
| M1c23967012 | 1 | 23967012 | [T/C] |
| M1c24203717 | 1 | 24203717 | [A/C] |
| M1c24510735 | 1 | 24510735 | [A/G] |
| M1c24736709 | 1 | 24736709 | [T/G] |
| M1c25400235 | 1 | 25400235 | [A/G] |
| M1c25882835 | 1 | 25882835 | [A/G] |
| M1c26201897 | 1 | 26201897 | [T/C] |
| M1c26680358 | 1 | 26680358 | [A/G] |
| M1c27101738 | 1 | 27101738 | [T/C] |
| M1c27305274 | 1 | 27305274 | [A/G] |
| M1c27555261 | 1 | 27555261 | [T/C] |
| M1c27911882 | 1 | 27911882 | [T/C] |
| M1c28421841 | 1 | 28421841 | [T/C] |
| M1c28525652 | 1 | 28525652 | [T/C] |
| M1c28967906 | 1 | 28967906 | [T/C] |
| M1c29197802 | 1 | 29197802 | [A/G] |
| M1c29542872 | 1 | 29542872 | [T/C] |
| M1c29966522 | 1 | 29966522 | [T/C] |
| M1c30346233 | 1 | 30346233 | [A/G] |
| M1c30688527 | 1 | 30688527 | [A/G] |
| M1c30906936 | 1 | 30906936 | [T/C] |
| M1c31378717 | 1 | 31378717 | [T/G] |
| M1c31742362 | 1 | 31742362 | [T/C] |
| M1c32157097 | 1 | 32157097 | [A/G] |
| M1c32579627 | 1 | 32579627 | [A/C] |
| M1c32871094 | 1 | 32871094 | [T/C] |
| M1c33216909 | 1 | 33216909 | [T/C] |
| M1c33408781 | 1 | 33408781 | [A/G] |
| M1c33824383 | 1 | 33824383 | [T/C] |
| M1c34155301 | 1 | 34155301 | [A/G] |
| M1c34386853 | 1 | 34386853 | [T/C] |
| M1c34716138 | 1 | 34716138 | [A/G] |
| M1c35134483 | 1 | 35134483 | [T/C] |
| M1c35455443 | 1 | 35455443 | [A/G] |
| M1c35935970 | 1 | 35935970 | [A/G] |
| M1c36216181 | 1 | 36216181 | [T/C] |
| M1c36499105 | 1 | 36499105 | [A/C] |
| M1c36800951 | 1 | 36800951 | [T/G] |
| M1c37202160 | 1 | 37202160 | [T/C] |
| M1c37717756 | 1 | 37717756 | [A/G] |
| M1c37842905 | 1 | 37842905 | [T/G] |
| M1c38315375 | 1 | 38315375 | [A/C] |
| M1c38550339 | 1 | 38550339 | [T/G] |
| M1c39057809 | 1 | 39057809 | [T/G] |
| M1c39404867 | 1 | 39404867 | [A/C] |
| M1c39743117 | 1 | 39743117 | [T/G] |
| M1c39843836 | 1 | 39843836 | [A/G] |
| M1c40224985 | 1 | 40224985 | [T/C] |
| M1c40812988 | 1 | 40812988 | [A/G] |
| M1c41104295 | 1 | 41104295 | [T/C] |
| M1c41237489 | 1 | 41237489 | [T/C] |
| M1c41964754 | 1 | 41964754 | [T/C] |
| M1c42522289 | 1 | 42522289 | [A/G] |
| M1c42851889 | 1 | 42851889 | [T/C] |
| M1c42922738 | 1 | 42922738 | [A/C] |
| M1c43421131 | 1 | 43421131 | [T/G] |
| M1c43681143 | 1 | 43681143 | [T/C] |
| M1c44161458 | 1 | 44161458 | [A/G] |
| M1c44348872 | 1 | 44348872 | [A/G] |
| M1c44802580 | 1 | 44802580 | [A/G] |
| M1c45102852 | 1 | 45102852 | [T/C] |
| M1c45338592 | 1 | 45338592 | [A/C] |
| M1c45778940 | 1 | 45778940 | [A/G] |
| M1c46232564 | 1 | 46232564 | [A/G] |
| M1c46622962 | 1 | 46622962 | [T/C] |
| M1c47301627 | 1 | 47301627 | [T/G] |
| M1c47506790 | 1 | 47506790 | [T/C] |
| M1c47901197 | 1 | 47901197 | [A/G] |
| M1c48659763 | 1 | 48659763 | [A/G] |
| M1c48877642 | 1 | 48877642 | [T/C] |
| M1c49392023 | 1 | 49392023 | [A/G] |
| M1c49726574 | 1 | 49726574 | [T/C] |
| M1c50096448 | 1 | 50096448 | [A/C] |
| M1c50439401 | 1 | 50439401 | [T/C] |
| M1c51046985 | 1 | 51046985 | [A/C] |
| M1c52085125 | 1 | 52085125 | [A/C] |
| M1c52227535 | 1 | 52227535 | [T/C] |
| M1c52772482 | 1 | 52772482 | [A/C] |
| M1c52920957 | 1 | 52920957 | [A/G] |
| M1c53345008 | 1 | 53345008 | [A/G] |
| M1c53947584 | 1 | 53947584 | [T/C] |
| M1c54275745 | 1 | 54275745 | [T/G] |
| M1c55163699 | 1 | 55163699 | [T/C] |
| M1c55380139 | 1 | 55380139 | [T/C] |
| M1c55739262 | 1 | 55739262 | [T/C] |
| M1c56224437 | 1 | 56224437 | [T/C] |
| M1c56558305 | 1 | 56558305 | [T/C] |
| M1c56786832 | 1 | 56786832 | [T/C] |
| M1c57246788 | 1 | 57246788 | [T/C] |
| M1c57349324 | 1 | 57349324 | [A/C] |
| M1c57835920 | 1 | 57835920 | [A/G] |
| M1c58056224 | 1 | 58056224 | [A/G] |
| M1c58577657 | 1 | 58577657 | [T/C] |
| M1c59463070 | 1 | 59463070 | [T/G] |
| M1c59863674 | 1 | 59863674 | [T/C] |
| M1c60262719 | 1 | 60262719 | [T/C] |
| M1c60478149 | 1 | 60478149 | [T/G] |
| M1c60832251 | 1 | 60832251 | [A/C] |
| M1c61137029 | 1 | 61137029 | [A/C] |
| M1c61449224 | 1 | 61449224 | [T/C] |
| M1c62714138 | 1 | 62714138 | [A/G] |
| M1c63044321 | 1 | 63044321 | [A/G] |
| M1c63358665 | 1 | 63358665 | [T/G] |
| M1c64004661 | 1 | 64004661 | [A/C] |
| M1c64392596 | 1 | 64392596 | [T/C] |
| M1c64542686 | 1 | 64542686 | [T/C] |
| M1c65089709 | 1 | 65089709 | [T/C] |
| M1c65488776 | 1 | 65488776 | [T/C] |
| M1c66014068 | 1 | 66014068 | [A/G] |
| M1c66399512 | 1 | 66399512 | [A/G] |
| M1c66769981 | 1 | 66769981 | [A/C] |
| M1c67525919 | 1 | 67525919 | [T/C] |
| M1c67773150 | 1 | 67773150 | [T/C] |
| M1c68106189 | 1 | 68106189 | [T/C] |
| M1c68439791 | 1 | 68439791 | [A/G] |
| M1c68663634 | 1 | 68663634 | [A/G] |
| M1c69009439 | 1 | 69009439 | [A/G] |
| M1c69541789 | 1 | 69541789 | [A/G] |
| M1c70250834 | 1 | 70250834 | [T/G] |
| M1c70518275 | 1 | 70518275 | [A/G] |
| M1c70725221 | 1 | 70725221 | [T/C] |
| M1c71583778 | 1 | 71583778 | [A/G] |
| M1c71794734 | 1 | 71794734 | [T/G] |
| M1c72097319 | 1 | 72097319 | [T/C] |
| M1c72618382 | 1 | 72618382 | [A/G] |
| M1c73637089 | 1 | 73637089 | [T/C] |
| M1c74415285 | 1 | 74415285 | [T/C] |
| M1c74487925 | 1 | 74487925 | [T/C] |
| M1c74959560 | 1 | 74959560 | [T/G] |
| M1c75156367 | 1 | 75156367 | [A/G] |
| M1c75759490 | 1 | 75759490 | [A/G] |
| M1c75963363 | 1 | 75963363 | [A/G] |
| M1c76610535 | 1 | 76610535 | [T/C] |
| M1c76881598 | 1 | 76881598 | [T/C] |
| M1c77243410 | 1 | 77243410 | [T/C] |
| M1c77797955 | 1 | 77797955 | [T/C] |
| M1c78027428 | 1 | 78027428 | [A/G] |
| M1c78289456 | 1 | 78289456 | [T/C] |
| M1c78707555 | 1 | 78707555 | [A/G] |
| M1c79236413 | 1 | 79236413 | [A/G] |
| M1c79261683 | 1 | 79261683 | [T/G] |
| M1c79707748 | 1 | 79707748 | [A/G] |
| M1c80058364 | 1 | 80058364 | [T/G] |
| M1c80321796 | 1 | 80321796 | [A/G] |
| M1c81212962 | 1 | 81212962 | [A/G] |
| M1c81640277 | 1 | 81640277 | [A/G] |
| M1c82015054 | 1 | 82015054 | [T/C] |
| M1c82580634 | 1 | 82580634 | [A/G] |
| M1c82702375 | 1 | 82702375 | [T/C] |
| M1c83281160 | 1 | 83281160 | [T/C] |
| M1c83404103 | 1 | 83404103 | [A/G] |
| M1c84295709 | 1 | 84295709 | [T/C] |
| M1c84567674 | 1 | 84567674 | [A/G] |
| M1c85060051 | 1 | 85060051 | [A/G] |
| M1c85277408 | 1 | 85277408 | [T/G] |
| M1c85732983 | 1 | 85732983 | [A/G] |
| M1c85834569 | 1 | 85834569 | [T/C] |
| M1c86168856 | 1 | 86168856 | [T/C] |
| M1c86590155 | 1 | 86590155 | [T/C] |
| M1c86945879 | 1 | 86945879 | [A/G] |
| M1c87439599 | 1 | 87439599 | [A/G] |
| M1c87562343 | 1 | 87562343 | [A/C] |
| M1c87916466 | 1 | 87916466 | [T/G] |
| M1c88323086 | 1 | 88323086 | [A/G] |
| M1c88530879 | 1 | 88530879 | [T/C] |
| M1c88887675 | 1 | 88887675 | [A/G] |
| M1c89830165 | 1 | 89830165 | [T/C] |
| M1c90222759 | 1 | 90222759 | [T/G] |
| M1c90327105 | 1 | 90327105 | [A/G] |
| M1c90820362 | 1 | 90820362 | [A/G] |
| M1c91188438 | 1 | 91188438 | [A/C] |
| M1c91461737 | 1 | 91461737 | [T/G] |
| M1c91765543 | 1 | 91765543 | [T/C] |
| M1c92241184 | 1 | 92241184 | [T/C] |
| M1c92464862 | 1 | 92464862 | [A/C] |
| M1c92663680 | 1 | 92663680 | [T/C] |
| M1c93077115 | 1 | 93077115 | [T/C] |
| M1c93505861 | 1 | 93505861 | [T/C] |
| M1c93801529 | 1 | 93801529 | [T/C] |
| M1c94013486 | 1 | 94013486 | [A/G] |
| M1c94469030 | 1 | 94469030 | [A/G] |
| M1c94822341 | 1 | 94822341 | [A/G] |
| M1c95189882 | 1 | 95189882 | [T/G] |
| M1c95659376 | 1 | 95659376 | [T/C] |
| M1c95894883 | 1 | 95894883 | [T/C] |
| M1c96114507 | 1 | 96114507 | [T/G] |
| M1c96683443 | 1 | 96683443 | [T/C] |
| M1c96811016 | 1 | 96811016 | [A/G] |
| M1c97152383 | 1 | 97152383 | [T/C] |
| M1c97679432 | 1 | 97679432 | [A/C] |
| M1c97885478 | 1 | 97885478 | [T/C] |
| M1c98355583 | 1 | 98355583 | [A/G] |
| M1c98547234 | 1 | 98547234 | [T/C] |
| M1c99134559 | 1 | 99134559 | [T/C] |
| M1c99697241 | 1 | 99697241 | [T/C] |
| M1c99846039 | 1 | 99846039 | [A/G] |
| M1c100825561 | 1 | 100825561 | [T/G] |
| M1c100907218 | 1 | 100907218 | [T/C] |
| M1c101338083 | 1 | 101338083 | [T/C] |
| M1c101684363 | 1 | 101684363 | [A/G] |
| M1c102090732 | 1 | 102090732 | [A/G] |
| M1c102711899 | 1 | 102711899 | [T/G] |
| M1c102950232 | 1 | 102950232 | [A/G] |
| M1c103433384 | 1 | 103433384 | [T/C] |
| M1c103826700 | 1 | 103826700 | [A/C] |
| M1c104276740 | 1 | 104276740 | [T/C] |
| M1c104522780 | 1 | 104522780 | [A/G] |
| M1c105153661 | 1 | 105153661 | [A/G] |
| M1c105471290 | 1 | 105471290 | [T/C] |
| M1c105951445 | 1 | 105951445 | [T/C] |
| M1c106159787 | 1 | 106159787 | [A/G] |
| M1c106668929 | 1 | 106668929 | [A/G] |
| M1c106906318 | 1 | 106906318 | [T/C] |
| M1c107311994 | 1 | 107311994 | [A/C] |
| M1c107449508 | 1 | 107449508 | [T/C] |
| M1c108067006 | 1 | 108067006 | [T/C] |
| M1c108311453 | 1 | 108311453 | [A/C] |
| M1c108621763 | 1 | 108621763 | [A/C] |
| M1c108805902 | 1 | 108805902 | [A/G] |
| M1c109389543 | 1 | 109389543 | [A/C] |
| M1c109636752 | 1 | 109636752 | [A/C] |
| M1c109839058 | 1 | 109839058 | [A/C] |
| M1c110251408 | 1 | 110251408 | [T/G] |
| M1c110639058 | 1 | 110639058 | [T/C] |
| M1c111125276 | 1 | 111125276 | [T/C] |
| M1c111219051 | 1 | 111219051 | [T/C] |
| M1c111568941 | 1 | 111568941 | [A/G] |
| M1c112144808 | 1 | 112144808 | [T/C] |
| M1c112334528 | 1 | 112334528 | [A/G] |
| M1c112726993 | 1 | 112726993 | [A/G] |
| M1c113058370 | 1 | 113058370 | [T/C] |
| M1c113529763 | 1 | 113529763 | [T/C] |
| M1c113750078 | 1 | 113750078 | [T/C] |
| M1c114222614 | 1 | 114222614 | [T/C] |
| M1c114285706 | 1 | 114285706 | [T/C] |
| M1c114804544 | 1 | 114804544 | [A/G] |
| M1c115217186 | 1 | 115217186 | [T/C] |
| M1c115338780 | 1 | 115338780 | [A/G] |
| M1c115926398 | 1 | 115926398 | [T/C] |
| M1c116044809 | 1 | 116044809 | [T/C] |
| M1c116332711 | 1 | 116332711 | [A/G] |
| M1c116767275 | 1 | 116767275 | [A/G] |
| M1c117467640 | 1 | 117467640 | [A/G] |
| M1c117714326 | 1 | 117714326 | [A/C] |
| M1c118115744 | 1 | 118115744 | [A/G] |
| M1c118477033 | 1 | 118477033 | [T/C] |
| M1c119023786 | 1 | 119023786 | [T/C] |
| M1c119334206 | 1 | 119334206 | [T/C] |
| M1c119793255 | 1 | 119793255 | [T/C] |
| M1c120365985 | 1 | 120365985 | [T/C] |
| M1c120847600 | 1 | 120847600 | [A/C] |
| M1c121234148 | 1 | 121234148 | [A/G] |
| M1c121487568 | 1 | 121487568 | [A/G] |
| M1c121972604 | 1 | 121972604 | [A/C] |
| M1c122404957 | 1 | 122404957 | [A/G] |
| M1c122797619 | 1 | 122797619 | [T/C] |
| M1c122941186 | 1 | 122941186 | [A/G] |
| M1c123738749 | 1 | 123738749 | [A/C] |
| M1c124127179 | 1 | 124127179 | [A/G] |
| M1c124470353 | 1 | 124470353 | [A/G] |
| M1c124627699 | 1 | 124627699 | [T/C] |
| M1c125727101 | 1 | 125727101 | [A/G] |
| M1c126115863 | 1 | 126115863 | [T/C] |
| M1c127037896 | 1 | 127037896 | [T/C] |
| M1c127290153 | 1 | 127290153 | [T/C] |
| M1c128026259 | 1 | 128026259 | [A/C] |
| M1c128497758 | 1 | 128497758 | [A/G] |
| M1c129219903 | 1 | 129219903 | [A/G] |
| M1c129573485 | 1 | 129573485 | [A/G] |
| M1c129924241 | 1 | 129924241 | [A/G] |
| M1c130666922 | 1 | 130666922 | [A/C] |
| M1c130769889 | 1 | 130769889 | [A/G] |
| M1c131331736 | 1 | 131331736 | [A/G] |
| M1c132171646 | 1 | 132171646 | [A/G] |
| M1c132847804 | 1 | 132847804 | [A/G] |
| M1c133377681 | 1 | 133377681 | [T/C] |
| M1c133468538 | 1 | 133468538 | [A/C] |
| M1c134423696 | 1 | 134423696 | [A/G] |
| M1c135276739 | 1 | 135276739 | [A/G] |
| M1c135562407 | 1 | 135562407 | [T/G] |
| M1c135921281 | 1 | 135921281 | [T/C] |
| M1c136780655 | 1 | 136780655 | [A/G] |
| M1c137455581 | 1 | 137455581 | [T/G] |
| M1c138018387 | 1 | 138018387 | [T/C] |
| M1c138451473 | 1 | 138451473 | [A/G] |
| M1c138612234 | 1 | 138612234 | [T/G] |
| M1c139129124 | 1 | 139129124 | [T/C] |
| M1c139462272 | 1 | 139462272 | [A/G] |
| M1c140039684 | 1 | 140039684 | [A/G] |
| M1c140985509 | 1 | 140985509 | [T/C] |
| M1c141275323 | 1 | 141275323 | [A/G] |
| M1c141534136 | 1 | 141534136 | [T/C] |
| M1c141928718 | 1 | 141928718 | [A/G] |
| M1c142372254 | 1 | 142372254 | [T/C] |
| M1c142704767 | 1 | 142704767 | [T/C] |
| M1c142730310 | 1 | 142730310 | [A/G] |
| M1c144063543 | 1 | 144063543 | [A/C] |
| M1c144231254 | 1 | 144231254 | [A/C] |
| M1c144752244 | 1 | 144752244 | [T/C] |
| M1c145057153 | 1 | 145057153 | [T/C] |
| M1c145317614 | 1 | 145317614 | [A/G] |
| M1c145489078 | 1 | 145489078 | [T/C] |
| M1c146440669 | 1 | 146440669 | [A/G] |
| M1c146506786 | 1 | 146506786 | [A/G] |
| M1c147015971 | 1 | 147015971 | [T/C] |
| M1c147207031 | 1 | 147207031 | [A/G] |
| M1c147709773 | 1 | 147709773 | [A/C] |
| M1c147990047 | 1 | 147990047 | [T/C] |
| M1c148458078 | 1 | 148458078 | [T/G] |
| M1c148628650 | 1 | 148628650 | [A/G] |
| M1c149549096 | 1 | 149549096 | [A/C] |
| M1c149596109 | 1 | 149596109 | [A/G] |
| M1c150106382 | 1 | 150106382 | [A/C] |
| M1c150481698 | 1 | 150481698 | [A/G] |
| M1c150779907 | 1 | 150779907 | [T/C] |
| M1c151023622 | 1 | 151023622 | [T/C] |
| M1c151357947 | 1 | 151357947 | [A/G] |
| M1c151739260 | 1 | 151739260 | [T/C] |
| M1c152164993 | 1 | 152164993 | [T/C] |
| M1c152507712 | 1 | 152507712 | [T/C] |
| M1c152910375 | 1 | 152910375 | [T/C] |
| M1c153343364 | 1 | 153343364 | [A/G] |
| M1c153995388 | 1 | 153995388 | [T/C] |
| M1c154508506 | 1 | 154508506 | [A/C] |
| M1c154954567 | 1 | 154954567 | [T/G] |
| M1c155419043 | 1 | 155419043 | [T/C] |
| M1c155679711 | 1 | 155679711 | [T/C] |
| M1c155933928 | 1 | 155933928 | [A/G] |
| M1c156215727 | 1 | 156215727 | [A/G] |
| M1c156537466 | 1 | 156537466 | [A/G] |
| M1c157138294 | 1 | 157138294 | [A/G] |
| M1c157231910 | 1 | 157231910 | [A/G] |
| M1c157523129 | 1 | 157523129 | [T/C] |
| M1c157907164 | 1 | 157907164 | [A/G] |
| M1c158483447 | 1 | 158483447 | [T/G] |
| M1c158632785 | 1 | 158632785 | [A/G] |
| M1c158923491 | 1 | 158923491 | [T/C] |
| M1c159415824 | 1 | 159415824 | [A/G] |
| M1c159541638 | 1 | 159541638 | [T/C] |
| M1c160119383 | 1 | 160119383 | [T/C] |
| M1c160223741 | 1 | 160223741 | [T/C] |
| M1c160306697 | 1 | 160306697 | [T/C] |
| M1c160572889 | 1 | 160572889 | [A/G] |
| M1c160817659 | 1 | 160817659 | [A/C] |
| M1c160954795 | 1 | 160954795 | [T/C] |
| M1c160954855 | 1 | 160954855 | [A/G] |
| M1c161306894 | 1 | 161306894 | [T/C] |
| M1c161938738 | 1 | 161938738 | [T/C] |
| M1c162267119 | 1 | 162267119 | [A/G] |
| M1c162542042 | 1 | 162542042 | [T/C] |
| M1c163006813 | 1 | 163006813 | [A/G] |
| M1c163529703 | 1 | 163529703 | [T/C] |
| M1c164318966 | 1 | 164318966 | [T/C] |
| M1c164369597 | 1 | 164369597 | [T/G] |
| M1c164953049 | 1 | 164953049 | [T/C] |
| M1c165249364 | 1 | 165249364 | [A/G] |
| M1c165405945 | 1 | 165405945 | [A/C] |
| M1c166667484 | 1 | 166667484 | [T/C] |
| M1c166922802 | 1 | 166922802 | [T/C] |
| M1c167208869 | 1 | 167208869 | [T/C] |
| M1c167517455 | 1 | 167517455 | [A/C] |
| M1c167898531 | 1 | 167898531 | [T/C] |
| M1c168415203 | 1 | 168415203 | [T/C] |
| M1c168781384 | 1 | 168781384 | [A/G] |
| M1c168880111 | 1 | 168880111 | [A/G] |
| M1c169433544 | 1 | 169433544 | [A/G] |
| M1c169738761 | 1 | 169738761 | [A/C] |
| M1c169987768 | 1 | 169987768 | [T/C] |
| M1c170303746 | 1 | 170303746 | [A/G] |
| M1c170961674 | 1 | 170961674 | [A/C] |
| M1c171176156 | 1 | 171176156 | [T/C] |
| M1c171534977 | 1 | 171534977 | [A/G] |
| M1c171781205 | 1 | 171781205 | [T/C] |
| M1c172283355 | 1 | 172283355 | [A/G] |
| M1c172822044 | 1 | 172822044 | [A/G] |
| M1c173216477 | 1 | 173216477 | [A/G] |
| M1c173313597 | 1 | 173313597 | [T/C] |
| M1c173658873 | 1 | 173658873 | [A/C] |
| M1c174217484 | 1 | 174217484 | [T/C] |
| M1c174799835 | 1 | 174799835 | [A/C] |
| M1c175296774 | 1 | 175296774 | [A/G] |
| M1c175644273 | 1 | 175644273 | [T/G] |
| M1c175899522 | 1 | 175899522 | [A/G] |
| M1c176631516 | 1 | 176631516 | [T/G] |
| M1c176756849 | 1 | 176756849 | [T/C] |
| M1c177051403 | 1 | 177051403 | [A/C] |
| M1c177685787 | 1 | 177685787 | [T/C] |
| M1c178321579 | 1 | 178321579 | [T/C] |
| M1c178620294 | 1 | 178620294 | [A/G] |
| M1c178787864 | 1 | 178787864 | [T/G] |
| M1c179183309 | 1 | 179183309 | [T/C] |
| M1c179723333 | 1 | 179723333 | [A/C] |
| M1c180017078 | 1 | 180017078 | [A/G] |
| M1c180401222 | 1 | 180401222 | [T/C] |
| M1c180612182 | 1 | 180612182 | [A/G] |
| M1c180815772 | 1 | 180815772 | [T/C] |
| M1c181496189 | 1 | 181496189 | [A/G] |
| M1c181642361 | 1 | 181642361 | [A/C] |
| M1c181886175 | 1 | 181886175 | [T/C] |
| M1c182286085 | 1 | 182286085 | [T/C] |
| M1c182624145 | 1 | 182624145 | [A/C] |
| M1c182903204 | 1 | 182903204 | [A/G] |
| M1c183301837 | 1 | 183301837 | [A/C] |
| M1c183810049 | 1 | 183810049 | [T/G] |
| M1c184012303 | 1 | 184012303 | [T/C] |
| M1c184835375 | 1 | 184835375 | [T/C] |
| M1c186544429 | 1 | 186544429 | [T/C] |
| M1c186655481 | 1 | 186655481 | [A/C] |
| M1c187637214 | 1 | 187637214 | [T/C] |
| M1c187974308 | 1 | 187974308 | [T/G] |
| M1c188026235 | 1 | 188026235 | [A/C] |
| M1c188632028 | 1 | 188632028 | [T/C] |
| M1c188929503 | 1 | 188929503 | [T/C] |
| M1c189088166 | 1 | 189088166 | [A/G] |
| M1c189404618 | 1 | 189404618 | [A/G] |
| M1c189773451 | 1 | 189773451 | [A/C] |
| M1c190396353 | 1 | 190396353 | [T/C] |
| M1c190757670 | 1 | 190757670 | [A/G] |
| M1c191199165 | 1 | 191199165 | [T/C] |
| M1c191858782 | 1 | 191858782 | [A/G] |
| M1c192404883 | 1 | 192404883 | [T/C] |
| M1c192730873 | 1 | 192730873 | [T/C] |
| M1c193040565 | 1 | 193040565 | [T/G] |
| M1c193318034 | 1 | 193318034 | [A/G] |
| M1c193520375 | 1 | 193520375 | [A/G] |
| M1c193866498 | 1 | 193866498 | [A/G] |
| M1c194492514 | 1 | 194492514 | [T/G] |
| M1c195151360 | 1 | 195151360 | [T/C] |
| M1c195490960 | 1 | 195490960 | [T/C] |
| M1c195770152 | 1 | 195770152 | [T/G] |
| M1c196129249 | 1 | 196129249 | [A/G] |
| M1c196440524 | 1 | 196440524 | [A/G] |
| M1c196638863 | 1 | 196638863 | [T/G] |
| M1c197059330 | 1 | 197059330 | [T/G] |
| M1c197392077 | 1 | 197392077 | [A/G] |
| M1c197737925 | 1 | 197737925 | [A/G] |
| M1c198232289 | 1 | 198232289 | [A/C] |
| M1c198393224 | 1 | 198393224 | [A/C] |
| M1c199106407 | 1 | 199106407 | [T/G] |
| M1c199616162 | 1 | 199616162 | [A/G] |
| M1c199754459 | 1 | 199754459 | [T/G] |
| M1c200283416 | 1 | 200283416 | [T/C] |
| M1c200620278 | 1 | 200620278 | [T/G] |
| M1c200863989 | 1 | 200863989 | [A/G] |
| M1c201194199 | 1 | 201194199 | [A/G] |
| M1c202624996 | 1 | 202624996 | [T/C] |
| M1c202996012 | 1 | 202996012 | [A/G] |
| M1c203172991 | 1 | 203172991 | [T/C] |
| M1c203588391 | 1 | 203588391 | [A/G] |
| M1c204064632 | 1 | 204064632 | [A/G] |
| M1c204262817 | 1 | 204262817 | [T/C] |
| M1c204683219 | 1 | 204683219 | [T/C] |
| M1c205100034 | 1 | 205100034 | [A/G] |
| M1c205218834 | 1 | 205218834 | [A/G] |
| M1c205574384 | 1 | 205574384 | [T/C] |
| M1c206198374 | 1 | 206198374 | [A/G] |
| M1c206505952 | 1 | 206505952 | [A/G] |
| M1c206741997 | 1 | 206741997 | [T/G] |
| M1c207134068 | 1 | 207134068 | [T/C] |
| M1c207513822 | 1 | 207513822 | [A/G] |
| M1c207611620 | 1 | 207611620 | [T/G] |
| M1c208164897 | 1 | 208164897 | [T/C] |
| M1c208575411 | 1 | 208575411 | [T/C] |
| M1c208822485 | 1 | 208822485 | [T/G] |
| M1c209181321 | 1 | 209181321 | [T/G] |
| M1c209536591 | 1 | 209536591 | [A/C] |
| M1c209790211 | 1 | 209790211 | [A/C] |
| M1c210464167 | 1 | 210464167 | [T/C] |
| M1c210789591 | 1 | 210789591 | [A/G] |
| M1c211204733 | 1 | 211204733 | [A/G] |
| M1c211624360 | 1 | 211624360 | [T/G] |
| M1c212067307 | 1 | 212067307 | [A/G] |
| M1c212981433 | 1 | 212981433 | [T/C] |
| M1c213093477 | 1 | 213093477 | [A/C] |
| M1c213657132 | 1 | 213657132 | [T/C] |
| M1c213917676 | 1 | 213917676 | [T/G] |
| M1c214155334 | 1 | 214155334 | [A/G] |
| M1c215204046 | 1 | 215204046 | [A/G] |
| M1c215610443 | 1 | 215610443 | [T/C] |
| M1c216473790 | 1 | 216473790 | [T/C] |
| M1c216695475 | 1 | 216695475 | [A/G] |
| M1c216876926 | 1 | 216876926 | [A/G] |
| M1c217211476 | 1 | 217211476 | [T/C] |
| M1c217586463 | 1 | 217586463 | [T/C] |
| M1c217897948 | 1 | 217897948 | [T/C] |
| M1c218240365 | 1 | 218240365 | [A/G] |
| M1c218669150 | 1 | 218669150 | [A/G] |
| M1c219066760 | 1 | 219066760 | [T/C] |
| M1c219595391 | 1 | 219595391 | [A/C] |
| M1c220202522 | 1 | 220202522 | [A/C] |
| M1c220714804 | 1 | 220714804 | [T/C] |
| M1c221301796 | 1 | 221301796 | [T/G] |
| M1c221741596 | 1 | 221741596 | [A/G] |
| M1c222134417 | 1 | 222134417 | [T/C] |
| M1c222534306 | 1 | 222534306 | [A/G] |
| M1c222721099 | 1 | 222721099 | [T/C] |
| M1c223307476 | 1 | 223307476 | [A/G] |
| M1c223686008 | 1 | 223686008 | [T/C] |
| M1c223970764 | 1 | 223970764 | [A/C] |
| M1c224253721 | 1 | 224253721 | [T/G] |
| M1c224418808 | 1 | 224418808 | [A/G] |
| M1c224783681 | 1 | 224783681 | [T/C] |
| M1c225107542 | 1 | 225107542 | [A/G] |
| M1c225576680 | 1 | 225576680 | [T/C] |
| M1c225869057 | 1 | 225869057 | [A/G] |
| M1c226179348 | 1 | 226179348 | [A/G] |
| M1c226551713 | 1 | 226551713 | [A/C] |
| M1c226926690 | 1 | 226926690 | [A/C] |
| M1c227367207 | 1 | 227367207 | [T/C] |
| M1c227747807 | 1 | 227747807 | [A/G] |
| M1c228006619 | 1 | 228006619 | [T/C] |
| M1c228563313 | 1 | 228563313 | [A/C] |
| M1c228973956 | 1 | 228973956 | [T/C] |
| M1c229813944 | 1 | 229813944 | [T/C] |
| M1c230199260 | 1 | 230199260 | [T/C] |
| M1c230536683 | 1 | 230536683 | [A/G] |
| M1c231277741 | 1 | 231277741 | [T/G] |
| M1c231679748 | 1 | 231679748 | [T/C] |
| M1c232518928 | 1 | 232518928 | [T/G] |
| M1c232651456 | 1 | 232651456 | [T/C] |
| M1c233298798 | 1 | 233298798 | [A/C] |
| M1c233445932 | 1 | 233445932 | [T/G] |
| M1c233718957 | 1 | 233718957 | [T/G] |
| M1c234220809 | 1 | 234220809 | [A/G] |
| M1c234910750 | 1 | 234910750 | [T/G] |
| M1c235099748 | 1 | 235099748 | [T/C] |
| M1c235650077 | 1 | 235650077 | [T/C] |
| M1c235719155 | 1 | 235719155 | [T/G] |
| M1c236232147 | 1 | 236232147 | [T/C] |
| M1c236572983 | 1 | 236572983 | [A/G] |
| M1c236952975 | 1 | 236952975 | [A/C] |
| M1c237090833 | 1 | 237090833 | [A/G] |
| M1c237745337 | 1 | 237745337 | [A/G] |
| M1c237966670 | 1 | 237966670 | [T/C] |
| M1c238344080 | 1 | 238344080 | [T/G] |
| M1c238344479 | 1 | 238344479 | [T/C] |
| M1c238608286 | 1 | 238608286 | [T/C] |
| M1c239104443 | 1 | 239104443 | [A/G] |
| M1c239211167 | 1 | 239211167 | [A/G] |
| M1c239616366 | 1 | 239616366 | [A/G] |
| M1c239953811 | 1 | 239953811 | [A/G] |
| M1c240383709 | 1 | 240383709 | [A/G] |
| M1c240716730 | 1 | 240716730 | [T/C] |
| M1c240963186 | 1 | 240963186 | [T/C] |
| M1c241371465 | 1 | 241371465 | [T/C] |
| M1c242434885 | 1 | 242434885 | [T/C] |
| M1c242683374 | 1 | 242683374 | [A/G] |
| M1c243439838 | 1 | 243439838 | [T/C] |
| M1c243813136 | 1 | 243813136 | [T/C] |
| M1c244210033 | 1 | 244210033 | [T/C] |
| M1c244512305 | 1 | 244512305 | [T/G] |
| M1c244771107 | 1 | 244771107 | [A/G] |
| M1c245312038 | 1 | 245312038 | [T/G] |
| M1c245479447 | 1 | 245479447 | [A/G] |
| M1c245694133 | 1 | 245694133 | [A/G] |
| M1c246137235 | 1 | 246137235 | [T/C] |
| M1c246684950 | 1 | 246684950 | [T/G] |
| M1c246815190 | 1 | 246815190 | [T/C] |
| M1c247173464 | 1 | 247173464 | [T/C] |
| M1c247419009 | 1 | 247419009 | [A/G] |
| M1c248750685 | 1 | 248750685 | [A/G] |
| M1c249414753 | 1 | 249414753 | [T/C] |
| M1c249538343 | 1 | 249538343 | [A/G] |
| M1c250093789 | 1 | 250093789 | [T/C] |
| M1c250188959 | 1 | 250188959 | [A/G] |
| M1c250558028 | 1 | 250558028 | [A/G] |
| M1c251386866 | 1 | 251386866 | [T/C] |
| M1c252322358 | 1 | 252322358 | [T/G] |
| M1c252794761 | 1 | 252794761 | [A/C] |
| M1c253019475 | 1 | 253019475 | [A/C] |
| M1c253393521 | 1 | 253393521 | [T/C] |
| M1c253720517 | 1 | 253720517 | [A/G] |
| M1c253906844 | 1 | 253906844 | [A/G] |
| M1c254560116 | 1 | 254560116 | [A/G] |
| M1c254800516 | 1 | 254800516 | [A/G] |
| M1c255318743 | 1 | 255318743 | [T/C] |
| M1c255703366 | 1 | 255703366 | [T/G] |
| M1c256292686 | 1 | 256292686 | [T/G] |
| M1c256296924 | 1 | 256296924 | [T/C] |
| M1c256660716 | 1 | 256660716 | [T/C] |
| M1c257186929 | 1 | 257186929 | [A/G] |
| M1c257808050 | 1 | 257808050 | [A/G] |
| M1c258440066 | 1 | 258440066 | [A/G] |
| M1c259012638 | 1 | 259012638 | [A/G] |
| M1c259215113 | 1 | 259215113 | [T/C] |
| M1c259382325 | 1 | 259382325 | [A/G] |
| M1c259930420 | 1 | 259930420 | [T/C] |
| M1c260134702 | 1 | 260134702 | [A/C] |
| M1c260518996 | 1 | 260518996 | [T/C] |
| M1c260957600 | 1 | 260957600 | [T/C] |
| M1c261666221 | 1 | 261666221 | [T/C] |
| M1c262326428 | 1 | 262326428 | [A/G] |
| M1c263129533 | 1 | 263129533 | [T/C] |
| M1c263195388 | 1 | 263195388 | [A/G] |
| M1c263652293 | 1 | 263652293 | [T/C] |
| M1c264084699 | 1 | 264084699 | [T/C] |
| M1c264203806 | 1 | 264203806 | [T/C] |
| M1c264528611 | 1 | 264528611 | [A/C] |
| M1c264957465 | 1 | 264957465 | [A/C] |
| M1c265317625 | 1 | 265317625 | [A/G] |
| M1c265652759 | 1 | 265652759 | [A/G] |
| M1c266369422 | 1 | 266369422 | [A/G] |
| M1c266910474 | 1 | 266910474 | [A/G] |
| M1c266951673 | 1 | 266951673 | [T/G] |
| M1c267532399 | 1 | 267532399 | [A/G] |
| M1c267783894 | 1 | 267783894 | [A/G] |
| M1c267908448 | 1 | 267908448 | [A/C] |
| M1c268561317 | 1 | 268561317 | [T/C] |
| M1c269109334 | 1 | 269109334 | [T/C] |
| M1c269354199 | 1 | 269354199 | [T/C] |
| M1c269936927 | 1 | 269936927 | [T/G] |
| M1c270134887 | 1 | 270134887 | [T/C] |
| M1c270661719 | 1 | 270661719 | [T/C] |
| M1c271027973 | 1 | 271027973 | [T/C] |
| M1c271321424 | 1 | 271321424 | [A/G] |
| M1c271627282 | 1 | 271627282 | [A/G] |
| M1c271768436 | 1 | 271768436 | [T/G] |
| M1c272219577 | 1 | 272219577 | [T/C] |
| M1c272572756 | 1 | 272572756 | [A/G] |
| M1c272801908 | 1 | 272801908 | [T/G] |
| M1c273372443 | 1 | 273372443 | [T/C] |
| M1c273693583 | 1 | 273693583 | [T/C] |
| M1c273983998 | 1 | 273983998 | [T/C] |
| M1c274408805 | 1 | 274408805 | [A/G] |
| M1c274710543 | 1 | 274710543 | [T/C] |
| M1c275031687 | 1 | 275031687 | [T/C] |
| M1c275682256 | 1 | 275682256 | [T/C] |
| M1c275866874 | 1 | 275866874 | [T/G] |
| M1c276417408 | 1 | 276417408 | [T/C] |
| M1c276538996 | 1 | 276538996 | [A/G] |
| M1c277142198 | 1 | 277142198 | [T/C] |
| M1c277375688 | 1 | 277375688 | [T/G] |
| M1c277602268 | 1 | 277602268 | [T/C] |
| M1c278131888 | 1 | 278131888 | [A/G] |
| M1c278356674 | 1 | 278356674 | [A/G] |
| M1c278908338 | 1 | 278908338 | [T/G] |
| M1c279120251 | 1 | 279120251 | [A/G] |
| M1c279414826 | 1 | 279414826 | [T/C] |
| M1c279875777 | 1 | 279875777 | [T/C] |
| M1c280219015 | 1 | 280219015 | [A/C] |
| M1c280581138 | 1 | 280581138 | [T/G] |
| M1c280975319 | 1 | 280975319 | [A/G] |
| M1c281590394 | 1 | 281590394 | [T/C] |
| M1c281709453 | 1 | 281709453 | [A/G] |
| M1c282531182 | 1 | 282531182 | [T/G] |
| M1c282894969 | 1 | 282894969 | [A/G] |
| M1c283601031 | 1 | 283601031 | [T/C] |
| M1c283862393 | 1 | 283862393 | [A/G] |
| M1c284215734 | 1 | 284215734 | [A/G] |
| M1c285068909 | 1 | 285068909 | [A/C] |
| M1c285229087 | 1 | 285229087 | [T/C] |
| M1c285635826 | 1 | 285635826 | [T/G] |
| M1c285810886 | 1 | 285810886 | [T/C] |
| M1c286403940 | 1 | 286403940 | [A/C] |
| M1c286660232 | 1 | 286660232 | [A/G] |
| M1c286858844 | 1 | 286858844 | [A/G] |
| M1c287474492 | 1 | 287474492 | [A/C] |
| M1c287492708 | 1 | 287492708 | [T/G] |
| M1c287595435 | 1 | 287595435 | [T/C] |
| M1c288170692 | 1 | 288170692 | [T/C] |
| M1c288261686 | 1 | 288261686 | [A/C] |
| M1c288864593 | 1 | 288864593 | [T/C] |
| M1c289060331 | 1 | 289060331 | [T/G] |
| M1c289219708 | 1 | 289219708 | [A/G] |
| M1c289546922 | 1 | 289546922 | [T/C] |
| M1c289688925 | 1 | 289688925 | [T/C] |
| M1c289852748 | 1 | 289852748 | [T/G] |
| M1c290012101 | 1 | 290012101 | [A/C] |
| M1c290137067 | 1 | 290137067 | [A/G] |
| M1c290306207 | 1 | 290306207 | [A/G] |
| M1c290932181 | 1 | 290932181 | [T/C] |
| M1c291254645 | 1 | 291254645 | [A/G] |
| M1c291311456 | 1 | 291311456 | [A/G] |
| M1c291926607 | 1 | 291926607 | [A/G] |
| M1c292278876 | 1 | 292278876 | [A/G] |
| M1c292404115 | 1 | 292404115 | [A/G] |
| M1c292674605 | 1 | 292674605 | [T/C] |
| M1c293576645 | 1 | 293576645 | [T/C] |
| M1c293998654 | 1 | 293998654 | [T/C] |
| M1c294143953 | 1 | 294143953 | [A/C] |
| M1c294659144 | 1 | 294659144 | [T/C] |
| M1c295018337 | 1 | 295018337 | [T/C] |
| M1c295062219 | 1 | 295062219 | [A/C] |
| M1c295310386 | 1 | 295310386 | [A/G] |
| M1c295480548 | 1 | 295480548 | [T/G] |
| M1c295535709 | 1 | 295535709 | [A/G] |
| M1c295763056 | 1 | 295763056 | [A/G] |
| M1c295879905 | 1 | 295879905 | [A/C] |
| M1c296281664 | 1 | 296281664 | [A/G] |
| M1c296767385 | 1 | 296767385 | [T/C] |
| M1c296949173 | 1 | 296949173 | [A/G] |
| M1c297374055 | 1 | 297374055 | [A/G] |
| M1c297916197 | 1 | 297916197 | [A/C] |
| M1c298331289 | 1 | 298331289 | [A/C] |
| M1c298786085 | 1 | 298786085 | [T/C] |
| M1c298879872 | 1 | 298879872 | [A/G] |
| M1c299287996 | 1 | 299287996 | [T/G] |
| M1c299609421 | 1 | 299609421 | [A/G] |
| M1c300871741 | 1 | 300871741 | [A/G] |
| M1c300993479 | 1 | 300993479 | [A/G] |
| M2c89546 | 2 | 89546 | [T/C] |
| M2c508913 | 2 | 508913 | [A/G] |
| M2c897617 | 2 | 897617 | [A/G] |
| M2c1037526 | 2 | 1037526 | [T/C] |
| M2c1138614 | 2 | 1138614 | [T/C] |
| M2c1352209 | 2 | 1352209 | [T/C] |
| M2c1459796 | 2 | 1459796 | [A/G] |
| M2c1461969 | 2 | 1461969 | [A/C] |
| M2c1777005 | 2 | 1777005 | [T/G] |
| M2c1814888 | 2 | 1814888 | [T/C] |
| M2c2107971 | 2 | 2107971 | [A/G] |
| M2c2539484 | 2 | 2539484 | [T/G] |
| M2c2821261 | 2 | 2821261 | [A/G] |
| M2c2912448 | 2 | 2912448 | [T/C] |
| M2c3104095 | 2 | 3104095 | [A/G] |
| M2c3226523 | 2 | 3226523 | [A/G] |
| M2c3614453 | 2 | 3614453 | [A/G] |
| M2c3614454 | 2 | 3614454 | [T/C] |
| M2c3860095 | 2 | 3860095 | [T/G] |
| M2c4299802 | 2 | 4299802 | [T/C] |
| M2c4584301 | 2 | 4584301 | [A/G] |
| M2c5229368 | 2 | 5229368 | [A/G] |
| M2c5571006 | 2 | 5571006 | [T/G] |
| M2c5863401 | 2 | 5863401 | [T/C] |
| M2c6020521 | 2 | 6020521 | [T/G] |
| M2c6177366 | 2 | 6177366 | [A/C] |
| M2c6467240 | 2 | 6467240 | [T/G] |
| M2c7002705 | 2 | 7002705 | [T/C] |
| M2c7314097 | 2 | 7314097 | [A/C] |
| M2c7623325 | 2 | 7623325 | [A/C] |
| M2c7843880 | 2 | 7843880 | [A/C] |
| M2c8294827 | 2 | 8294827 | [T/C] |
| M2c8803658 | 2 | 8803658 | [T/C] |
| M2c8908971 | 2 | 8908971 | [T/C] |
| M2c9267276 | 2 | 9267276 | [A/G] |
| M2c9553114 | 2 | 9553114 | [A/G] |
| M2c9968312 | 2 | 9968312 | [A/C] |
| M2c10479329 | 2 | 10479329 | [T/C] |
| M2c10570548 | 2 | 10570548 | [A/C] |
| M2c11202954 | 2 | 11202954 | [A/G] |
| M2c11366541 | 2 | 11366541 | [A/C] |
| M2c11590084 | 2 | 11590084 | [A/G] |
| M2c12105303 | 2 | 12105303 | [A/G] |
| M2c12266108 | 2 | 12266108 | [T/G] |
| M2c12677861 | 2 | 12677861 | [T/G] |
| M2c13154161 | 2 | 13154161 | [A/G] |
| M2c13404342 | 2 | 13404342 | [T/C] |
| M2c13783137 | 2 | 13783137 | [A/G] |
| M2c14267707 | 2 | 14267707 | [A/G] |
| M2c14536595 | 2 | 14536595 | [A/C] |
| M2c14715778 | 2 | 14715778 | [A/C] |
| M2c15273214 | 2 | 15273214 | [T/C] |
| M2c15571675 | 2 | 15571675 | [T/C] |
| M2c15987847 | 2 | 15987847 | [T/G] |
| M2c16122130 | 2 | 16122130 | [A/C] |
| M2c16469945 | 2 | 16469945 | [A/G] |
| M2c16938729 | 2 | 16938729 | [A/G] |
| M2c17348560 | 2 | 17348560 | [T/C] |
| M2c17607287 | 2 | 17607287 | [T/C] |
| M2c18002187 | 2 | 18002187 | [A/G] |
| M2c18203717 | 2 | 18203717 | [A/G] |
| M2c18513155 | 2 | 18513155 | [T/G] |
| M2c18814972 | 2 | 18814972 | [A/G] |
| M2c19197584 | 2 | 19197584 | [T/C] |
| M2c20205515 | 2 | 20205515 | [T/C] |
| M2c20532463 | 2 | 20532463 | [A/C] |
| M2c20857105 | 2 | 20857105 | [T/G] |
| M2c21268615 | 2 | 21268615 | [A/C] |
| M2c21638365 | 2 | 21638365 | [A/G] |
| M2c22105448 | 2 | 22105448 | [A/C] |
| M2c22612511 | 2 | 22612511 | [A/G] |
| M2c22995036 | 2 | 22995036 | [A/G] |
| M2c23542718 | 2 | 23542718 | [T/C] |
| M2c23706838 | 2 | 23706838 | [A/C] |
| M2c23980808 | 2 | 23980808 | [A/G] |
| M2c24333805 | 2 | 24333805 | [T/C] |
| M2c24834904 | 2 | 24834904 | [A/G] |
| M2c24969423 | 2 | 24969423 | [T/C] |
| M2c25614809 | 2 | 25614809 | [T/C] |
| M2c25743217 | 2 | 25743217 | [A/G] |
| M2c26383735 | 2 | 26383735 | [T/C] |
| M2c26727491 | 2 | 26727491 | [A/G] |
| M2c27052498 | 2 | 27052498 | [A/C] |
| M2c27562776 | 2 | 27562776 | [A/C] |
| M2c27872051 | 2 | 27872051 | [A/C] |
| M2c28036817 | 2 | 28036817 | [T/C] |
| M2c28401733 | 2 | 28401733 | [T/G] |
| M2c28722531 | 2 | 28722531 | [T/C] |
| M2c29269601 | 2 | 29269601 | [A/G] |
| M2c29633855 | 2 | 29633855 | [T/C] |
| M2c29943907 | 2 | 29943907 | [T/C] |
| M2c30360747 | 2 | 30360747 | [A/G] |
| M2c30587345 | 2 | 30587345 | [A/G] |
| M2c31046930 | 2 | 31046930 | [A/G] |
| M2c31161128 | 2 | 31161128 | [A/G] |
| M2c31924656 | 2 | 31924656 | [A/G] |
| M2c32334160 | 2 | 32334160 | [T/C] |
| M2c32492387 | 2 | 32492387 | [A/G] |
| M2c33499030 | 2 | 33499030 | [A/G] |
| M2c33767405 | 2 | 33767405 | [T/C] |
| M2c33954342 | 2 | 33954342 | [T/C] |
| M2c34343317 | 2 | 34343317 | [T/C] |
| M2c34688285 | 2 | 34688285 | [T/C] |
| M2c35035102 | 2 | 35035102 | [T/C] |
| M2c35326763 | 2 | 35326763 | [T/G] |
| M2c35880754 | 2 | 35880754 | [T/C] |
| M2c36586767 | 2 | 36586767 | [T/C] |
| M2c36836463 | 2 | 36836463 | [A/G] |
| M2c37198459 | 2 | 37198459 | [A/G] |
| M2c37557891 | 2 | 37557891 | [T/G] |
| M2c37904294 | 2 | 37904294 | [T/G] |
| M2c38134920 | 2 | 38134920 | [T/C] |
| M2c38560991 | 2 | 38560991 | [A/G] |
| M2c38988846 | 2 | 38988846 | [T/C] |
| M2c39046734 | 2 | 39046734 | [A/G] |
| M2c39615056 | 2 | 39615056 | [A/G] |
| M2c39796017 | 2 | 39796017 | [A/C] |
| M2c40350502 | 2 | 40350502 | [T/C] |
| M2c40684095 | 2 | 40684095 | [T/C] |
| M2c41030315 | 2 | 41030315 | [T/C] |
| M2c41305922 | 2 | 41305922 | [T/C] |
| M2c41497231 | 2 | 41497231 | [T/C] |
| M2c41898960 | 2 | 41898960 | [T/C] |
| M2c42357464 | 2 | 42357464 | [A/G] |
| M2c42677020 | 2 | 42677020 | [A/G] |
| M2c42804292 | 2 | 42804292 | [A/C] |
| M2c43436017 | 2 | 43436017 | [T/C] |
| M2c43753307 | 2 | 43753307 | [A/G] |
| M2c44014360 | 2 | 44014360 | [A/C] |
| M2c44474179 | 2 | 44474179 | [T/C] |
| M2c44796274 | 2 | 44796274 | [T/C] |
| M2c44944236 | 2 | 44944236 | [A/G] |
| M2c45194157 | 2 | 45194157 | [T/C] |
| M2c46437351 | 2 | 46437351 | [A/G] |
| M2c46604852 | 2 | 46604852 | [T/G] |
| M2c47557853 | 2 | 47557853 | [A/G] |
| M2c47852661 | 2 | 47852661 | [T/C] |
| M2c48108852 | 2 | 48108852 | [T/C] |
| M2c48592119 | 2 | 48592119 | [T/C] |
| M2c48658743 | 2 | 48658743 | [A/G] |
| M2c49056860 | 2 | 49056860 | [A/C] |
| M2c49302180 | 2 | 49302180 | [T/C] |
| M2c49922714 | 2 | 49922714 | [A/G] |
| M2c49981711 | 2 | 49981711 | [T/C] |
| M2c50643209 | 2 | 50643209 | [A/G] |
| M2c50809256 | 2 | 50809256 | [T/C] |
| M2c51239251 | 2 | 51239251 | [T/C] |
| M2c51443677 | 2 | 51443677 | [A/G] |
| M2c51753330 | 2 | 51753330 | [A/G] |
| M2c52318756 | 2 | 52318756 | [A/C] |
| M2c53366340 | 2 | 53366340 | [A/G] |
| M2c53680048 | 2 | 53680048 | [A/G] |
| M2c53845268 | 2 | 53845268 | [T/C] |
| M2c54283670 | 2 | 54283670 | [A/G] |
| M2c54763040 | 2 | 54763040 | [A/G] |
| M2c54962529 | 2 | 54962529 | [A/G] |
| M2c55448426 | 2 | 55448426 | [T/C] |
| M2c55558966 | 2 | 55558966 | [A/G] |
| M2c55828786 | 2 | 55828786 | [T/C] |
| M2c56809223 | 2 | 56809223 | [T/C] |
| M2c57484588 | 2 | 57484588 | [A/G] |
| M2c57543341 | 2 | 57543341 | [T/C] |
| M2c58012703 | 2 | 58012703 | [A/G] |
| M2c58471987 | 2 | 58471987 | [A/G] |
| M2c58759622 | 2 | 58759622 | [A/G] |
| M2c59013161 | 2 | 59013161 | [T/C] |
| M2c59284227 | 2 | 59284227 | [A/G] |
| M2c59633256 | 2 | 59633256 | [A/G] |
| M2c60070537 | 2 | 60070537 | [T/C] |
| M2c60417118 | 2 | 60417118 | [T/C] |
| M2c60658908 | 2 | 60658908 | [A/G] |
| M2c61246491 | 2 | 61246491 | [A/G] |
| M2c61323852 | 2 | 61323852 | [T/C] |
| M2c61857265 | 2 | 61857265 | [A/G] |
| M2c62099671 | 2 | 62099671 | [A/G] |
| M2c62377431 | 2 | 62377431 | [A/G] |
| M2c62992444 | 2 | 62992444 | [T/C] |
| M2c63072647 | 2 | 63072647 | [T/C] |
| M2c63943440 | 2 | 63943440 | [A/C] |
| M2c64278250 | 2 | 64278250 | [T/C] |
| M2c64697810 | 2 | 64697810 | [T/C] |
| M2c64741491 | 2 | 64741491 | [T/G] |
| M2c65182714 | 2 | 65182714 | [A/G] |
| M2c66251114 | 2 | 66251114 | [T/C] |
| M2c67081780 | 2 | 67081780 | [A/G] |
| M2c67149723 | 2 | 67149723 | [T/C] |
| M2c67538839 | 2 | 67538839 | [T/G] |
| M2c68085705 | 2 | 68085705 | [T/C] |
| M2c68289218 | 2 | 68289218 | [A/C] |
| M2c68692542 | 2 | 68692542 | [T/C] |
| M2c69177375 | 2 | 69177375 | [A/G] |
| M2c69296335 | 2 | 69296335 | [A/G] |
| M2c69706693 | 2 | 69706693 | [T/C] |
| M2c69905740 | 2 | 69905740 | [T/C] |
| M2c71148193 | 2 | 71148193 | [T/C] |
| M2c71585816 | 2 | 71585816 | [T/C] |
| M2c71606538 | 2 | 71606538 | [A/C] |
| M2c72205748 | 2 | 72205748 | [T/C] |
| M2c72450780 | 2 | 72450780 | [T/C] |
| M2c72672047 | 2 | 72672047 | [A/G] |
| M2c73068180 | 2 | 73068180 | [A/G] |
| M2c73806369 | 2 | 73806369 | [T/C] |
| M2c74045919 | 2 | 74045919 | [A/G] |
| M2c74566844 | 2 | 74566844 | [T/G] |
| M2c75317392 | 2 | 75317392 | [T/C] |
| M2c75452892 | 2 | 75452892 | [A/G] |
| M2c76199507 | 2 | 76199507 | [T/C] |
| M2c76642295 | 2 | 76642295 | [A/G] |
| M2c76919758 | 2 | 76919758 | [A/C] |
| M2c77358754 | 2 | 77358754 | [A/C] |
| M2c77610195 | 2 | 77610195 | [T/C] |
| M2c77816678 | 2 | 77816678 | [T/C] |
| M2c78230618 | 2 | 78230618 | [T/G] |
| M2c78944743 | 2 | 78944743 | [A/G] |
| M2c79182340 | 2 | 79182340 | [A/G] |
| M2c79571040 | 2 | 79571040 | [A/C] |
| M2c80057475 | 2 | 80057475 | [A/G] |
| M2c80444962 | 2 | 80444962 | [A/G] |
| M2c80583306 | 2 | 80583306 | [A/G] |
| M2c81451695 | 2 | 81451695 | [T/C] |
| M2c81808233 | 2 | 81808233 | [T/C] |
| M2c81979724 | 2 | 81979724 | [T/C] |
| M2c82246611 | 2 | 82246611 | [A/G] |
| M2c82813119 | 2 | 82813119 | [A/G] |
| M2c83169259 | 2 | 83169259 | [T/C] |
| M2c83487387 | 2 | 83487387 | [A/G] |
| M2c83795388 | 2 | 83795388 | [A/G] |
| M2c84184272 | 2 | 84184272 | [T/C] |
| M2c84624989 | 2 | 84624989 | [A/C] |
| M2c84651378 | 2 | 84651378 | [T/C] |
| M2c85179990 | 2 | 85179990 | [T/G] |
| M2c85472407 | 2 | 85472407 | [T/C] |
| M2c86002997 | 2 | 86002997 | [A/G] |
| M2c86252468 | 2 | 86252468 | [A/C] |
| M2c86654199 | 2 | 86654199 | [A/C] |
| M2c86892221 | 2 | 86892221 | [A/G] |
| M2c87077679 | 2 | 87077679 | [T/C] |
| M2c87657469 | 2 | 87657469 | [A/G] |
| M2c87722186 | 2 | 87722186 | [T/C] |
| M2c88795333 | 2 | 88795333 | [A/G] |
| M2c89228669 | 2 | 89228669 | [A/C] |
| M2c89708857 | 2 | 89708857 | [T/C] |
| M2c89844216 | 2 | 89844216 | [A/G] |
| M2c90377525 | 2 | 90377525 | [T/C] |
| M2c90534099 | 2 | 90534099 | [T/C] |
| M2c90891171 | 2 | 90891171 | [A/G] |
| M2c91347086 | 2 | 91347086 | [T/C] |
| M2c92005784 | 2 | 92005784 | [T/C] |
| M2c92453663 | 2 | 92453663 | [T/C] |
| M2c92777341 | 2 | 92777341 | [A/G] |
| M2c92890787 | 2 | 92890787 | [T/C] |
| M2c93358541 | 2 | 93358541 | [A/G] |
| M2c93811988 | 2 | 93811988 | [A/G] |
| M2c94807444 | 2 | 94807444 | [A/C] |
| M2c95003014 | 2 | 95003014 | [T/C] |
| M2c95462824 | 2 | 95462824 | [T/G] |
| M2c95647999 | 2 | 95647999 | [A/C] |
| M2c96341714 | 2 | 96341714 | [A/G] |
| M2c97283113 | 2 | 97283113 | [A/G] |
| M2c97439944 | 2 | 97439944 | [T/C] |
| M2c98289252 | 2 | 98289252 | [A/G] |
| M2c98603283 | 2 | 98603283 | [A/G] |
| M2c98751475 | 2 | 98751475 | [A/G] |
| M2c99271356 | 2 | 99271356 | [T/C] |
| M2c99967756 | 2 | 99967756 | [A/G] |
| M2c100409949 | 2 | 100409949 | [A/C] |
| M2c100657888 | 2 | 100657888 | [A/C] |
| M2c100762388 | 2 | 100762388 | [A/G] |
| M2c101173240 | 2 | 101173240 | [A/G] |
| M2c101543532 | 2 | 101543532 | [A/G] |
| M2c101958254 | 2 | 101958254 | [A/G] |
| M2c102137512 | 2 | 102137512 | [A/G] |
| M2c102751133 | 2 | 102751133 | [A/G] |
| M2c102929302 | 2 | 102929302 | [T/G] |
| M2c103173290 | 2 | 103173290 | [T/G] |
| M2c103685661 | 2 | 103685661 | [A/G] |
| M2c104180496 | 2 | 104180496 | [T/C] |
| M2c104491417 | 2 | 104491417 | [T/C] |
| M2c104728729 | 2 | 104728729 | [T/C] |
| M2c105165841 | 2 | 105165841 | [T/C] |
| M2c105418278 | 2 | 105418278 | [T/C] |
| M2c105564522 | 2 | 105564522 | [A/C] |
| M2c106286991 | 2 | 106286991 | [A/G] |
| M2c107286362 | 2 | 107286362 | [T/C] |
| M2c107850629 | 2 | 107850629 | [A/G] |
| M2c108231013 | 2 | 108231013 | [T/C] |
| M2c108631711 | 2 | 108631711 | [A/G] |
| M2c108679496 | 2 | 108679496 | [A/C] |
| M2c108996680 | 2 | 108996680 | [A/G] |
| M2c109492167 | 2 | 109492167 | [A/G] |
| M2c109876726 | 2 | 109876726 | [A/G] |
| M2c110482628 | 2 | 110482628 | [A/C] |
| M2c110746025 | 2 | 110746025 | [A/G] |
| M2c111315661 | 2 | 111315661 | [A/G] |
| M2c111980486 | 2 | 111980486 | [A/G] |
| M2c112084186 | 2 | 112084186 | [T/G] |
| M2c112639588 | 2 | 112639588 | [A/C] |
| M2c112864202 | 2 | 112864202 | [T/C] |
| M2c113360468 | 2 | 113360468 | [A/G] |
| M2c113779015 | 2 | 113779015 | [A/G] |
| M2c114021801 | 2 | 114021801 | [T/C] |
| M2c114163887 | 2 | 114163887 | [T/C] |
| M2c115068576 | 2 | 115068576 | [T/C] |
| M2c115322179 | 2 | 115322179 | [A/G] |
| M2c115744696 | 2 | 115744696 | [T/C] |
| M2c116170981 | 2 | 116170981 | [T/C] |
| M2c116793998 | 2 | 116793998 | [A/G] |
| M2c117049133 | 2 | 117049133 | [T/C] |
| M2c117507424 | 2 | 117507424 | [A/G] |
| M2c117842831 | 2 | 117842831 | [T/C] |
| M2c117954877 | 2 | 117954877 | [T/G] |
| M2c118571489 | 2 | 118571489 | [T/C] |
| M2c118910380 | 2 | 118910380 | [T/C] |
| M2c119211586 | 2 | 119211586 | [A/G] |
| M2c119489989 | 2 | 119489989 | [A/G] |
| M2c119640474 | 2 | 119640474 | [A/C] |
| M2c120234020 | 2 | 120234020 | [A/G] |
| M2c120654810 | 2 | 120654810 | [A/G] |
| M2c120664750 | 2 | 120664750 | [A/G] |
| M2c121903133 | 2 | 121903133 | [T/C] |
| M2c122583901 | 2 | 122583901 | [T/C] |
| M2c122952551 | 2 | 122952551 | [T/C] |
| M2c123330491 | 2 | 123330491 | [T/C] |
| M2c123597881 | 2 | 123597881 | [A/G] |
| M2c123980396 | 2 | 123980396 | [A/C] |
| M2c124423209 | 2 | 124423209 | [A/G] |
| M2c125296247 | 2 | 125296247 | [A/C] |
| M2c125710821 | 2 | 125710821 | [A/G] |
| M2c125809599 | 2 | 125809599 | [T/C] |
| M2c126443043 | 2 | 126443043 | [A/C] |
| M2c126554048 | 2 | 126554048 | [T/C] |
| M2c127093416 | 2 | 127093416 | [A/G] |
| M2c127602566 | 2 | 127602566 | [A/G] |
| M2c128064092 | 2 | 128064092 | [A/G] |
| M2c128452607 | 2 | 128452607 | [T/G] |
| M2c128565957 | 2 | 128565957 | [A/G] |
| M2c129121514 | 2 | 129121514 | [A/C] |
| M2c129306377 | 2 | 129306377 | [A/G] |
| M2c129786984 | 2 | 129786984 | [T/C] |
| M2c130063733 | 2 | 130063733 | [T/C] |
| M2c130524875 | 2 | 130524875 | [T/C] |
| M2c130744434 | 2 | 130744434 | [T/G] |
| M2c130953742 | 2 | 130953742 | [A/G] |
| M2c131349382 | 2 | 131349382 | [T/C] |
| M2c131679950 | 2 | 131679950 | [T/C] |
| M2c132572254 | 2 | 132572254 | [A/G] |
| M2c133268338 | 2 | 133268338 | [A/G] |
| M2c133701239 | 2 | 133701239 | [T/C] |
| M2c134442168 | 2 | 134442168 | [A/G] |
| M2c134968782 | 2 | 134968782 | [A/G] |
| M2c135394828 | 2 | 135394828 | [T/C] |
| M2c135723631 | 2 | 135723631 | [A/G] |
| M2c135956760 | 2 | 135956760 | [A/G] |
| M2c136182428 | 2 | 136182428 | [A/C] |
| M2c136666707 | 2 | 136666707 | [A/G] |
| M2c136841905 | 2 | 136841905 | [A/G] |
| M2c137261832 | 2 | 137261832 | [A/G] |
| M2c137773539 | 2 | 137773539 | [T/C] |
| M2c137985405 | 2 | 137985405 | [T/G] |
| M2c138254414 | 2 | 138254414 | [T/C] |
| M2c138562283 | 2 | 138562283 | [A/G] |
| M2c139244009 | 2 | 139244009 | [A/G] |
| M2c139645636 | 2 | 139645636 | [T/C] |
| M2c140323417 | 2 | 140323417 | [T/C] |
| M2c140659872 | 2 | 140659872 | [T/C] |
| M2c140902041 | 2 | 140902041 | [T/C] |
| M2c141363433 | 2 | 141363433 | [T/G] |
| M2c142029070 | 2 | 142029070 | [A/G] |
| M2c142355751 | 2 | 142355751 | [A/G] |
| M2c142864628 | 2 | 142864628 | [A/G] |
| M2c143258975 | 2 | 143258975 | [T/C] |
| M2c143671873 | 2 | 143671873 | [T/C] |
| M2c144046558 | 2 | 144046558 | [A/C] |
| M2c144432572 | 2 | 144432572 | [A/G] |
| M2c144987171 | 2 | 144987171 | [T/G] |
| M2c145351866 | 2 | 145351866 | [T/C] |
| M2c145422086 | 2 | 145422086 | [A/G] |
| M2c146015613 | 2 | 146015613 | [A/C] |
| M2c146123651 | 2 | 146123651 | [T/C] |
| M2c146410262 | 2 | 146410262 | [A/G] |
| M2c147038935 | 2 | 147038935 | [A/C] |
| M2c147310022 | 2 | 147310022 | [T/C] |
| M2c147618315 | 2 | 147618315 | [T/C] |
| M2c148145818 | 2 | 148145818 | [T/C] |
| M2c148658795 | 2 | 148658795 | [T/G] |
| M2c148876307 | 2 | 148876307 | [T/G] |
| M2c149340387 | 2 | 149340387 | [A/G] |
| M2c149518582 | 2 | 149518582 | [T/C] |
| M2c150143559 | 2 | 150143559 | [A/G] |
| M2c150804168 | 2 | 150804168 | [A/G] |
| M2c151077381 | 2 | 151077381 | [T/C] |
| M2c151261763 | 2 | 151261763 | [T/C] |
| M2c151606158 | 2 | 151606158 | [A/G] |
| M2c152236474 | 2 | 152236474 | [A/G] |
| M2c153150743 | 2 | 153150743 | [A/C] |
| M2c153437688 | 2 | 153437688 | [T/C] |
| M2c153794262 | 2 | 153794262 | [A/G] |
| M2c154166312 | 2 | 154166312 | [A/G] |
| M2c154793647 | 2 | 154793647 | [T/C] |
| M2c155086581 | 2 | 155086581 | [T/C] |
| M2c155631681 | 2 | 155631681 | [T/C] |
| M2c155851853 | 2 | 155851853 | [T/G] |
| M2c156181486 | 2 | 156181486 | [A/G] |
| M2c156399388 | 2 | 156399388 | [T/C] |
| M2c156895812 | 2 | 156895812 | [A/C] |
| M2c157209974 | 2 | 157209974 | [A/G] |
| M2c157477179 | 2 | 157477179 | [A/G] |
| M2c157899241 | 2 | 157899241 | [T/C] |
| M2c158388088 | 2 | 158388088 | [A/G] |
| M2c158613327 | 2 | 158613327 | [T/C] |
| M2c159202972 | 2 | 159202972 | [T/G] |
| M2c159584126 | 2 | 159584126 | [A/C] |
| M2c159791109 | 2 | 159791109 | [T/C] |
| M2c160250729 | 2 | 160250729 | [T/C] |
| M2c160783178 | 2 | 160783178 | [T/C] |
| M2c161049112 | 2 | 161049112 | [A/G] |
| M2c161514798 | 2 | 161514798 | [A/G] |
| M2c161872039 | 2 | 161872039 | [A/C] |
| M2c162435400 | 2 | 162435400 | [A/G] |
| M2c162568986 | 2 | 162568986 | [A/G] |
| M2c162962496 | 2 | 162962496 | [A/G] |
| M2c163452824 | 2 | 163452824 | [A/C] |
| M2c163883088 | 2 | 163883088 | [A/G] |
| M2c164191885 | 2 | 164191885 | [T/C] |
| M2c164272789 | 2 | 164272789 | [A/G] |
| M2c164881985 | 2 | 164881985 | [A/G] |
| M2c164975838 | 2 | 164975838 | [A/G] |
| M2c165259741 | 2 | 165259741 | [T/C] |
| M2c165615985 | 2 | 165615985 | [T/G] |
| M2c166077898 | 2 | 166077898 | [A/G] |
| M2c167206184 | 2 | 167206184 | [A/G] |
| M2c167327320 | 2 | 167327320 | [A/G] |
| M2c167849762 | 2 | 167849762 | [T/C] |
| M2c168226560 | 2 | 168226560 | [A/C] |
| M2c168536149 | 2 | 168536149 | [A/G] |
| M2c168893167 | 2 | 168893167 | [T/C] |
| M2c169147418 | 2 | 169147418 | [T/C] |
| M2c169494752 | 2 | 169494752 | [A/G] |
| M2c169807630 | 2 | 169807630 | [A/G] |
| M2c170267227 | 2 | 170267227 | [T/G] |
| M2c170493099 | 2 | 170493099 | [A/G] |
| M2c171073358 | 2 | 171073358 | [A/G] |
| M2c171224465 | 2 | 171224465 | [T/C] |
| M2c171734926 | 2 | 171734926 | [T/C] |
| M2c172145280 | 2 | 172145280 | [A/C] |
| M2c172706158 | 2 | 172706158 | [A/G] |
| M2c172844632 | 2 | 172844632 | [A/G] |
| M2c173284092 | 2 | 173284092 | [T/G] |
| M2c173604891 | 2 | 173604891 | [T/G] |
| M2c174103997 | 2 | 174103997 | [A/G] |
| M2c174469669 | 2 | 174469669 | [T/G] |
| M2c174602515 | 2 | 174602515 | [A/G] |
| M2c175092138 | 2 | 175092138 | [A/G] |
| M2c175495734 | 2 | 175495734 | [A/G] |
| M2c175831102 | 2 | 175831102 | [A/G] |
| M2c176020325 | 2 | 176020325 | [A/C] |
| M2c176444327 | 2 | 176444327 | [T/C] |
| M2c176641956 | 2 | 176641956 | [A/G] |
| M2c177108725 | 2 | 177108725 | [A/G] |
| M2c178231307 | 2 | 178231307 | [A/G] |
| M2c178382502 | 2 | 178382502 | [T/G] |
| M2c178878009 | 2 | 178878009 | [A/C] |
| M2c179040162 | 2 | 179040162 | [A/G] |
| M2c179809759 | 2 | 179809759 | [T/C] |
| M2c180241833 | 2 | 180241833 | [T/C] |
| M2c180626735 | 2 | 180626735 | [A/G] |
| M2c180892373 | 2 | 180892373 | [T/C] |
| M2c181111259 | 2 | 181111259 | [A/C] |
| M2c181540971 | 2 | 181540971 | [T/G] |
| M2c182343469 | 2 | 182343469 | [T/C] |
| M2c182656825 | 2 | 182656825 | [A/G] |
| M2c182873748 | 2 | 182873748 | [T/C] |
| M2c183184969 | 2 | 183184969 | [T/G] |
| M2c183560638 | 2 | 183560638 | [A/C] |
| M2c183934949 | 2 | 183934949 | [T/C] |
| M2c184144634 | 2 | 184144634 | [A/G] |
| M2c185110911 | 2 | 185110911 | [A/G] |
| M2c185218016 | 2 | 185218016 | [A/C] |
| M2c185693530 | 2 | 185693530 | [A/C] |
| M2c186439174 | 2 | 186439174 | [A/G] |
| M2c186637130 | 2 | 186637130 | [T/C] |
| M2c187052110 | 2 | 187052110 | [T/G] |
| M2c187374195 | 2 | 187374195 | [A/G] |
| M2c187682881 | 2 | 187682881 | [T/C] |
| M2c188162404 | 2 | 188162404 | [A/C] |
| M2c188529995 | 2 | 188529995 | [T/C] |
| M2c188874848 | 2 | 188874848 | [T/C] |
| M2c189088535 | 2 | 189088535 | [T/C] |
| M2c189555793 | 2 | 189555793 | [T/C] |
| M2c189820721 | 2 | 189820721 | [T/C] |
| M2c190024742 | 2 | 190024742 | [A/G] |
| M2c190422041 | 2 | 190422041 | [T/G] |
| M2c190934119 | 2 | 190934119 | [T/C] |
| M2c191002402 | 2 | 191002402 | [T/G] |
| M2c191664821 | 2 | 191664821 | [T/C] |
| M2c191783532 | 2 | 191783532 | [T/C] |
| M2c191818014 | 2 | 191818014 | [T/C] |
| M2c192126251 | 2 | 192126251 | [T/C] |
| M2c192603879 | 2 | 192603879 | [T/C] |
| M2c193497277 | 2 | 193497277 | [T/G] |
| M2c193833217 | 2 | 193833217 | [T/G] |
| M2c194356405 | 2 | 194356405 | [T/G] |
| M2c194480026 | 2 | 194480026 | [T/C] |
| M2c194909734 | 2 | 194909734 | [T/C] |
| M2c195358324 | 2 | 195358324 | [T/C] |
| M2c195759394 | 2 | 195759394 | [A/C] |
| M2c196386747 | 2 | 196386747 | [T/G] |
| M2c196524893 | 2 | 196524893 | [A/G] |
| M2c196873052 | 2 | 196873052 | [A/C] |
| M2c197225782 | 2 | 197225782 | [A/G] |
| M2c197589086 | 2 | 197589086 | [A/G] |
| M2c198153990 | 2 | 198153990 | [T/G] |
| M2c198512109 | 2 | 198512109 | [A/G] |
| M2c198652144 | 2 | 198652144 | [T/G] |
| M2c199180638 | 2 | 199180638 | [T/C] |
| M2c199387323 | 2 | 199387323 | [A/G] |
| M2c199887539 | 2 | 199887539 | [T/C] |
| M2c200086119 | 2 | 200086119 | [T/C] |
| M2c200577812 | 2 | 200577812 | [T/C] |
| M2c200909093 | 2 | 200909093 | [T/C] |
| M2c201207084 | 2 | 201207084 | [T/C] |
| M2c201594641 | 2 | 201594641 | [A/G] |
| M2c201684814 | 2 | 201684814 | [T/C] |
| M2c202284224 | 2 | 202284224 | [A/G] |
| M2c202315030 | 2 | 202315030 | [A/G] |
| M2c202921198 | 2 | 202921198 | [A/G] |
| M2c203063983 | 2 | 203063983 | [A/G] |
| M2c203603076 | 2 | 203603076 | [T/C] |
| M2c203900165 | 2 | 203900165 | [T/C] |
| M2c204485630 | 2 | 204485630 | [A/G] |
| M2c205709373 | 2 | 205709373 | [T/G] |
| M2c206081821 | 2 | 206081821 | [A/G] |
| M2c206418982 | 2 | 206418982 | [T/C] |
| M2c206540172 | 2 | 206540172 | [A/G] |
| M2c206900007 | 2 | 206900007 | [T/C] |
| M2c207236774 | 2 | 207236774 | [A/G] |
| M2c207698287 | 2 | 207698287 | [T/C] |
| M2c208130064 | 2 | 208130064 | [A/G] |
| M2c208226103 | 2 | 208226103 | [T/C] |
| M2c208576953 | 2 | 208576953 | [A/G] |
| M2c209011873 | 2 | 209011873 | [A/G] |
| M2c209196699 | 2 | 209196699 | [T/G] |
| M2c209999211 | 2 | 209999211 | [A/G] |
| M2c210313951 | 2 | 210313951 | [T/C] |
| M2c210788297 | 2 | 210788297 | [T/C] |
| M2c211212730 | 2 | 211212730 | [A/G] |
| M2c211393932 | 2 | 211393932 | [T/C] |
| M2c211734119 | 2 | 211734119 | [A/G] |
| M2c212246084 | 2 | 212246084 | [A/G] |
| M2c212405484 | 2 | 212405484 | [A/C] |
| M2c212804940 | 2 | 212804940 | [T/G] |
| M2c213189165 | 2 | 213189165 | [T/G] |
| M2c213580252 | 2 | 213580252 | [A/G] |
| M2c213952066 | 2 | 213952066 | [A/G] |
| M2c214296282 | 2 | 214296282 | [T/C] |
| M2c214614103 | 2 | 214614103 | [A/G] |
| M2c214952224 | 2 | 214952224 | [A/G] |
| M2c215292126 | 2 | 215292126 | [A/G] |
| M2c216037494 | 2 | 216037494 | [A/G] |
| M2c216190694 | 2 | 216190694 | [A/C] |
| M2c217007767 | 2 | 217007767 | [A/G] |
| M2c217119828 | 2 | 217119828 | [T/C] |
| M2c217640889 | 2 | 217640889 | [A/G] |
| M2c218035540 | 2 | 218035540 | [A/G] |
| M2c218342577 | 2 | 218342577 | [T/C] |
| M2c218584638 | 2 | 218584638 | [T/C] |
| M2c219124327 | 2 | 219124327 | [T/C] |
| M2c219258126 | 2 | 219258126 | [T/C] |
| M2c219729205 | 2 | 219729205 | [A/C] |
| M2c220077584 | 2 | 220077584 | [A/G] |
| M2c220496189 | 2 | 220496189 | [A/G] |
| M2c220670857 | 2 | 220670857 | [T/C] |
| M2c221130024 | 2 | 221130024 | [T/C] |
| M2c221742816 | 2 | 221742816 | [A/C] |
| M2c221894881 | 2 | 221894881 | [A/G] |
| M2c222303652 | 2 | 222303652 | [A/G] |
| M2c222588314 | 2 | 222588314 | [A/G] |
| M2c223035344 | 2 | 223035344 | [T/C] |
| M2c223296606 | 2 | 223296606 | [T/C] |
| M2c223721472 | 2 | 223721472 | [T/C] |
| M2c224064463 | 2 | 224064463 | [A/C] |
| M2c224546805 | 2 | 224546805 | [A/G] |
| M2c224948148 | 2 | 224948148 | [T/C] |
| M2c225444336 | 2 | 225444336 | [A/G] |
| M2c225682322 | 2 | 225682322 | [A/G] |
| M2c226323250 | 2 | 226323250 | [T/C] |
| M2c227356143 | 2 | 227356143 | [T/C] |
| M2c227531266 | 2 | 227531266 | [A/C] |
| M2c228045842 | 2 | 228045842 | [A/G] |
| M2c228048348 | 2 | 228048348 | [A/G] |
| M2c228517785 | 2 | 228517785 | [T/C] |
| M2c229134078 | 2 | 229134078 | [A/C] |
| M2c229479557 | 2 | 229479557 | [A/G] |
| M2c229999195 | 2 | 229999195 | [T/C] |
| M2c230357107 | 2 | 230357107 | [A/G] |
| M2c230586425 | 2 | 230586425 | [A/G] |
| M2c230866649 | 2 | 230866649 | [T/G] |
| M2c231380389 | 2 | 231380389 | [A/G] |
| M2c231660579 | 2 | 231660579 | [A/G] |
| M2c231712426 | 2 | 231712426 | [A/C] |
| M2c231987444 | 2 | 231987444 | [A/C] |
| M2c232368205 | 2 | 232368205 | [T/G] |
| M2c233122578 | 2 | 233122578 | [A/G] |
| M2c233482861 | 2 | 233482861 | [A/G] |
| M2c233798292 | 2 | 233798292 | [T/C] |
| M2c234220417 | 2 | 234220417 | [A/G] |
| M2c234227225 | 2 | 234227225 | [A/G] |
| M2c234726208 | 2 | 234726208 | [T/C] |
| M2c235142522 | 2 | 235142522 | [T/C] |
| M2c235347961 | 2 | 235347961 | [A/C] |
| M2c235853874 | 2 | 235853874 | [T/C] |
| M2c236188999 | 2 | 236188999 | [T/G] |
| M2c236284951 | 2 | 236284951 | [A/G] |
| M2c236814886 | 2 | 236814886 | [A/G] |
| M2c236971222 | 2 | 236971222 | [A/C] |
| M3c470965 | 3 | 470965 | [T/C] |
| M3c655716 | 3 | 655716 | [A/G] |
| M3c989725 | 3 | 989725 | [A/G] |
| M3c1481059 | 3 | 1481059 | [A/G] |
| M3c1627346 | 3 | 1627346 | [T/C] |
| M3c2079025 | 3 | 2079025 | [A/G] |
| M3c2743270 | 3 | 2743270 | [A/G] |
| M3c3208487 | 3 | 3208487 | [T/C] |
| M3c3560282 | 3 | 3560282 | [T/C] |
| M3c3841571 | 3 | 3841571 | [A/G] |
| M3c4350288 | 3 | 4350288 | [A/G] |
| M3c4636668 | 3 | 4636668 | [A/G] |
| M3c4710998 | 3 | 4710998 | [A/C] |
| M3c5322131 | 3 | 5322131 | [T/C] |
| M3c5434945 | 3 | 5434945 | [T/C] |
| M3c5675783 | 3 | 5675783 | [A/G] |
| M3c5751247 | 3 | 5751247 | [T/C] |
| M3c5765238 | 3 | 5765238 | [A/C] |
| M3c6095716 | 3 | 6095716 | [T/G] |
| M3c6523471 | 3 | 6523471 | [T/G] |
| M3c6795219 | 3 | 6795219 | [A/G] |
| M3c7685102 | 3 | 7685102 | [T/C] |
| M3c8122289 | 3 | 8122289 | [A/C] |
| M3c8272789 | 3 | 8272789 | [T/C] |
| M3c8602550 | 3 | 8602550 | [T/C] |
| M3c8882280 | 3 | 8882280 | [T/G] |
| M3c9580892 | 3 | 9580892 | [A/G] |
| M3c10179987 | 3 | 10179987 | [A/G] |
| M3c10221041 | 3 | 10221041 | [A/G] |
| M3c10607873 | 3 | 10607873 | [T/C] |
| M3c11007387 | 3 | 11007387 | [A/C] |
| M3c11897617 | 3 | 11897617 | [A/C] |
| M3c12192230 | 3 | 12192230 | [T/C] |
| M3c12290085 | 3 | 12290085 | [A/C] |
| M3c12802465 | 3 | 12802465 | [A/G] |
| M3c13277867 | 3 | 13277867 | [A/G] |
| M3c13279768 | 3 | 13279768 | [A/G] |
| M3c13731012 | 3 | 13731012 | [T/G] |
| M3c14109926 | 3 | 14109926 | [A/G] |
| M3c14470044 | 3 | 14470044 | [A/G] |
| M3c14849506 | 3 | 14849506 | [T/C] |
| M3c15427752 | 3 | 15427752 | [T/C] |
| M3c15913139 | 3 | 15913139 | [T/C] |
| M3c16468512 | 3 | 16468512 | [A/G] |
| M3c17003825 | 3 | 17003825 | [A/G] |
| M3c17284235 | 3 | 17284235 | [A/G] |
| M3c17608001 | 3 | 17608001 | [A/G] |
| M3c18028330 | 3 | 18028330 | [A/G] |
| M3c18319066 | 3 | 18319066 | [A/G] |
| M3c18637400 | 3 | 18637400 | [T/G] |
| M3c18854665 | 3 | 18854665 | [A/C] |
| M3c19255701 | 3 | 19255701 | [T/C] |
| M3c19529556 | 3 | 19529556 | [T/G] |
| M3c19942395 | 3 | 19942395 | [A/G] |
| M3c20417247 | 3 | 20417247 | [A/G] |
| M3c20587499 | 3 | 20587499 | [A/G] |
| M3c21144834 | 3 | 21144834 | [T/C] |
| M3c21455365 | 3 | 21455365 | [A/G] |
| M3c21849012 | 3 | 21849012 | [T/C] |
| M3c22389425 | 3 | 22389425 | [T/G] |
| M3c22572781 | 3 | 22572781 | [T/C] |
| M3c23012451 | 3 | 23012451 | [A/G] |
| M3c23433316 | 3 | 23433316 | [T/C] |
| M3c23846812 | 3 | 23846812 | [T/C] |
| M3c24353398 | 3 | 24353398 | [A/C] |
| M3c24917748 | 3 | 24917748 | [T/G] |
| M3c25215105 | 3 | 25215105 | [A/G] |
| M3c25355371 | 3 | 25355371 | [A/C] |
| M3c25862727 | 3 | 25862727 | [A/G] |
| M3c26101402 | 3 | 26101402 | [A/G] |
| M3c26448058 | 3 | 26448058 | [T/G] |
| M3c27317113 | 3 | 27317113 | [T/C] |
| M3c27558730 | 3 | 27558730 | [A/G] |
| M3c27859909 | 3 | 27859909 | [T/G] |
| M3c28334398 | 3 | 28334398 | [T/C] |
| M3c28620669 | 3 | 28620669 | [T/C] |
| M3c29621207 | 3 | 29621207 | [T/C] |
| M3c29978514 | 3 | 29978514 | [A/G] |
| M3c30139159 | 3 | 30139159 | [T/C] |
| M3c30440296 | 3 | 30440296 | [A/C] |
| M3c30997705 | 3 | 30997705 | [A/C] |
| M3c31556788 | 3 | 31556788 | [A/G] |
| M3c32043963 | 3 | 32043963 | [T/C] |
| M3c32246440 | 3 | 32246440 | [T/G] |
| M3c32780376 | 3 | 32780376 | [A/G] |
| M3c33061321 | 3 | 33061321 | [A/G] |
| M3c33503069 | 3 | 33503069 | [T/C] |
| M3c34497373 | 3 | 34497373 | [T/C] |
| M3c34670876 | 3 | 34670876 | [T/C] |
| M3c34948388 | 3 | 34948388 | [T/G] |
| M3c35429244 | 3 | 35429244 | [T/G] |
| M3c35750177 | 3 | 35750177 | [A/G] |
| M3c36226661 | 3 | 36226661 | [T/G] |
| M3c36310766 | 3 | 36310766 | [A/G] |
| M3c36833272 | 3 | 36833272 | [A/G] |
| M3c37163651 | 3 | 37163651 | [A/G] |
| M3c37671940 | 3 | 37671940 | [A/G] |
| M3c38167419 | 3 | 38167419 | [T/G] |
| M3c38428248 | 3 | 38428248 | [A/G] |
| M3c38744133 | 3 | 38744133 | [T/G] |
| M3c39217849 | 3 | 39217849 | [A/C] |
| M3c39475330 | 3 | 39475330 | [T/G] |
| M3c39747466 | 3 | 39747466 | [A/C] |
| M3c40321869 | 3 | 40321869 | [A/G] |
| M3c40579173 | 3 | 40579173 | [T/C] |
| M3c40829658 | 3 | 40829658 | [T/C] |
| M3c41403869 | 3 | 41403869 | [A/G] |
| M3c41454070 | 3 | 41454070 | [A/G] |
| M3c42259523 | 3 | 42259523 | [A/C] |
| M3c42550574 | 3 | 42550574 | [A/G] |
| M3c43230600 | 3 | 43230600 | [T/C] |
| M3c43658787 | 3 | 43658787 | [A/C] |
| M3c43992235 | 3 | 43992235 | [A/G] |
| M3c44339760 | 3 | 44339760 | [T/C] |
| M3c44672731 | 3 | 44672731 | [A/G] |
| M3c45053604 | 3 | 45053604 | [A/G] |
| M3c45205248 | 3 | 45205248 | [A/G] |
| M3c45791696 | 3 | 45791696 | [A/G] |
| M3c46179055 | 3 | 46179055 | [T/C] |
| M3c46538500 | 3 | 46538500 | [T/C] |
| M3c46883529 | 3 | 46883529 | [T/C] |
| M3c47212646 | 3 | 47212646 | [A/C] |
| M3c47328847 | 3 | 47328847 | [T/C] |
| M3c47850740 | 3 | 47850740 | [T/C] |
| M3c48042531 | 3 | 48042531 | [T/G] |
| M3c48494238 | 3 | 48494238 | [A/G] |
| M3c49181181 | 3 | 49181181 | [A/G] |
| M3c49384261 | 3 | 49384261 | [A/G] |
| M3c49864666 | 3 | 49864666 | [A/G] |
| M3c50606672 | 3 | 50606672 | [T/G] |
| M3c51137448 | 3 | 51137448 | [A/G] |
| M3c51466316 | 3 | 51466316 | [A/G] |
| M3c52248934 | 3 | 52248934 | [T/C] |
| M3c53143525 | 3 | 53143525 | [A/C] |
| M3c53488512 | 3 | 53488512 | [T/C] |
| M3c54117186 | 3 | 54117186 | [A/C] |
| M3c54840781 | 3 | 54840781 | [A/C] |
| M3c55802944 | 3 | 55802944 | [A/G] |
| M3c56290275 | 3 | 56290275 | [A/G] |
| M3c57098850 | 3 | 57098850 | [T/C] |
| M3c57231184 | 3 | 57231184 | [T/C] |
| M3c57658674 | 3 | 57658674 | [T/G] |
| M3c58081419 | 3 | 58081419 | [T/G] |
| M3c58540879 | 3 | 58540879 | [T/C] |
| M3c59297467 | 3 | 59297467 | [T/C] |
| M3c59843600 | 3 | 59843600 | [T/C] |
| M3c60254604 | 3 | 60254604 | [A/G] |
| M3c60574360 | 3 | 60574360 | [T/C] |
| M3c60694552 | 3 | 60694552 | [T/C] |
| M3c61241333 | 3 | 61241333 | [A/C] |
| M3c61340900 | 3 | 61340900 | [T/C] |
| M3c61844814 | 3 | 61844814 | [T/C] |
| M3c62179345 | 3 | 62179345 | [A/G] |
| M3c63108464 | 3 | 63108464 | [T/G] |
| M3c63696378 | 3 | 63696378 | [A/G] |
| M3c63963622 | 3 | 63963622 | [A/G] |
| M3c64264338 | 3 | 64264338 | [T/C] |
| M3c64461335 | 3 | 64461335 | [T/C] |
| M3c64819072 | 3 | 64819072 | [A/C] |
| M3c65286247 | 3 | 65286247 | [A/C] |
| M3c65663672 | 3 | 65663672 | [A/G] |
| M3c66110056 | 3 | 66110056 | [T/C] |
| M3c66187197 | 3 | 66187197 | [A/C] |
| M3c66705321 | 3 | 66705321 | [A/G] |
| M3c66944550 | 3 | 66944550 | [A/C] |
| M3c67258345 | 3 | 67258345 | [T/C] |
| M3c67923638 | 3 | 67923638 | [A/G] |
| M3c68359000 | 3 | 68359000 | [T/C] |
| M3c68751901 | 3 | 68751901 | [A/C] |
| M3c69144010 | 3 | 69144010 | [T/C] |
| M3c69204608 | 3 | 69204608 | [T/C] |
| M3c69953052 | 3 | 69953052 | [A/G] |
| M3c70702132 | 3 | 70702132 | [T/C] |
| M3c71308750 | 3 | 71308750 | [T/C] |
| M3c71873824 | 3 | 71873824 | [T/C] |
| M3c72285566 | 3 | 72285566 | [T/C] |
| M3c72532638 | 3 | 72532638 | [T/C] |
| M3c72770054 | 3 | 72770054 | [T/C] |
| M3c73311237 | 3 | 73311237 | [A/G] |
| M3c73647174 | 3 | 73647174 | [T/C] |
| M3c73982699 | 3 | 73982699 | [A/G] |
| M3c74158700 | 3 | 74158700 | [A/G] |
| M3c74583159 | 3 | 74583159 | [T/C] |
| M3c75047065 | 3 | 75047065 | [T/C] |
| M3c75499985 | 3 | 75499985 | [A/G] |
| M3c75956341 | 3 | 75956341 | [A/G] |
| M3c76579313 | 3 | 76579313 | [A/G] |
| M3c77152481 | 3 | 77152481 | [T/C] |
| M3c77640828 | 3 | 77640828 | [A/G] |
| M3c78200323 | 3 | 78200323 | [T/C] |
| M3c78497275 | 3 | 78497275 | [T/C] |
| M3c79609017 | 3 | 79609017 | [T/C] |
| M3c80146066 | 3 | 80146066 | [A/G] |
| M3c80348747 | 3 | 80348747 | [A/G] |
| M3c81074929 | 3 | 81074929 | [A/G] |
| M3c81531039 | 3 | 81531039 | [A/G] |
| M3c81705591 | 3 | 81705591 | [A/C] |
| M3c82021447 | 3 | 82021447 | [A/G] |
| M3c82352151 | 3 | 82352151 | [A/G] |
| M3c82727904 | 3 | 82727904 | [T/G] |
| M3c83049862 | 3 | 83049862 | [A/C] |
| M3c83274750 | 3 | 83274750 | [T/C] |
| M3c84006864 | 3 | 84006864 | [A/G] |
| M3c84439071 | 3 | 84439071 | [A/G] |
| M3c84909559 | 3 | 84909559 | [A/G] |
| M3c85057381 | 3 | 85057381 | [A/C] |
| M3c85663397 | 3 | 85663397 | [T/C] |
| M3c85751521 | 3 | 85751521 | [A/G] |
| M3c86359145 | 3 | 86359145 | [T/C] |
| M3c86902257 | 3 | 86902257 | [T/C] |
| M3c87211600 | 3 | 87211600 | [T/C] |
| M3c87424807 | 3 | 87424807 | [T/C] |
| M3c87808945 | 3 | 87808945 | [A/G] |
| M3c88391049 | 3 | 88391049 | [A/G] |
| M3c88516679 | 3 | 88516679 | [T/C] |
| M3c89043914 | 3 | 89043914 | [T/C] |
| M3c89686547 | 3 | 89686547 | [A/G] |
| M3c90025426 | 3 | 90025426 | [T/C] |
| M3c90411023 | 3 | 90411023 | [A/C] |
| M3c90704054 | 3 | 90704054 | [A/G] |
| M3c91095447 | 3 | 91095447 | [T/C] |
| M3c91245521 | 3 | 91245521 | [T/C] |
| M3c91677989 | 3 | 91677989 | [T/C] |
| M3c91935448 | 3 | 91935448 | [A/G] |
| M3c92221848 | 3 | 92221848 | [A/G] |
| M3c92542170 | 3 | 92542170 | [T/G] |
| M3c93185590 | 3 | 93185590 | [A/G] |
| M3c93312427 | 3 | 93312427 | [T/G] |
| M3c93827663 | 3 | 93827663 | [A/C] |
| M3c93988247 | 3 | 93988247 | [A/G] |
| M3c94334082 | 3 | 94334082 | [T/C] |
| M3c94801478 | 3 | 94801478 | [T/C] |
| M3c95266356 | 3 | 95266356 | [A/G] |
| M3c95512200 | 3 | 95512200 | [T/C] |
| M3c95859983 | 3 | 95859983 | [T/G] |
| M3c96237916 | 3 | 96237916 | [A/G] |
| M3c96366130 | 3 | 96366130 | [T/C] |
| M3c96706581 | 3 | 96706581 | [A/C] |
| M3c97582739 | 3 | 97582739 | [A/G] |
| M3c98011575 | 3 | 98011575 | [T/C] |
| M3c98140408 | 3 | 98140408 | [T/C] |
| M3c98496970 | 3 | 98496970 | [T/C] |
| M3c98799672 | 3 | 98799672 | [A/G] |
| M3c99280448 | 3 | 99280448 | [A/G] |
| M3c99727791 | 3 | 99727791 | [A/G] |
| M3c100079905 | 3 | 100079905 | [A/G] |
| M3c100284463 | 3 | 100284463 | [T/C] |
| M3c100434597 | 3 | 100434597 | [A/G] |
| M3c100844491 | 3 | 100844491 | [A/G] |
| M3c101382589 | 3 | 101382589 | [A/C] |
| M3c101701006 | 3 | 101701006 | [A/G] |
| M3c101955115 | 3 | 101955115 | [T/C] |
| M3c102217354 | 3 | 102217354 | [A/G] |
| M3c102488991 | 3 | 102488991 | [A/G] |
| M3c103166745 | 3 | 103166745 | [T/C] |
| M3c103326531 | 3 | 103326531 | [A/C] |
| M3c103544700 | 3 | 103544700 | [T/C] |
| M3c104044994 | 3 | 104044994 | [A/G] |
| M3c104753320 | 3 | 104753320 | [T/C] |
| M3c104894981 | 3 | 104894981 | [A/G] |
| M3c105347231 | 3 | 105347231 | [A/G] |
| M3c105645506 | 3 | 105645506 | [A/C] |
| M3c106244719 | 3 | 106244719 | [A/G] |
| M3c106810243 | 3 | 106810243 | [A/C] |
| M3c106957725 | 3 | 106957725 | [A/G] |
| M3c107892511 | 3 | 107892511 | [A/G] |
| M3c108145074 | 3 | 108145074 | [A/G] |
| M3c108555340 | 3 | 108555340 | [T/C] |
| M3c108732252 | 3 | 108732252 | [T/C] |
| M3c109187908 | 3 | 109187908 | [T/C] |
| M3c109368694 | 3 | 109368694 | [T/C] |
| M3c110214487 | 3 | 110214487 | [T/G] |
| M3c110461378 | 3 | 110461378 | [T/C] |
| M3c110808517 | 3 | 110808517 | [A/G] |
| M3c111229238 | 3 | 111229238 | [A/G] |
| M3c112033929 | 3 | 112033929 | [A/G] |
| M3c112198441 | 3 | 112198441 | [A/G] |
| M3c112757329 | 3 | 112757329 | [A/C] |
| M3c113069015 | 3 | 113069015 | [A/C] |
| M3c113348728 | 3 | 113348728 | [A/C] |
| M3c113594215 | 3 | 113594215 | [A/G] |
| M3c114020972 | 3 | 114020972 | [T/C] |
| M3c114353795 | 3 | 114353795 | [T/G] |
| M3c114787315 | 3 | 114787315 | [T/C] |
| M3c115006037 | 3 | 115006037 | [T/C] |
| M3c115308283 | 3 | 115308283 | [A/G] |
| M3c115793337 | 3 | 115793337 | [A/G] |
| M3c116026935 | 3 | 116026935 | [T/C] |
| M3c116553323 | 3 | 116553323 | [T/C] |
| M3c117089716 | 3 | 117089716 | [T/G] |
| M3c117259226 | 3 | 117259226 | [A/G] |
| M3c117591502 | 3 | 117591502 | [A/C] |
| M3c118173382 | 3 | 118173382 | [T/C] |
| M3c118833919 | 3 | 118833919 | [A/C] |
| M3c119179711 | 3 | 119179711 | [A/G] |
| M3c119327441 | 3 | 119327441 | [T/G] |
| M3c119977155 | 3 | 119977155 | [T/G] |
| M3c120190805 | 3 | 120190805 | [T/C] |
| M3c120346395 | 3 | 120346395 | [A/C] |
| M3c120748733 | 3 | 120748733 | [T/C] |
| M3c121071496 | 3 | 121071496 | [A/G] |
| M3c121623478 | 3 | 121623478 | [T/C] |
| M3c121718665 | 3 | 121718665 | [T/C] |
| M3c122066758 | 3 | 122066758 | [T/C] |
| M3c122883097 | 3 | 122883097 | [T/C] |
| M3c124097875 | 3 | 124097875 | [A/G] |
| M3c124189168 | 3 | 124189168 | [T/C] |
| M3c124732596 | 3 | 124732596 | [T/C] |
| M3c124922809 | 3 | 124922809 | [A/G] |
| M3c125227350 | 3 | 125227350 | [A/G] |
| M3c125480513 | 3 | 125480513 | [A/G] |
| M3c126109305 | 3 | 126109305 | [A/G] |
| M3c126569064 | 3 | 126569064 | [T/C] |
| M3c127008564 | 3 | 127008564 | [A/G] |
| M3c127512845 | 3 | 127512845 | [A/C] |
| M3c127789690 | 3 | 127789690 | [T/C] |
| M3c128099285 | 3 | 128099285 | [T/G] |
| M3c128232952 | 3 | 128232952 | [A/G] |
| M3c128663479 | 3 | 128663479 | [T/C] |
| M3c128935021 | 3 | 128935021 | [T/C] |
| M3c129304528 | 3 | 129304528 | [A/G] |
| M3c129616115 | 3 | 129616115 | [T/C] |
| M3c130098183 | 3 | 130098183 | [A/G] |
| M3c130519333 | 3 | 130519333 | [A/G] |
| M3c130829687 | 3 | 130829687 | [A/C] |
| M3c131014335 | 3 | 131014335 | [T/G] |
| M3c131605545 | 3 | 131605545 | [A/G] |
| M3c131931505 | 3 | 131931505 | [T/G] |
| M3c132263592 | 3 | 132263592 | [T/C] |
| M3c132842698 | 3 | 132842698 | [A/G] |
| M3c133205879 | 3 | 133205879 | [T/C] |
| M3c133454822 | 3 | 133454822 | [A/G] |
| M3c134250398 | 3 | 134250398 | [A/G] |
| M3c134615000 | 3 | 134615000 | [T/C] |
| M3c135303199 | 3 | 135303199 | [A/C] |
| M3c135509233 | 3 | 135509233 | [T/G] |
| M3c135852239 | 3 | 135852239 | [A/G] |
| M3c136333692 | 3 | 136333692 | [A/C] |
| M3c136568007 | 3 | 136568007 | [T/C] |
| M3c136974999 | 3 | 136974999 | [T/C] |
| M3c137680670 | 3 | 137680670 | [A/G] |
| M3c138122181 | 3 | 138122181 | [A/C] |
| M3c138386380 | 3 | 138386380 | [T/C] |
| M3c138778128 | 3 | 138778128 | [T/C] |
| M3c139220347 | 3 | 139220347 | [A/C] |
| M3c139809923 | 3 | 139809923 | [T/C] |
| M3c140099444 | 3 | 140099444 | [A/G] |
| M3c140463431 | 3 | 140463431 | [T/C] |
| M3c140726053 | 3 | 140726053 | [T/C] |
| M3c140978009 | 3 | 140978009 | [A/C] |
| M3c141561352 | 3 | 141561352 | [A/G] |
| M3c141699112 | 3 | 141699112 | [T/C] |
| M3c142249775 | 3 | 142249775 | [T/C] |
| M3c142424381 | 3 | 142424381 | [T/C] |
| M3c142692824 | 3 | 142692824 | [T/C] |
| M3c143001352 | 3 | 143001352 | [A/G] |
| M3c143488417 | 3 | 143488417 | [T/C] |
| M3c144094298 | 3 | 144094298 | [T/G] |
| M3c144416264 | 3 | 144416264 | [T/G] |
| M3c144687873 | 3 | 144687873 | [T/C] |
| M3c145272582 | 3 | 145272582 | [A/G] |
| M3c145480205 | 3 | 145480205 | [A/G] |
| M3c145764922 | 3 | 145764922 | [A/G] |
| M3c146250918 | 3 | 146250918 | [T/G] |
| M3c147016334 | 3 | 147016334 | [T/C] |
| M3c147404002 | 3 | 147404002 | [A/G] |
| M3c147622083 | 3 | 147622083 | [T/C] |
| M3c148098044 | 3 | 148098044 | [T/C] |
| M3c148613501 | 3 | 148613501 | [A/C] |
| M3c148841922 | 3 | 148841922 | [A/G] |
| M3c149232161 | 3 | 149232161 | [A/C] |
| M3c149563451 | 3 | 149563451 | [A/C] |
| M3c150169162 | 3 | 150169162 | [T/C] |
| M3c150367947 | 3 | 150367947 | [A/G] |
| M3c150588960 | 3 | 150588960 | [A/G] |
| M3c151046873 | 3 | 151046873 | [T/C] |
| M3c151357006 | 3 | 151357006 | [T/G] |
| M3c151633270 | 3 | 151633270 | [T/C] |
| M3c152184405 | 3 | 152184405 | [A/G] |
| M3c152494658 | 3 | 152494658 | [T/C] |
| M3c152845710 | 3 | 152845710 | [A/G] |
| M3c153258770 | 3 | 153258770 | [T/G] |
| M3c153298314 | 3 | 153298314 | [A/C] |
| M3c153764734 | 3 | 153764734 | [A/G] |
| M3c154088531 | 3 | 154088531 | [A/G] |
| M3c154593950 | 3 | 154593950 | [A/G] |
| M3c154976720 | 3 | 154976720 | [T/C] |
| M3c154979694 | 3 | 154979694 | [A/G] |
| M3c155960629 | 3 | 155960629 | [A/G] |
| M3c156061008 | 3 | 156061008 | [T/C] |
| M3c156433838 | 3 | 156433838 | [A/C] |
| M3c156797172 | 3 | 156797172 | [T/G] |
| M3c157759194 | 3 | 157759194 | [T/C] |
| M3c158202416 | 3 | 158202416 | [A/C] |
| M3c158892231 | 3 | 158892231 | [A/G] |
| M3c159378411 | 3 | 159378411 | [A/C] |
| M3c159526813 | 3 | 159526813 | [A/G] |
| M3c160014443 | 3 | 160014443 | [T/C] |
| M3c160303648 | 3 | 160303648 | [A/C] |
| M3c160670930 | 3 | 160670930 | [T/C] |
| M3c160899465 | 3 | 160899465 | [A/G] |
| M3c161369503 | 3 | 161369503 | [T/G] |
| M3c162827249 | 3 | 162827249 | [T/C] |
| M3c163086332 | 3 | 163086332 | [A/G] |
| M3c163712420 | 3 | 163712420 | [T/C] |
| M3c163995945 | 3 | 163995945 | [T/C] |
| M3c164480993 | 3 | 164480993 | [T/C] |
| M3c164884525 | 3 | 164884525 | [A/G] |
| M3c164969911 | 3 | 164969911 | [T/C] |
| M3c165462332 | 3 | 165462332 | [T/C] |
| M3c165911994 | 3 | 165911994 | [T/C] |
| M3c166247161 | 3 | 166247161 | [A/G] |
| M3c166449550 | 3 | 166449550 | [A/G] |
| M3c166799624 | 3 | 166799624 | [A/C] |
| M3c167007181 | 3 | 167007181 | [T/C] |
| M3c167474886 | 3 | 167474886 | [T/C] |
| M3c167690341 | 3 | 167690341 | [T/C] |
| M3c168012590 | 3 | 168012590 | [T/C] |
| M3c168233750 | 3 | 168233750 | [A/C] |
| M3c168298873 | 3 | 168298873 | [A/G] |
| M3c168482460 | 3 | 168482460 | [T/C] |
| M3c168487642 | 3 | 168487642 | [T/C] |
| M3c168492566 | 3 | 168492566 | [A/G] |
| M3c168859850 | 3 | 168859850 | [T/G] |
| M3c168860624 | 3 | 168860624 | [A/G] |
| M3c169730362 | 3 | 169730362 | [T/C] |
| M3c169874341 | 3 | 169874341 | [A/C] |
| M3c170441940 | 3 | 170441940 | [A/G] |
| M3c171438379 | 3 | 171438379 | [T/C] |
| M3c171715945 | 3 | 171715945 | [T/C] |
| M3c172051041 | 3 | 172051041 | [A/G] |
| M3c172624243 | 3 | 172624243 | [A/G] |
| M3c173002258 | 3 | 173002258 | [A/G] |
| M3c173473757 | 3 | 173473757 | [T/C] |
| M3c173737994 | 3 | 173737994 | [T/C] |
| M3c174054354 | 3 | 174054354 | [A/G] |
| M3c174367916 | 3 | 174367916 | [A/C] |
| M3c174826064 | 3 | 174826064 | [T/C] |
| M3c175157179 | 3 | 175157179 | [A/G] |
| M3c175560221 | 3 | 175560221 | [T/C] |
| M3c175897784 | 3 | 175897784 | [A/G] |
| M3c175966460 | 3 | 175966460 | [A/G] |
| M3c176525480 | 3 | 176525480 | [T/C] |
| M3c176677313 | 3 | 176677313 | [T/G] |
| M3c177372763 | 3 | 177372763 | [A/G] |
| M3c177788811 | 3 | 177788811 | [A/C] |
| M3c178049748 | 3 | 178049748 | [T/C] |
| M3c178537710 | 3 | 178537710 | [T/C] |
| M3c179264565 | 3 | 179264565 | [T/G] |
| M3c179411625 | 3 | 179411625 | [T/C] |
| M3c179725389 | 3 | 179725389 | [T/C] |
| M3c180199044 | 3 | 180199044 | [A/G] |
| M3c180570197 | 3 | 180570197 | [A/C] |
| M3c180744388 | 3 | 180744388 | [T/C] |
| M3c181071624 | 3 | 181071624 | [A/C] |
| M3c181505973 | 3 | 181505973 | [T/C] |
| M3c182776342 | 3 | 182776342 | [T/C] |
| M3c183262547 | 3 | 183262547 | [A/G] |
| M3c184058938 | 3 | 184058938 | [T/C] |
| M3c184435036 | 3 | 184435036 | [T/G] |
| M3c184814422 | 3 | 184814422 | [A/G] |
| M3c184852073 | 3 | 184852073 | [A/G] |
| M3c185312639 | 3 | 185312639 | [A/C] |
| M3c185837328 | 3 | 185837328 | [A/G] |
| M3c185968145 | 3 | 185968145 | [A/G] |
| M3c186216265 | 3 | 186216265 | [A/G] |
| M3c186785795 | 3 | 186785795 | [A/G] |
| M3c187212622 | 3 | 187212622 | [A/G] |
| M3c187451031 | 3 | 187451031 | [A/G] |
| M3c187712116 | 3 | 187712116 | [T/G] |
| M3c187935218 | 3 | 187935218 | [T/G] |
| M3c188897897 | 3 | 188897897 | [A/C] |
| M3c189240313 | 3 | 189240313 | [T/C] |
| M3c189466935 | 3 | 189466935 | [T/C] |
| M3c190064606 | 3 | 190064606 | [A/G] |
| M3c190324634 | 3 | 190324634 | [A/G] |
| M3c190750009 | 3 | 190750009 | [A/G] |
| M3c191054903 | 3 | 191054903 | [T/C] |
| M3c191054987 | 3 | 191054987 | [A/G] |
| M3c191374469 | 3 | 191374469 | [A/C] |
| M3c191772406 | 3 | 191772406 | [T/C] |
| M3c192400876 | 3 | 192400876 | [T/C] |
| M3c192787863 | 3 | 192787863 | [T/C] |
| M3c194044566 | 3 | 194044566 | [T/G] |
| M3c194256597 | 3 | 194256597 | [T/C] |
| M3c194474558 | 3 | 194474558 | [T/C] |
| M3c194803727 | 3 | 194803727 | [T/G] |
| M3c196075736 | 3 | 196075736 | [A/G] |
| M3c196151369 | 3 | 196151369 | [A/C] |
| M3c196688960 | 3 | 196688960 | [A/G] |
| M3c196942020 | 3 | 196942020 | [T/C] |
| M3c197248877 | 3 | 197248877 | [T/G] |
| M3c197715215 | 3 | 197715215 | [A/G] |
| M3c197947808 | 3 | 197947808 | [A/G] |
| M3c198390555 | 3 | 198390555 | [A/G] |
| M3c199250779 | 3 | 199250779 | [T/C] |
| M3c199834547 | 3 | 199834547 | [A/C] |
| M3c200187411 | 3 | 200187411 | [A/C] |
| M3c200430220 | 3 | 200430220 | [A/G] |
| M3c201783346 | 3 | 201783346 | [T/G] |
| M3c202038436 | 3 | 202038436 | [T/C] |
| M3c202434111 | 3 | 202434111 | [T/C] |
| M3c202838929 | 3 | 202838929 | [T/G] |
| M3c203290535 | 3 | 203290535 | [A/C] |
| M3c203517449 | 3 | 203517449 | [A/G] |
| M3c203759973 | 3 | 203759973 | [A/G] |
| M3c204095588 | 3 | 204095588 | [T/C] |
| M3c204644237 | 3 | 204644237 | [T/C] |
| M3c205017207 | 3 | 205017207 | [A/C] |
| M3c205407013 | 3 | 205407013 | [T/C] |
| M3c205627055 | 3 | 205627055 | [A/G] |
| M3c205972071 | 3 | 205972071 | [A/G] |
| M3c206416566 | 3 | 206416566 | [A/G] |
| M3c206851948 | 3 | 206851948 | [A/G] |
| M3c207170712 | 3 | 207170712 | [A/G] |
| M3c208048742 | 3 | 208048742 | [A/G] |
| M3c208344511 | 3 | 208344511 | [A/C] |
| M3c208828780 | 3 | 208828780 | [A/C] |
| M3c209138037 | 3 | 209138037 | [T/C] |
| M3c209728783 | 3 | 209728783 | [A/G] |
| M3c209973792 | 3 | 209973792 | [A/G] |
| M3c210417004 | 3 | 210417004 | [T/C] |
| M3c210821187 | 3 | 210821187 | [T/C] |
| M3c211016773 | 3 | 211016773 | [A/G] |
| M3c211326037 | 3 | 211326037 | [A/G] |
| M3c211866087 | 3 | 211866087 | [A/G] |
| M3c211977309 | 3 | 211977309 | [T/C] |
| M3c212406628 | 3 | 212406628 | [T/C] |
| M3c212698808 | 3 | 212698808 | [A/C] |
| M3c212983003 | 3 | 212983003 | [A/G] |
| M3c213622390 | 3 | 213622390 | [A/G] |
| M3c213654405 | 3 | 213654405 | [T/C] |
| M3c214307877 | 3 | 214307877 | [A/G] |
| M3c214472004 | 3 | 214472004 | [T/C] |
| M3c214715886 | 3 | 214715886 | [A/G] |
| M3c215466897 | 3 | 215466897 | [A/C] |
| M3c215929810 | 3 | 215929810 | [A/G] |
| M3c216254942 | 3 | 216254942 | [A/G] |
| M3c216464674 | 3 | 216464674 | [A/G] |
| M3c217008927 | 3 | 217008927 | [A/G] |
| M3c217471142 | 3 | 217471142 | [A/G] |
| M3c217896087 | 3 | 217896087 | [T/C] |
| M3c218362066 | 3 | 218362066 | [A/C] |
| M3c218778912 | 3 | 218778912 | [T/C] |
| M3c218898682 | 3 | 218898682 | [A/G] |
| M3c219426148 | 3 | 219426148 | [T/C] |
| M3c219785889 | 3 | 219785889 | [T/C] |
| M3c219842336 | 3 | 219842336 | [T/C] |
| M3c220508548 | 3 | 220508548 | [T/C] |
| M3c220526324 | 3 | 220526324 | [A/G] |
| M3c220984058 | 3 | 220984058 | [A/C] |
| M3c221308758 | 3 | 221308758 | [T/C] |
| M3c221637182 | 3 | 221637182 | [A/G] |
| M3c221923815 | 3 | 221923815 | [T/C] |
| M3c222450979 | 3 | 222450979 | [T/C] |
| M3c222768757 | 3 | 222768757 | [T/C] |
| M3c223083185 | 3 | 223083185 | [A/G] |
| M3c223746191 | 3 | 223746191 | [T/C] |
| M3c224001702 | 3 | 224001702 | [A/C] |
| M3c224460726 | 3 | 224460726 | [A/G] |
| M3c224926023 | 3 | 224926023 | [T/C] |
| M3c225198334 | 3 | 225198334 | [T/C] |
| M3c225456599 | 3 | 225456599 | [A/G] |
| M3c226510221 | 3 | 226510221 | [T/C] |
| M3c226769046 | 3 | 226769046 | [T/C] |
| M3c227047497 | 3 | 227047497 | [A/G] |
| M3c227578705 | 3 | 227578705 | [T/G] |
| M3c227759825 | 3 | 227759825 | [A/C] |
| M3c228370276 | 3 | 228370276 | [A/G] |
| M3c228602166 | 3 | 228602166 | [T/C] |
| M3c228983283 | 3 | 228983283 | [T/C] |
| M3c229388123 | 3 | 229388123 | [T/C] |
| M3c229658588 | 3 | 229658588 | [T/C] |
| M3c229903593 | 3 | 229903593 | [A/G] |
| M3c230437497 | 3 | 230437497 | [A/G] |
| M3c230655495 | 3 | 230655495 | [T/C] |
| M3c231114800 | 3 | 231114800 | [A/G] |
| M3c231826112 | 3 | 231826112 | [A/G] |
| M3c231999311 | 3 | 231999311 | [A/C] |
| M4c140130 | 4 | 140130 | [A/G] |
| M4c515015 | 4 | 515015 | [A/G] |
| M4c812399 | 4 | 812399 | [A/G] |
| M4c1265191 | 4 | 1265191 | [T/G] |
| M4c1458931 | 4 | 1458931 | [A/C] |
| M4c2051266 | 4 | 2051266 | [A/G] |
| M4c2409989 | 4 | 2409989 | [T/C] |
| M4c2515123 | 4 | 2515123 | [T/G] |
| M4c2998895 | 4 | 2998895 | [T/G] |
| M4c3460012 | 4 | 3460012 | [T/G] |
| M4c3749744 | 4 | 3749744 | [T/C] |
| M4c3892482 | 4 | 3892482 | [A/C] |
| M4c5153130 | 4 | 5153130 | [T/C] |
| M4c5518302 | 4 | 5518302 | [A/G] |
| M4c5591119 | 4 | 5591119 | [A/G] |
| M4c6062712 | 4 | 6062712 | [T/C] |
| M4c6416748 | 4 | 6416748 | [T/G] |
| M4c6598081 | 4 | 6598081 | [A/G] |
| M4c7153998 | 4 | 7153998 | [T/C] |
| M4c7468959 | 4 | 7468959 | [T/C] |
| M4c7616857 | 4 | 7616857 | [A/G] |
| M4c8205873 | 4 | 8205873 | [A/G] |
| M4c8899489 | 4 | 8899489 | [T/C] |
| M4c8957570 | 4 | 8957570 | [A/G] |
| M4c9485353 | 4 | 9485353 | [T/G] |
| M4c9752147 | 4 | 9752147 | [T/C] |
| M4c10032486 | 4 | 10032486 | [A/G] |
| M4c10596800 | 4 | 10596800 | [A/C] |
| M4c10697004 | 4 | 10697004 | [T/C] |
| M4c11264343 | 4 | 11264343 | [T/C] |
| M4c11389631 | 4 | 11389631 | [T/C] |
| M4c12298423 | 4 | 12298423 | [A/G] |
| M4c12443948 | 4 | 12443948 | [T/C] |
| M4c13705066 | 4 | 13705066 | [A/C] |
| M4c13863477 | 4 | 13863477 | [A/G] |
| M4c14112724 | 4 | 14112724 | [T/C] |
| M4c14818099 | 4 | 14818099 | [T/C] |
| M4c15299308 | 4 | 15299308 | [A/G] |
| M4c15690002 | 4 | 15690002 | [A/C] |
| M4c15938143 | 4 | 15938143 | [A/G] |
| M4c16370526 | 4 | 16370526 | [A/C] |
| M4c16786235 | 4 | 16786235 | [T/C] |
| M4c16851599 | 4 | 16851599 | [A/G] |
| M4c17489280 | 4 | 17489280 | [T/C] |
| M4c17983623 | 4 | 17983623 | [A/G] |
| M4c18491450 | 4 | 18491450 | [A/C] |
| M4c18590044 | 4 | 18590044 | [A/G] |
| M4c18918644 | 4 | 18918644 | [A/G] |
| M4c19330083 | 4 | 19330083 | [A/G] |
| M4c19859745 | 4 | 19859745 | [A/C] |
| M4c20054320 | 4 | 20054320 | [T/G] |
| M4c20739902 | 4 | 20739902 | [A/G] |
| M4c21138645 | 4 | 21138645 | [T/C] |
| M4c21430542 | 4 | 21430542 | [A/G] |
| M4c22142029 | 4 | 22142029 | [A/C] |
| M4c22552800 | 4 | 22552800 | [T/C] |
| M4c22939399 | 4 | 22939399 | [T/C] |
| M4c23190139 | 4 | 23190139 | [A/G] |
| M4c23524571 | 4 | 23524571 | [A/C] |
| M4c23949335 | 4 | 23949335 | [T/C] |
| M4c24186328 | 4 | 24186328 | [T/C] |
| M4c24614720 | 4 | 24614720 | [A/C] |
| M4c24764627 | 4 | 24764627 | [T/G] |
| M4c25399284 | 4 | 25399284 | [A/G] |
| M4c25659672 | 4 | 25659672 | [A/G] |
| M4c25797161 | 4 | 25797161 | [T/C] |
| M4c26285375 | 4 | 26285375 | [A/G] |
| M4c26497470 | 4 | 26497470 | [A/G] |
| M4c26821163 | 4 | 26821163 | [A/G] |
| M4c27455130 | 4 | 27455130 | [T/C] |
| M4c27752635 | 4 | 27752635 | [A/G] |
| M4c28026867 | 4 | 28026867 | [A/G] |
| M4c28329566 | 4 | 28329566 | [A/G] |
| M4c28832601 | 4 | 28832601 | [T/C] |
| M4c28981066 | 4 | 28981066 | [T/C] |
| M4c29335471 | 4 | 29335471 | [T/C] |
| M4c29561724 | 4 | 29561724 | [A/G] |
| M4c29935575 | 4 | 29935575 | [T/C] |
| M4c30473321 | 4 | 30473321 | [T/C] |
| M4c30767214 | 4 | 30767214 | [T/C] |
| M4c31062922 | 4 | 31062922 | [A/G] |
| M4c31576107 | 4 | 31576107 | [T/C] |
| M4c31611196 | 4 | 31611196 | [T/C] |
| M4c32250331 | 4 | 32250331 | [T/C] |
| M4c32497909 | 4 | 32497909 | [A/G] |
| M4c32810639 | 4 | 32810639 | [T/C] |
| M4c32810884 | 4 | 32810884 | [T/C] |
| M4c33223696 | 4 | 33223696 | [A/C] |
| M4c33437905 | 4 | 33437905 | [A/G] |
| M4c33820331 | 4 | 33820331 | [T/C] |
| M4c34018579 | 4 | 34018579 | [A/G] |
| M4c34474146 | 4 | 34474146 | [A/C] |
| M4c34901943 | 4 | 34901943 | [A/G] |
| M4c35213690 | 4 | 35213690 | [T/C] |
| M4c35576322 | 4 | 35576322 | [T/G] |
| M4c36029293 | 4 | 36029293 | [A/C] |
| M4c36310096 | 4 | 36310096 | [T/C] |
| M4c36881660 | 4 | 36881660 | [T/C] |
| M4c37571615 | 4 | 37571615 | [A/G] |
| M4c38023367 | 4 | 38023367 | [A/G] |
| M4c38230105 | 4 | 38230105 | [T/C] |
| M4c38571629 | 4 | 38571629 | [A/G] |
| M4c38939330 | 4 | 38939330 | [T/G] |
| M4c39332038 | 4 | 39332038 | [A/G] |
| M4c39794414 | 4 | 39794414 | [A/C] |
| M4c39973556 | 4 | 39973556 | [T/C] |
| M4c40384877 | 4 | 40384877 | [A/G] |
| M4c40522307 | 4 | 40522307 | [A/G] |
| M4c41839666 | 4 | 41839666 | [T/C] |
| M4c41957588 | 4 | 41957588 | [T/C] |
| M4c42366405 | 4 | 42366405 | [T/C] |
| M4c43057373 | 4 | 43057373 | [T/C] |
| M4c44182524 | 4 | 44182524 | [A/G] |
| M4c44582257 | 4 | 44582257 | [T/G] |
| M4c44958493 | 4 | 44958493 | [A/G] |
| M4c45263272 | 4 | 45263272 | [A/G] |
| M4c46168821 | 4 | 46168821 | [A/G] |
| M4c46423649 | 4 | 46423649 | [A/G] |
| M4c46971188 | 4 | 46971188 | [A/G] |
| M4c47199091 | 4 | 47199091 | [A/C] |
| M4c47592757 | 4 | 47592757 | [A/G] |
| M4c48156367 | 4 | 48156367 | [T/C] |
| M4c48658967 | 4 | 48658967 | [A/G] |
| M4c49084167 | 4 | 49084167 | [A/G] |
| M4c49270998 | 4 | 49270998 | [T/G] |
| M4c49444626 | 4 | 49444626 | [A/G] |
| M4c49918126 | 4 | 49918126 | [A/C] |
| M4c50156422 | 4 | 50156422 | [A/G] |
| M4c50775770 | 4 | 50775770 | [T/C] |
| M4c51281012 | 4 | 51281012 | [A/G] |
| M4c51565598 | 4 | 51565598 | [T/C] |
| M4c52157276 | 4 | 52157276 | [T/C] |
| M4c52861388 | 4 | 52861388 | [T/C] |
| M4c53079877 | 4 | 53079877 | [A/G] |
| M4c53445466 | 4 | 53445466 | [T/C] |
| M4c53678226 | 4 | 53678226 | [A/C] |
| M4c54423639 | 4 | 54423639 | [A/G] |
| M4c54918663 | 4 | 54918663 | [T/G] |
| M4c55198916 | 4 | 55198916 | [T/C] |
| M4c55756415 | 4 | 55756415 | [A/G] |
| M4c56152339 | 4 | 56152339 | [A/G] |
| M4c56312634 | 4 | 56312634 | [A/G] |
| M4c56860537 | 4 | 56860537 | [T/C] |
| M4c57159304 | 4 | 57159304 | [A/G] |
| M4c57374005 | 4 | 57374005 | [A/G] |
| M4c58031648 | 4 | 58031648 | [T/G] |
| M4c58610280 | 4 | 58610280 | [A/G] |
| M4c58954964 | 4 | 58954964 | [T/G] |
| M4c59669053 | 4 | 59669053 | [T/C] |
| M4c60023545 | 4 | 60023545 | [A/G] |
| M4c60357510 | 4 | 60357510 | [A/G] |
| M4c60810636 | 4 | 60810636 | [T/C] |
| M4c61338936 | 4 | 61338936 | [A/G] |
| M4c61989721 | 4 | 61989721 | [T/C] |
| M4c62353542 | 4 | 62353542 | [T/C] |
| M4c62564815 | 4 | 62564815 | [A/C] |
| M4c62863368 | 4 | 62863368 | [T/C] |
| M4c63420154 | 4 | 63420154 | [A/G] |
| M4c63824015 | 4 | 63824015 | [A/C] |
| M4c63912388 | 4 | 63912388 | [A/G] |
| M4c65016950 | 4 | 65016950 | [T/G] |
| M4c65262117 | 4 | 65262117 | [A/C] |
| M4c65959860 | 4 | 65959860 | [T/C] |
| M4c66286010 | 4 | 66286010 | [T/C] |
| M4c66729820 | 4 | 66729820 | [A/G] |
| M4c67071852 | 4 | 67071852 | [T/C] |
| M4c67343049 | 4 | 67343049 | [T/C] |
| M4c67663701 | 4 | 67663701 | [T/C] |
| M4c68154594 | 4 | 68154594 | [A/C] |
| M4c68648729 | 4 | 68648729 | [T/G] |
| M4c68748698 | 4 | 68748698 | [A/G] |
| M4c69339398 | 4 | 69339398 | [A/G] |
| M4c69341509 | 4 | 69341509 | [A/G] |
| M4c69842966 | 4 | 69842966 | [T/G] |
| M4c70353667 | 4 | 70353667 | [A/C] |
| M4c70842760 | 4 | 70842760 | [T/G] |
| M4c71536171 | 4 | 71536171 | [T/G] |
| M4c72573426 | 4 | 72573426 | [T/C] |
| M4c72846656 | 4 | 72846656 | [A/C] |
| M4c73369365 | 4 | 73369365 | [T/C] |
| M4c73605553 | 4 | 73605553 | [T/C] |
| M4c73840497 | 4 | 73840497 | [T/C] |
| M4c74921287 | 4 | 74921287 | [T/G] |
| M4c75230995 | 4 | 75230995 | [A/G] |
| M4c75633216 | 4 | 75633216 | [T/C] |
| M4c76333109 | 4 | 76333109 | [A/G] |
| M4c76779637 | 4 | 76779637 | [T/C] |
| M4c77207732 | 4 | 77207732 | [A/G] |
| M4c77395684 | 4 | 77395684 | [A/G] |
| M4c77608730 | 4 | 77608730 | [A/G] |
| M4c78260008 | 4 | 78260008 | [A/C] |
| M4c78304321 | 4 | 78304321 | [A/G] |
| M4c78613158 | 4 | 78613158 | [A/G] |
| M4c79589466 | 4 | 79589466 | [T/C] |
| M4c79841743 | 4 | 79841743 | [A/G] |
| M4c80074977 | 4 | 80074977 | [T/C] |
| M4c80537071 | 4 | 80537071 | [A/G] |
| M4c80737878 | 4 | 80737878 | [T/C] |
| M4c81251263 | 4 | 81251263 | [T/C] |
| M4c81606118 | 4 | 81606118 | [T/C] |
| M4c81989038 | 4 | 81989038 | [T/C] |
| M4c82151350 | 4 | 82151350 | [A/G] |
| M4c82643985 | 4 | 82643985 | [A/C] |
| M4c82776341 | 4 | 82776341 | [A/C] |
| M4c83198379 | 4 | 83198379 | [T/G] |
| M4c83506535 | 4 | 83506535 | [A/G] |
| M4c84049925 | 4 | 84049925 | [T/C] |
| M4c84442707 | 4 | 84442707 | [A/G] |
| M4c85023428 | 4 | 85023428 | [A/G] |
| M4c85446580 | 4 | 85446580 | [T/C] |
| M4c86066726 | 4 | 86066726 | [T/C] |
| M4c86382390 | 4 | 86382390 | [A/G] |
| M4c86501714 | 4 | 86501714 | [A/G] |
| M4c87039084 | 4 | 87039084 | [T/C] |
| M4c88133635 | 4 | 88133635 | [A/G] |
| M4c88291632 | 4 | 88291632 | [T/C] |
| M4c88769673 | 4 | 88769673 | [A/G] |
| M4c89452667 | 4 | 89452667 | [A/G] |
| M4c90198969 | 4 | 90198969 | [T/G] |
| M4c90396315 | 4 | 90396315 | [T/G] |
| M4c90841246 | 4 | 90841246 | [A/C] |
| M4c91027567 | 4 | 91027567 | [T/G] |
| M4c91425309 | 4 | 91425309 | [T/C] |
| M4c91977602 | 4 | 91977602 | [T/G] |
| M4c92093985 | 4 | 92093985 | [A/G] |
| M4c92451350 | 4 | 92451350 | [T/G] |
| M4c93180999 | 4 | 93180999 | [T/C] |
| M4c93374137 | 4 | 93374137 | [A/C] |
| M4c93709974 | 4 | 93709974 | [A/C] |
| M4c94102812 | 4 | 94102812 | [A/G] |
| M4c94488137 | 4 | 94488137 | [T/C] |
| M4c94937630 | 4 | 94937630 | [T/G] |
| M4c95778880 | 4 | 95778880 | [A/C] |
| M4c96309068 | 4 | 96309068 | [A/G] |
| M4c96541882 | 4 | 96541882 | [T/G] |
| M4c96790899 | 4 | 96790899 | [T/C] |
| M4c97848206 | 4 | 97848206 | [T/G] |
| M4c98840448 | 4 | 98840448 | [T/C] |
| M4c99189019 | 4 | 99189019 | [T/C] |
| M4c99402352 | 4 | 99402352 | [A/G] |
| M4c99699311 | 4 | 99699311 | [T/C] |
| M4c99928068 | 4 | 99928068 | [A/G] |
| M4c102569226 | 4 | 102569226 | [T/C] |
| M4c102774645 | 4 | 102774645 | [A/G] |
| M4c103146524 | 4 | 103146524 | [T/C] |
| M4c103545904 | 4 | 103545904 | [T/C] |
| M4c103779995 | 4 | 103779995 | [A/C] |
| M4c105352686 | 4 | 105352686 | [A/C] |
| M4c106529368 | 4 | 106529368 | [T/C] |
| M4c107130219 | 4 | 107130219 | [A/G] |
| M4c107492957 | 4 | 107492957 | [T/C] |
| M4c108080202 | 4 | 108080202 | [A/C] |
| M4c108324922 | 4 | 108324922 | [T/C] |
| M4c108575686 | 4 | 108575686 | [T/C] |
| M4c109185296 | 4 | 109185296 | [A/G] |
| M4c109609541 | 4 | 109609541 | [T/C] |
| M4c109951513 | 4 | 109951513 | [T/C] |
| M4c110450359 | 4 | 110450359 | [T/C] |
| M4c110525140 | 4 | 110525140 | [T/C] |
| M4c110910404 | 4 | 110910404 | [A/C] |
| M4c112022254 | 4 | 112022254 | [A/G] |
| M4c112526064 | 4 | 112526064 | [A/G] |
| M4c112814933 | 4 | 112814933 | [T/C] |
| M4c113283936 | 4 | 113283936 | [A/G] |
| M4c113854877 | 4 | 113854877 | [T/G] |
| M4c114273102 | 4 | 114273102 | [T/C] |
| M4c114330551 | 4 | 114330551 | [T/C] |
| M4c114853641 | 4 | 114853641 | [A/C] |
| M4c115239690 | 4 | 115239690 | [T/G] |
| M4c116000669 | 4 | 116000669 | [A/G] |
| M4c116470417 | 4 | 116470417 | [T/C] |
| M4c116866144 | 4 | 116866144 | [T/C] |
| M4c117148026 | 4 | 117148026 | [T/C] |
| M4c117780928 | 4 | 117780928 | [T/C] |
| M4c118396333 | 4 | 118396333 | [T/G] |
| M4c118412279 | 4 | 118412279 | [A/G] |
| M4c119083136 | 4 | 119083136 | [A/G] |
| M4c119471020 | 4 | 119471020 | [A/G] |
| M4c119829680 | 4 | 119829680 | [T/C] |
| M4c120397735 | 4 | 120397735 | [T/C] |
| M4c120684912 | 4 | 120684912 | [A/G] |
| M4c121167803 | 4 | 121167803 | [A/G] |
| M4c121810047 | 4 | 121810047 | [A/C] |
| M4c121968145 | 4 | 121968145 | [A/C] |
| M4c122231472 | 4 | 122231472 | [A/G] |
| M4c122678410 | 4 | 122678410 | [T/C] |
| M4c122877057 | 4 | 122877057 | [T/C] |
| M4c123332893 | 4 | 123332893 | [T/G] |
| M4c123668118 | 4 | 123668118 | [A/C] |
| M4c123980616 | 4 | 123980616 | [A/G] |
| M4c124477994 | 4 | 124477994 | [A/C] |
| M4c124636531 | 4 | 124636531 | [A/G] |
| M4c125496232 | 4 | 125496232 | [T/C] |
| M4c125634387 | 4 | 125634387 | [A/G] |
| M4c125981314 | 4 | 125981314 | [A/G] |
| M4c126537846 | 4 | 126537846 | [T/C] |
| M4c127203026 | 4 | 127203026 | [A/G] |
| M4c127454955 | 4 | 127454955 | [A/C] |
| M4c127696697 | 4 | 127696697 | [T/C] |
| M4c128593749 | 4 | 128593749 | [T/C] |
| M4c129373391 | 4 | 129373391 | [T/G] |
| M4c129618964 | 4 | 129618964 | [T/C] |
| M4c129817779 | 4 | 129817779 | [T/C] |
| M4c130280780 | 4 | 130280780 | [T/C] |
| M4c130619206 | 4 | 130619206 | [A/G] |
| M4c130908269 | 4 | 130908269 | [A/C] |
| M4c131177323 | 4 | 131177323 | [A/G] |
| M4c131764913 | 4 | 131764913 | [A/G] |
| M4c131938293 | 4 | 131938293 | [A/G] |
| M4c132212425 | 4 | 132212425 | [A/G] |
| M4c132760125 | 4 | 132760125 | [T/G] |
| M4c133106147 | 4 | 133106147 | [A/G] |
| M4c133253688 | 4 | 133253688 | [A/C] |
| M4c133662066 | 4 | 133662066 | [T/G] |
| M4c134625724 | 4 | 134625724 | [A/G] |
| M4c134998171 | 4 | 134998171 | [T/C] |
| M4c135311224 | 4 | 135311224 | [T/C] |
| M4c135651148 | 4 | 135651148 | [T/G] |
| M4c135909493 | 4 | 135909493 | [A/C] |
| M4c136517418 | 4 | 136517418 | [A/C] |
| M4c136668729 | 4 | 136668729 | [A/G] |
| M4c137593228 | 4 | 137593228 | [T/C] |
| M4c137658506 | 4 | 137658506 | [T/C] |
| M4c138413782 | 4 | 138413782 | [T/C] |
| M4c138917570 | 4 | 138917570 | [A/G] |
| M4c139230576 | 4 | 139230576 | [T/C] |
| M4c139441669 | 4 | 139441669 | [A/G] |
| M4c139988435 | 4 | 139988435 | [A/C] |
| M4c140349277 | 4 | 140349277 | [T/C] |
| M4c140641171 | 4 | 140641171 | [A/G] |
| M4c140790186 | 4 | 140790186 | [A/G] |
| M4c141270569 | 4 | 141270569 | [A/C] |
| M4c141527614 | 4 | 141527614 | [T/C] |
| M4c142163752 | 4 | 142163752 | [A/G] |
| M4c142704259 | 4 | 142704259 | [T/C] |
| M4c143087803 | 4 | 143087803 | [A/G] |
| M4c143292648 | 4 | 143292648 | [A/G] |
| M4c143716879 | 4 | 143716879 | [T/C] |
| M4c144043799 | 4 | 144043799 | [T/G] |
| M4c144283581 | 4 | 144283581 | [T/C] |
| M4c144727138 | 4 | 144727138 | [T/C] |
| M4c144840683 | 4 | 144840683 | [T/C] |
| M4c145369695 | 4 | 145369695 | [A/G] |
| M4c145615210 | 4 | 145615210 | [A/G] |
| M4c145922186 | 4 | 145922186 | [T/C] |
| M4c146286855 | 4 | 146286855 | [A/C] |
| M4c146706994 | 4 | 146706994 | [T/G] |
| M4c147219276 | 4 | 147219276 | [T/C] |
| M4c147394010 | 4 | 147394010 | [T/C] |
| M4c147746862 | 4 | 147746862 | [T/G] |
| M4c148231987 | 4 | 148231987 | [A/G] |
| M4c148399278 | 4 | 148399278 | [A/G] |
| M4c148602572 | 4 | 148602572 | [A/G] |
| M4c149061163 | 4 | 149061163 | [A/G] |
| M4c149286755 | 4 | 149286755 | [T/C] |
| M4c149834538 | 4 | 149834538 | [A/G] |
| M4c150115461 | 4 | 150115461 | [A/G] |
| M4c150576283 | 4 | 150576283 | [A/G] |
| M4c150890615 | 4 | 150890615 | [T/G] |
| M4c151144213 | 4 | 151144213 | [T/C] |
| M4c151701676 | 4 | 151701676 | [T/C] |
| M4c152071890 | 4 | 152071890 | [T/C] |
| M4c152398781 | 4 | 152398781 | [T/G] |
| M4c152789653 | 4 | 152789653 | [A/G] |
| M4c153632836 | 4 | 153632836 | [A/G] |
| M4c154047191 | 4 | 154047191 | [A/G] |
| M4c154167076 | 4 | 154167076 | [A/G] |
| M4c154625042 | 4 | 154625042 | [A/G] |
| M4c155008677 | 4 | 155008677 | [T/C] |
| M4c155381136 | 4 | 155381136 | [T/G] |
| M4c155735446 | 4 | 155735446 | [A/C] |
| M4c155827731 | 4 | 155827731 | [A/G] |
| M4c156192322 | 4 | 156192322 | [A/G] |
| M4c156638203 | 4 | 156638203 | [A/C] |
| M4c156996314 | 4 | 156996314 | [T/C] |
| M4c157494238 | 4 | 157494238 | [A/G] |
| M4c157616561 | 4 | 157616561 | [A/G] |
| M4c157879556 | 4 | 157879556 | [A/G] |
| M4c158496981 | 4 | 158496981 | [A/G] |
| M4c158559601 | 4 | 158559601 | [T/C] |
| M4c159074160 | 4 | 159074160 | [A/C] |
| M4c159755184 | 4 | 159755184 | [T/C] |
| M4c160122392 | 4 | 160122392 | [A/G] |
| M4c160569662 | 4 | 160569662 | [T/C] |
| M4c160934436 | 4 | 160934436 | [A/G] |
| M4c161077119 | 4 | 161077119 | [T/C] |
| M4c161375594 | 4 | 161375594 | [A/G] |
| M4c161953926 | 4 | 161953926 | [T/C] |
| M4c162267661 | 4 | 162267661 | [T/C] |
| M4c162563344 | 4 | 162563344 | [T/C] |
| M4c162907836 | 4 | 162907836 | [T/C] |
| M4c163644563 | 4 | 163644563 | [A/G] |
| M4c163843729 | 4 | 163843729 | [T/C] |
| M4c164257949 | 4 | 164257949 | [A/G] |
| M4c165637692 | 4 | 165637692 | [A/G] |
| M4c167076270 | 4 | 167076270 | [A/G] |
| M4c167183055 | 4 | 167183055 | [A/G] |
| M4c167761991 | 4 | 167761991 | [A/G] |
| M4c168338401 | 4 | 168338401 | [A/G] |
| M4c168837697 | 4 | 168837697 | [T/C] |
| M4c168987417 | 4 | 168987417 | [A/C] |
| M4c169210027 | 4 | 169210027 | [T/G] |
| M4c169803020 | 4 | 169803020 | [T/C] |
| M4c170518569 | 4 | 170518569 | [A/G] |
| M4c170791705 | 4 | 170791705 | [A/C] |
| M4c171126206 | 4 | 171126206 | [T/C] |
| M4c171448191 | 4 | 171448191 | [A/G] |
| M4c171633713 | 4 | 171633713 | [T/C] |
| M4c172089690 | 4 | 172089690 | [A/G] |
| M4c172355999 | 4 | 172355999 | [A/G] |
| M4c172834970 | 4 | 172834970 | [T/G] |
| M4c172994023 | 4 | 172994023 | [T/C] |
| M4c173627639 | 4 | 173627639 | [A/G] |
| M4c173827108 | 4 | 173827108 | [T/C] |
| M4c173997010 | 4 | 173997010 | [T/G] |
| M4c174595892 | 4 | 174595892 | [A/C] |
| M4c174718739 | 4 | 174718739 | [A/G] |
| M4c175272679 | 4 | 175272679 | [A/G] |
| M4c175383245 | 4 | 175383245 | [T/C] |
| M4c175729040 | 4 | 175729040 | [T/C] |
| M4c176260031 | 4 | 176260031 | [A/G] |
| M4c176531277 | 4 | 176531277 | [A/C] |
| M4c176858872 | 4 | 176858872 | [A/G] |
| M4c177345782 | 4 | 177345782 | [T/C] |
| M4c177649049 | 4 | 177649049 | [A/G] |
| M4c178043945 | 4 | 178043945 | [A/C] |
| M4c178137657 | 4 | 178137657 | [T/G] |
| M4c178500153 | 4 | 178500153 | [T/C] |
| M4c179796874 | 4 | 179796874 | [T/C] |
| M4c180053538 | 4 | 180053538 | [T/G] |
| M4c180236183 | 4 | 180236183 | [A/C] |
| M4c181061502 | 4 | 181061502 | [T/C] |
| M4c181726003 | 4 | 181726003 | [A/G] |
| M4c181963756 | 4 | 181963756 | [A/C] |
| M4c182298138 | 4 | 182298138 | [T/C] |
| M4c182639128 | 4 | 182639128 | [A/G] |
| M4c182946612 | 4 | 182946612 | [T/C] |
| M4c183398899 | 4 | 183398899 | [T/C] |
| M4c183701178 | 4 | 183701178 | [A/G] |
| M4c184548766 | 4 | 184548766 | [T/C] |
| M4c185729631 | 4 | 185729631 | [A/G] |
| M4c186274282 | 4 | 186274282 | [A/G] |
| M4c186484390 | 4 | 186484390 | [A/G] |
| M4c186684167 | 4 | 186684167 | [T/G] |
| M4c187278435 | 4 | 187278435 | [A/G] |
| M4c187594876 | 4 | 187594876 | [A/C] |
| M4c187992567 | 4 | 187992567 | [T/C] |
| M4c188314565 | 4 | 188314565 | [T/G] |
| M4c188703595 | 4 | 188703595 | [T/C] |
| M4c189041979 | 4 | 189041979 | [A/G] |
| M4c189586484 | 4 | 189586484 | [A/G] |
| M4c189814797 | 4 | 189814797 | [A/G] |
| M4c190372771 | 4 | 190372771 | [A/G] |
| M4c190611189 | 4 | 190611189 | [A/G] |
| M4c191111480 | 4 | 191111480 | [A/G] |
| M4c191357841 | 4 | 191357841 | [T/G] |
| M4c191754995 | 4 | 191754995 | [A/G] |
| M4c191955175 | 4 | 191955175 | [T/C] |
| M4c192257611 | 4 | 192257611 | [A/G] |
| M4c192795925 | 4 | 192795925 | [T/G] |
| M4c193155631 | 4 | 193155631 | [A/G] |
| M4c193226797 | 4 | 193226797 | [A/G] |
| M4c193766453 | 4 | 193766453 | [A/G] |
| M4c194113383 | 4 | 194113383 | [A/G] |
| M4c194344037 | 4 | 194344037 | [A/G] |
| M4c194845932 | 4 | 194845932 | [T/C] |
| M4c194978215 | 4 | 194978215 | [T/C] |
| M4c195537146 | 4 | 195537146 | [A/C] |
| M4c195784278 | 4 | 195784278 | [A/G] |
| M4c196242566 | 4 | 196242566 | [A/G] |
| M4c196515170 | 4 | 196515170 | [A/G] |
| M4c196928187 | 4 | 196928187 | [T/C] |
| M4c197152132 | 4 | 197152132 | [A/G] |
| M4c197365493 | 4 | 197365493 | [A/G] |
| M4c197710757 | 4 | 197710757 | [T/C] |
| M4c198278681 | 4 | 198278681 | [A/G] |
| M4c198868480 | 4 | 198868480 | [A/G] |
| M4c199076543 | 4 | 199076543 | [A/C] |
| M4c199542386 | 4 | 199542386 | [T/C] |
| M4c199785143 | 4 | 199785143 | [T/C] |
| M4c200368158 | 4 | 200368158 | [T/G] |
| M4c200703228 | 4 | 200703228 | [T/C] |
| M4c200846517 | 4 | 200846517 | [T/C] |
| M4c201110345 | 4 | 201110345 | [T/C] |
| M4c201462099 | 4 | 201462099 | [T/C] |
| M4c202073021 | 4 | 202073021 | [T/C] |
| M4c202223852 | 4 | 202223852 | [T/C] |
| M4c202518235 | 4 | 202518235 | [T/C] |
| M4c203536847 | 4 | 203536847 | [A/C] |
| M4c204011502 | 4 | 204011502 | [T/C] |
| M4c204339561 | 4 | 204339561 | [A/G] |
| M4c205202566 | 4 | 205202566 | [T/C] |
| M4c205307401 | 4 | 205307401 | [T/C] |
| M4c205554992 | 4 | 205554992 | [T/G] |
| M4c205977547 | 4 | 205977547 | [A/G] |
| M4c206668246 | 4 | 206668246 | [A/G] |
| M4c207565516 | 4 | 207565516 | [T/G] |
| M4c207858290 | 4 | 207858290 | [A/G] |
| M4c208402180 | 4 | 208402180 | [T/C] |
| M4c208848050 | 4 | 208848050 | [A/G] |
| M4c209229971 | 4 | 209229971 | [T/C] |
| M4c209470107 | 4 | 209470107 | [A/G] |
| M4c210243373 | 4 | 210243373 | [A/G] |
| M4c210536729 | 4 | 210536729 | [T/C] |
| M4c210804647 | 4 | 210804647 | [T/C] |
| M4c211197113 | 4 | 211197113 | [T/G] |
| M4c211594187 | 4 | 211594187 | [T/G] |
| M4c212042175 | 4 | 212042175 | [A/G] |
| M4c212142654 | 4 | 212142654 | [A/C] |
| M4c212748213 | 4 | 212748213 | [A/G] |
| M4c213037838 | 4 | 213037838 | [T/C] |
| M4c213347252 | 4 | 213347252 | [A/G] |
| M4c213769875 | 4 | 213769875 | [T/C] |
| M4c213961911 | 4 | 213961911 | [T/C] |
| M4c214377114 | 4 | 214377114 | [A/C] |
| M4c214545491 | 4 | 214545491 | [T/C] |
| M4c214905226 | 4 | 214905226 | [T/C] |
| M4c215405822 | 4 | 215405822 | [T/C] |
| M4c215715252 | 4 | 215715252 | [T/C] |
| M4c216371298 | 4 | 216371298 | [T/C] |
| M4c216812264 | 4 | 216812264 | [T/G] |
| M4c217023967 | 4 | 217023967 | [T/C] |
| M4c217369219 | 4 | 217369219 | [T/C] |
| M4c217689633 | 4 | 217689633 | [A/G] |
| M4c218145242 | 4 | 218145242 | [A/G] |
| M4c218330848 | 4 | 218330848 | [A/G] |
| M4c218668283 | 4 | 218668283 | [A/G] |
| M4c218945417 | 4 | 218945417 | [A/G] |
| M4c219603060 | 4 | 219603060 | [T/C] |
| M4c219707135 | 4 | 219707135 | [T/C] |
| M4c220089780 | 4 | 220089780 | [A/G] |
| M4c220610845 | 4 | 220610845 | [A/G] |
| M4c220655870 | 4 | 220655870 | [A/G] |
| M4c221187413 | 4 | 221187413 | [T/C] |
| M4c221901458 | 4 | 221901458 | [A/C] |
| M4c222230537 | 4 | 222230537 | [T/C] |
| M4c222687636 | 4 | 222687636 | [A/G] |
| M4c222826423 | 4 | 222826423 | [A/G] |
| M4c223216502 | 4 | 223216502 | [A/G] |
| M4c224168825 | 4 | 224168825 | [T/C] |
| M4c224725208 | 4 | 224725208 | [A/C] |
| M4c225070551 | 4 | 225070551 | [T/C] |
| M4c225259260 | 4 | 225259260 | [T/C] |
| M4c225992853 | 4 | 225992853 | [T/C] |
| M4c226459824 | 4 | 226459824 | [T/C] |
| M4c226719865 | 4 | 226719865 | [T/G] |
| M4c226942005 | 4 | 226942005 | [A/G] |
| M4c227213625 | 4 | 227213625 | [T/C] |
| M4c227595478 | 4 | 227595478 | [T/G] |
| M4c228448733 | 4 | 228448733 | [T/C] |
| M4c228553512 | 4 | 228553512 | [A/G] |
| M4c229036587 | 4 | 229036587 | [T/C] |
| M4c229344081 | 4 | 229344081 | [A/G] |
| M4c229738659 | 4 | 229738659 | [T/C] |
| M4c229968007 | 4 | 229968007 | [T/C] |
| M4c230798523 | 4 | 230798523 | [T/C] |
| M4c231209119 | 4 | 231209119 | [A/G] |
| M4c231965143 | 4 | 231965143 | [T/G] |
| M4c232129810 | 4 | 232129810 | [A/G] |
| M4c232849770 | 4 | 232849770 | [T/G] |
| M4c233328520 | 4 | 233328520 | [T/C] |
| M4c233443169 | 4 | 233443169 | [T/C] |
| M4c234358410 | 4 | 234358410 | [A/G] |
| M4c234626827 | 4 | 234626827 | [T/C] |
| M4c234726780 | 4 | 234726780 | [T/C] |
| M4c235228646 | 4 | 235228646 | [T/C] |
| M4c235606766 | 4 | 235606766 | [T/C] |
| M4c236083383 | 4 | 236083383 | [T/C] |
| M4c236083488 | 4 | 236083488 | [T/C] |
| M4c236109268 | 4 | 236109268 | [A/C] |
| M4c236175035 | 4 | 236175035 | [T/C] |
| M4c236474011 | 4 | 236474011 | [T/C] |
| M4c236475263 | 4 | 236475263 | [A/G] |
| M4c236881687 | 4 | 236881687 | [T/C] |
| M4c237041732 | 4 | 237041732 | [A/C] |
| M4c237391476 | 4 | 237391476 | [T/C] |
| M4c237777656 | 4 | 237777656 | [A/G] |
| M4c238059564 | 4 | 238059564 | [A/G] |
| M4c238298929 | 4 | 238298929 | [T/C] |
| M4c238374755 | 4 | 238374755 | [A/G] |
| M4c238758660 | 4 | 238758660 | [T/C] |
| M4c238763301 | 4 | 238763301 | [T/C] |
| M4c238963086 | 4 | 238963086 | [T/C] |
| M4c239407776 | 4 | 239407776 | [A/G] |
| M4c239499500 | 4 | 239499500 | [T/C] |
| M4c239650401 | 4 | 239650401 | [T/C] |
| M4c239690281 | 4 | 239690281 | [T/C] |
| M4c239896686 | 4 | 239896686 | [A/G] |
| M4c239932920 | 4 | 239932920 | [A/G] |
| M4c240465668 | 4 | 240465668 | [T/C] |
| M4c241079088 | 4 | 241079088 | [A/G] |
| M5c21571 | 5 | 21571 | [T/G] |
| M5c109496 | 5 | 109496 | [T/C] |
| M5c648565 | 5 | 648565 | [A/G] |
| M5c1026204 | 5 | 1026204 | [T/C] |
| M5c1037491 | 5 | 1037491 | [T/C] |
| M5c1339120 | 5 | 1339120 | [T/C] |
| M5c1676152 | 5 | 1676152 | [T/C] |
| M5c1820513 | 5 | 1820513 | [T/C] |
| M5c1943512 | 5 | 1943512 | [A/G] |
| M5c2379314 | 5 | 2379314 | [A/G] |
| M5c3343433 | 5 | 3343433 | [A/G] |
| M5c3351902 | 5 | 3351902 | [A/C] |
| M5c3667742 | 5 | 3667742 | [A/C] |
| M5c3779537 | 5 | 3779537 | [A/G] |
| M5c3947168 | 5 | 3947168 | [A/G] |
| M5c4014973 | 5 | 4014973 | [A/C] |
| M5c4314833 | 5 | 4314833 | [A/G] |
| M5c4684888 | 5 | 4684888 | [T/G] |
| M5c5217683 | 5 | 5217683 | [A/G] |
| M5c5433999 | 5 | 5433999 | [A/G] |
| M5c5902928 | 5 | 5902928 | [T/C] |
| M5c6105668 | 5 | 6105668 | [T/C] |
| M5c6526809 | 5 | 6526809 | [A/G] |
| M5c6676863 | 5 | 6676863 | [A/G] |
| M5c7279237 | 5 | 7279237 | [T/C] |
| M5c7458288 | 5 | 7458288 | [A/C] |
| M5c7857008 | 5 | 7857008 | [A/G] |
| M5c7996619 | 5 | 7996619 | [T/C] |
| M5c8513808 | 5 | 8513808 | [T/C] |
| M5c9246196 | 5 | 9246196 | [T/C] |
| M5c9635641 | 5 | 9635641 | [T/C] |
| M5c9812698 | 5 | 9812698 | [T/C] |
| M5c10089609 | 5 | 10089609 | [T/C] |
| M5c10680338 | 5 | 10680338 | [T/C] |
| M5c11065782 | 5 | 11065782 | [T/C] |
| M5c11392159 | 5 | 11392159 | [A/G] |
| M5c11691957 | 5 | 11691957 | [A/G] |
| M5c11952463 | 5 | 11952463 | [T/C] |
| M5c12219760 | 5 | 12219760 | [T/G] |
| M5c12580816 | 5 | 12580816 | [A/G] |
| M5c13076268 | 5 | 13076268 | [T/C] |
| M5c13390979 | 5 | 13390979 | [T/C] |
| M5c14072437 | 5 | 14072437 | [A/G] |
| M5c14472662 | 5 | 14472662 | [A/G] |
| M5c14752884 | 5 | 14752884 | [T/C] |
| M5c15193425 | 5 | 15193425 | [T/C] |
| M5c15544163 | 5 | 15544163 | [T/C] |
| M5c16217349 | 5 | 16217349 | [T/C] |
| M5c16304145 | 5 | 16304145 | [A/G] |
| M5c16615900 | 5 | 16615900 | [A/G] |
| M5c17229164 | 5 | 17229164 | [T/C] |
| M5c17274308 | 5 | 17274308 | [A/G] |
| M5c17780354 | 5 | 17780354 | [T/C] |
| M5c18281911 | 5 | 18281911 | [A/G] |
| M5c18540913 | 5 | 18540913 | [A/C] |
| M5c18753657 | 5 | 18753657 | [A/C] |
| M5c19133269 | 5 | 19133269 | [A/G] |
| M5c19457917 | 5 | 19457917 | [T/C] |
| M5c19892656 | 5 | 19892656 | [T/C] |
| M5c20200526 | 5 | 20200526 | [A/C] |
| M5c20603556 | 5 | 20603556 | [T/G] |
| M5c21343930 | 5 | 21343930 | [A/G] |
| M5c21583728 | 5 | 21583728 | [A/G] |
| M5c21893051 | 5 | 21893051 | [T/G] |
| M5c22357442 | 5 | 22357442 | [A/C] |
| M5c22628835 | 5 | 22628835 | [A/G] |
| M5c22761912 | 5 | 22761912 | [A/C] |
| M5c23337478 | 5 | 23337478 | [A/G] |
| M5c23573052 | 5 | 23573052 | [T/C] |
| M5c23874761 | 5 | 23874761 | [A/G] |
| M5c24162998 | 5 | 24162998 | [A/G] |
| M5c24617066 | 5 | 24617066 | [A/G] |
| M5c24950778 | 5 | 24950778 | [A/G] |
| M5c25436298 | 5 | 25436298 | [A/C] |
| M5c25616536 | 5 | 25616536 | [T/C] |
| M5c25915223 | 5 | 25915223 | [A/G] |
| M5c26314431 | 5 | 26314431 | [A/C] |
| M5c26564832 | 5 | 26564832 | [T/C] |
| M5c27055853 | 5 | 27055853 | [A/G] |
| M5c27394919 | 5 | 27394919 | [T/C] |
| M5c28196541 | 5 | 28196541 | [A/C] |
| M5c28335285 | 5 | 28335285 | [T/C] |
| M5c28680529 | 5 | 28680529 | [A/G] |
| M5c29177528 | 5 | 29177528 | [A/G] |
| M5c29324719 | 5 | 29324719 | [A/C] |
| M5c30624238 | 5 | 30624238 | [A/C] |
| M5c30759525 | 5 | 30759525 | [T/C] |
| M5c31132422 | 5 | 31132422 | [A/G] |
| M5c31444326 | 5 | 31444326 | [A/G] |
| M5c31857877 | 5 | 31857877 | [A/C] |
| M5c32353554 | 5 | 32353554 | [T/G] |
| M5c32848179 | 5 | 32848179 | [T/C] |
| M5c33287477 | 5 | 33287477 | [A/G] |
| M5c33968310 | 5 | 33968310 | [T/C] |
| M5c34309649 | 5 | 34309649 | [A/C] |
| M5c34965757 | 5 | 34965757 | [T/C] |
| M5c35300837 | 5 | 35300837 | [T/G] |
| M5c35725329 | 5 | 35725329 | [A/G] |
| M5c36065951 | 5 | 36065951 | [T/C] |
| M5c36284079 | 5 | 36284079 | [T/C] |
| M5c36764755 | 5 | 36764755 | [A/G] |
| M5c36932219 | 5 | 36932219 | [A/C] |
| M5c37256600 | 5 | 37256600 | [A/G] |
| M5c37950336 | 5 | 37950336 | [T/C] |
| M5c38808163 | 5 | 38808163 | [A/C] |
| M5c38992475 | 5 | 38992475 | [A/C] |
| M5c39403337 | 5 | 39403337 | [T/G] |
| M5c40338219 | 5 | 40338219 | [A/G] |
| M5c41141026 | 5 | 41141026 | [T/C] |
| M5c41553149 | 5 | 41553149 | [T/C] |
| M5c41637623 | 5 | 41637623 | [T/C] |
| M5c42231141 | 5 | 42231141 | [A/G] |
| M5c42461827 | 5 | 42461827 | [T/G] |
| M5c42846688 | 5 | 42846688 | [T/C] |
| M5c43154752 | 5 | 43154752 | [T/C] |
| M5c43420028 | 5 | 43420028 | [T/C] |
| M5c43738299 | 5 | 43738299 | [T/C] |
| M5c44153116 | 5 | 44153116 | [A/G] |
| M5c44405670 | 5 | 44405670 | [A/G] |
| M5c44718026 | 5 | 44718026 | [A/G] |
| M5c45333476 | 5 | 45333476 | [T/G] |
| M5c45440557 | 5 | 45440557 | [T/G] |
| M5c45906399 | 5 | 45906399 | [A/G] |
| M5c46526507 | 5 | 46526507 | [T/C] |
| M5c46766139 | 5 | 46766139 | [A/G] |
| M5c47205509 | 5 | 47205509 | [A/G] |
| M5c47579424 | 5 | 47579424 | [A/G] |
| M5c48629612 | 5 | 48629612 | [T/C] |
| M5c48881156 | 5 | 48881156 | [T/C] |
| M5c49826835 | 5 | 49826835 | [T/G] |
| M5c49899493 | 5 | 49899493 | [T/C] |
| M5c50451330 | 5 | 50451330 | [T/C] |
| M5c50552660 | 5 | 50552660 | [T/C] |
| M5c51180978 | 5 | 51180978 | [A/G] |
| M5c51359824 | 5 | 51359824 | [A/G] |
| M5c51692724 | 5 | 51692724 | [T/G] |
| M5c52235564 | 5 | 52235564 | [T/C] |
| M5c52273107 | 5 | 52273107 | [A/G] |
| M5c52745592 | 5 | 52745592 | [A/G] |
| M5c53173773 | 5 | 53173773 | [A/C] |
| M5c53396223 | 5 | 53396223 | [T/C] |
| M5c53907070 | 5 | 53907070 | [T/C] |
| M5c53978258 | 5 | 53978258 | [A/G] |
| M5c54612121 | 5 | 54612121 | [A/G] |
| M5c54992598 | 5 | 54992598 | [A/G] |
| M5c55107944 | 5 | 55107944 | [A/G] |
| M5c55560497 | 5 | 55560497 | [A/G] |
| M5c55788582 | 5 | 55788582 | [A/C] |
| M5c56049019 | 5 | 56049019 | [T/C] |
| M5c56372336 | 5 | 56372336 | [A/C] |
| M5c56782978 | 5 | 56782978 | [T/C] |
| M5c57193958 | 5 | 57193958 | [A/G] |
| M5c57434191 | 5 | 57434191 | [A/G] |
| M5c58005376 | 5 | 58005376 | [T/C] |
| M5c58206441 | 5 | 58206441 | [T/C] |
| M5c58607239 | 5 | 58607239 | [A/C] |
| M5c59271333 | 5 | 59271333 | [T/G] |
| M5c59467709 | 5 | 59467709 | [T/G] |
| M5c59805514 | 5 | 59805514 | [A/G] |
| M5c60282107 | 5 | 60282107 | [T/G] |
| M5c60596122 | 5 | 60596122 | [T/C] |
| M5c60996345 | 5 | 60996345 | [T/G] |
| M5c61353927 | 5 | 61353927 | [T/C] |
| M5c61697453 | 5 | 61697453 | [A/G] |
| M5c62113519 | 5 | 62113519 | [A/G] |
| M5c62206519 | 5 | 62206519 | [T/G] |
| M5c62756250 | 5 | 62756250 | [T/C] |
| M5c62921231 | 5 | 62921231 | [A/G] |
| M5c63567727 | 5 | 63567727 | [T/C] |
| M5c64253744 | 5 | 64253744 | [T/C] |
| M5c64439893 | 5 | 64439893 | [A/C] |
| M5c64681353 | 5 | 64681353 | [A/G] |
| M5c65225599 | 5 | 65225599 | [T/G] |
| M5c65936096 | 5 | 65936096 | [A/G] |
| M5c66114088 | 5 | 66114088 | [A/G] |
| M5c66423575 | 5 | 66423575 | [A/C] |
| M5c66836999 | 5 | 66836999 | [T/C] |
| M5c67017135 | 5 | 67017135 | [A/G] |
| M5c67634390 | 5 | 67634390 | [T/G] |
| M5c68021466 | 5 | 68021466 | [A/G] |
| M5c68195023 | 5 | 68195023 | [A/G] |
| M5c68648543 | 5 | 68648543 | [A/G] |
| M5c69303336 | 5 | 69303336 | [A/G] |
| M5c69434227 | 5 | 69434227 | [A/G] |
| M5c70083510 | 5 | 70083510 | [T/C] |
| M5c70150938 | 5 | 70150938 | [A/G] |
| M5c70773319 | 5 | 70773319 | [T/C] |
| M5c70783551 | 5 | 70783551 | [A/G] |
| M5c71382625 | 5 | 71382625 | [T/C] |
| M5c71687090 | 5 | 71687090 | [A/G] |
| M5c71847319 | 5 | 71847319 | [T/C] |
| M5c72408807 | 5 | 72408807 | [T/G] |
| M5c72691069 | 5 | 72691069 | [T/C] |
| M5c73050070 | 5 | 73050070 | [T/C] |
| M5c73327507 | 5 | 73327507 | [A/G] |
| M5c74043747 | 5 | 74043747 | [A/G] |
| M5c74293916 | 5 | 74293916 | [A/G] |
| M5c74657261 | 5 | 74657261 | [T/C] |
| M5c74917986 | 5 | 74917986 | [T/C] |
| M5c76210391 | 5 | 76210391 | [A/G] |
| M5c76377907 | 5 | 76377907 | [T/C] |
| M5c76634818 | 5 | 76634818 | [T/C] |
| M5c77269643 | 5 | 77269643 | [A/G] |
| M5c77447428 | 5 | 77447428 | [T/C] |
| M5c77758866 | 5 | 77758866 | [T/C] |
| M5c77998736 | 5 | 77998736 | [A/C] |
| M5c78381238 | 5 | 78381238 | [A/G] |
| M5c78779371 | 5 | 78779371 | [T/C] |
| M5c79157554 | 5 | 79157554 | [T/G] |
| M5c79679938 | 5 | 79679938 | [A/G] |
| M5c79857039 | 5 | 79857039 | [T/G] |
| M5c80389273 | 5 | 80389273 | [A/G] |
| M5c80828554 | 5 | 80828554 | [A/C] |
| M5c81080658 | 5 | 81080658 | [A/G] |
| M5c81455347 | 5 | 81455347 | [A/G] |
| M5c81857955 | 5 | 81857955 | [T/C] |
| M5c82429270 | 5 | 82429270 | [T/C] |
| M5c82554735 | 5 | 82554735 | [T/C] |
| M5c82954843 | 5 | 82954843 | [A/G] |
| M5c83277611 | 5 | 83277611 | [T/C] |
| M5c83559669 | 5 | 83559669 | [T/C] |
| M5c84088323 | 5 | 84088323 | [T/C] |
| M5c84840822 | 5 | 84840822 | [T/C] |
| M5c85028803 | 5 | 85028803 | [T/C] |
| M5c85235256 | 5 | 85235256 | [T/G] |
| M5c85740416 | 5 | 85740416 | [T/C] |
| M5c86191202 | 5 | 86191202 | [T/C] |
| M5c86260683 | 5 | 86260683 | [T/C] |
| M5c87524551 | 5 | 87524551 | [T/C] |
| M5c88046716 | 5 | 88046716 | [T/G] |
| M5c88334288 | 5 | 88334288 | [T/G] |
| M5c88752267 | 5 | 88752267 | [A/G] |
| M5c89001954 | 5 | 89001954 | [T/C] |
| M5c89723990 | 5 | 89723990 | [T/C] |
| M5c90102945 | 5 | 90102945 | [A/G] |
| M5c90341921 | 5 | 90341921 | [T/C] |
| M5c90843221 | 5 | 90843221 | [A/G] |
| M5c91364696 | 5 | 91364696 | [T/C] |
| M5c91813640 | 5 | 91813640 | [T/C] |
| M5c92369931 | 5 | 92369931 | [T/C] |
| M5c92681108 | 5 | 92681108 | [T/G] |
| M5c92806494 | 5 | 92806494 | [A/C] |
| M5c93171319 | 5 | 93171319 | [A/C] |
| M5c93581737 | 5 | 93581737 | [T/C] |
| M5c93837922 | 5 | 93837922 | [A/G] |
| M5c94531264 | 5 | 94531264 | [T/C] |
| M5c95001993 | 5 | 95001993 | [A/G] |
| M5c95218908 | 5 | 95218908 | [A/G] |
| M5c95632723 | 5 | 95632723 | [A/G] |
| M5c95901619 | 5 | 95901619 | [T/C] |
| M5c96304708 | 5 | 96304708 | [A/C] |
| M5c96534748 | 5 | 96534748 | [T/C] |
| M5c97174313 | 5 | 97174313 | [A/G] |
| M5c97501218 | 5 | 97501218 | [A/G] |
| M5c97707020 | 5 | 97707020 | [A/C] |
| M5c98274899 | 5 | 98274899 | [A/G] |
| M5c98573021 | 5 | 98573021 | [T/G] |
| M5c98993592 | 5 | 98993592 | [T/C] |
| M5c99500938 | 5 | 99500938 | [A/G] |
| M5c99928353 | 5 | 99928353 | [T/C] |
| M5c100011317 | 5 | 100011317 | [A/G] |
| M5c100447943 | 5 | 100447943 | [A/G] |
| M5c100699643 | 5 | 100699643 | [A/C] |
| M5c101299328 | 5 | 101299328 | [T/C] |
| M5c101635631 | 5 | 101635631 | [T/C] |
| M5c101736934 | 5 | 101736934 | [A/G] |
| M5c102269529 | 5 | 102269529 | [T/G] |
| M5c102870701 | 5 | 102870701 | [T/C] |
| M5c103564983 | 5 | 103564983 | [A/G] |
| M5c104215351 | 5 | 104215351 | [T/G] |
| M5c104747103 | 5 | 104747103 | [T/C] |
| M5c105041835 | 5 | 105041835 | [A/G] |
| M5c105243023 | 5 | 105243023 | [T/C] |
| M5c105766225 | 5 | 105766225 | [T/C] |
| M5c106101033 | 5 | 106101033 | [A/G] |
| M5c106229435 | 5 | 106229435 | [A/G] |
| M5c106634996 | 5 | 106634996 | [A/G] |
| M5c107144944 | 5 | 107144944 | [A/G] |
| M5c107241109 | 5 | 107241109 | [A/G] |
| M5c108030000 | 5 | 108030000 | [A/G] |
| M5c108224333 | 5 | 108224333 | [T/G] |
| M5c108597071 | 5 | 108597071 | [A/G] |
| M5c109326025 | 5 | 109326025 | [A/G] |
| M5c110013238 | 5 | 110013238 | [T/C] |
| M5c110462571 | 5 | 110462571 | [T/G] |
| M5c111262742 | 5 | 111262742 | [A/G] |
| M5c111345399 | 5 | 111345399 | [A/G] |
| M5c111663268 | 5 | 111663268 | [T/G] |
| M5c111998749 | 5 | 111998749 | [T/G] |
| M5c112610779 | 5 | 112610779 | [T/C] |
| M5c112926050 | 5 | 112926050 | [T/C] |
| M5c113198825 | 5 | 113198825 | [T/C] |
| M5c113588264 | 5 | 113588264 | [A/G] |
| M5c113749846 | 5 | 113749846 | [A/G] |
| M5c114090549 | 5 | 114090549 | [A/G] |
| M5c114767932 | 5 | 114767932 | [T/C] |
| M5c115148597 | 5 | 115148597 | [T/C] |
| M5c115419960 | 5 | 115419960 | [T/G] |
| M5c115730699 | 5 | 115730699 | [A/G] |
| M5c116919466 | 5 | 116919466 | [T/C] |
| M5c117260293 | 5 | 117260293 | [T/C] |
| M5c117989871 | 5 | 117989871 | [T/G] |
| M5c118148897 | 5 | 118148897 | [A/G] |
| M5c118673668 | 5 | 118673668 | [T/C] |
| M5c119482522 | 5 | 119482522 | [T/G] |
| M5c120349531 | 5 | 120349531 | [A/G] |
| M5c120576859 | 5 | 120576859 | [T/C] |
| M5c120984518 | 5 | 120984518 | [T/C] |
| M5c121236161 | 5 | 121236161 | [T/C] |
| M5c121770980 | 5 | 121770980 | [A/G] |
| M5c122161810 | 5 | 122161810 | [T/C] |
| M5c122481712 | 5 | 122481712 | [A/G] |
| M5c122642167 | 5 | 122642167 | [T/C] |
| M5c123255590 | 5 | 123255590 | [T/C] |
| M5c123335984 | 5 | 123335984 | [T/C] |
| M5c123894151 | 5 | 123894151 | [A/G] |
| M5c124417259 | 5 | 124417259 | [T/C] |
| M5c125493704 | 5 | 125493704 | [T/G] |
| M5c125824701 | 5 | 125824701 | [T/G] |
| M5c126026957 | 5 | 126026957 | [A/C] |
| M5c126820317 | 5 | 126820317 | [T/G] |
| M5c127357195 | 5 | 127357195 | [T/C] |
| M5c127605006 | 5 | 127605006 | [T/C] |
| M5c127809699 | 5 | 127809699 | [T/C] |
| M5c128140018 | 5 | 128140018 | [T/C] |
| M5c129548685 | 5 | 129548685 | [T/C] |
| M5c129989514 | 5 | 129989514 | [A/C] |
| M5c130359477 | 5 | 130359477 | [A/G] |
| M5c130658802 | 5 | 130658802 | [A/G] |
| M5c130956665 | 5 | 130956665 | [A/G] |
| M5c131734265 | 5 | 131734265 | [A/G] |
| M5c132172405 | 5 | 132172405 | [T/C] |
| M5c132314121 | 5 | 132314121 | [A/G] |
| M5c132712280 | 5 | 132712280 | [T/C] |
| M5c133333397 | 5 | 133333397 | [T/G] |
| M5c133719410 | 5 | 133719410 | [A/G] |
| M5c134205590 | 5 | 134205590 | [A/G] |
| M5c135278708 | 5 | 135278708 | [A/G] |
| M5c135302345 | 5 | 135302345 | [A/G] |
| M5c136200206 | 5 | 136200206 | [T/C] |
| M5c136567200 | 5 | 136567200 | [A/G] |
| M5c136958422 | 5 | 136958422 | [T/C] |
| M5c137152292 | 5 | 137152292 | [T/G] |
| M5c137852051 | 5 | 137852051 | [T/C] |
| M5c138152098 | 5 | 138152098 | [A/C] |
| M5c138506106 | 5 | 138506106 | [T/C] |
| M5c138875797 | 5 | 138875797 | [T/C] |
| M5c139626107 | 5 | 139626107 | [T/G] |
| M5c139767416 | 5 | 139767416 | [T/C] |
| M5c140093536 | 5 | 140093536 | [A/G] |
| M5c140764385 | 5 | 140764385 | [T/C] |
| M5c140781215 | 5 | 140781215 | [T/G] |
| M5c141168244 | 5 | 141168244 | [A/G] |
| M5c141578105 | 5 | 141578105 | [T/C] |
| M5c142137152 | 5 | 142137152 | [A/G] |
| M5c142181287 | 5 | 142181287 | [T/C] |
| M5c142711421 | 5 | 142711421 | [A/G] |
| M5c143163766 | 5 | 143163766 | [T/C] |
| M5c143185948 | 5 | 143185948 | [A/G] |
| M5c143669303 | 5 | 143669303 | [T/C] |
| M5c144114260 | 5 | 144114260 | [A/G] |
| M5c144522939 | 5 | 144522939 | [T/C] |
| M5c144713442 | 5 | 144713442 | [T/G] |
| M5c145215521 | 5 | 145215521 | [T/C] |
| M5c145524567 | 5 | 145524567 | [T/C] |
| M5c145714748 | 5 | 145714748 | [T/C] |
| M5c146389114 | 5 | 146389114 | [A/G] |
| M5c146887877 | 5 | 146887877 | [A/G] |
| M5c147089634 | 5 | 147089634 | [A/G] |
| M5c147984675 | 5 | 147984675 | [A/C] |
| M5c148396668 | 5 | 148396668 | [A/G] |
| M5c148882939 | 5 | 148882939 | [T/C] |
| M5c149760886 | 5 | 149760886 | [T/G] |
| M5c150089295 | 5 | 150089295 | [T/C] |
| M5c150957280 | 5 | 150957280 | [T/C] |
| M5c151394123 | 5 | 151394123 | [A/C] |
| M5c151713082 | 5 | 151713082 | [A/G] |
| M5c152431894 | 5 | 152431894 | [T/C] |
| M5c152744906 | 5 | 152744906 | [A/C] |
| M5c152919972 | 5 | 152919972 | [A/G] |
| M5c153340280 | 5 | 153340280 | [T/C] |
| M5c153548392 | 5 | 153548392 | [A/C] |
| M5c153945373 | 5 | 153945373 | [T/C] |
| M5c154351003 | 5 | 154351003 | [T/G] |
| M5c154808379 | 5 | 154808379 | [T/C] |
| M5c154987745 | 5 | 154987745 | [T/C] |
| M5c155243568 | 5 | 155243568 | [A/G] |
| M5c155531042 | 5 | 155531042 | [T/C] |
| M5c155959609 | 5 | 155959609 | [T/C] |
| M5c156444413 | 5 | 156444413 | [T/C] |
| M5c156593296 | 5 | 156593296 | [A/G] |
| M5c157202556 | 5 | 157202556 | [A/C] |
| M5c157497742 | 5 | 157497742 | [A/G] |
| M5c157650228 | 5 | 157650228 | [A/G] |
| M5c158194281 | 5 | 158194281 | [A/G] |
| M5c158544956 | 5 | 158544956 | [A/G] |
| M5c158866334 | 5 | 158866334 | [T/C] |
| M5c159201483 | 5 | 159201483 | [A/G] |
| M5c159538128 | 5 | 159538128 | [A/G] |
| M5c159912768 | 5 | 159912768 | [T/C] |
| M5c160458741 | 5 | 160458741 | [T/G] |
| M5c160717435 | 5 | 160717435 | [A/G] |
| M5c161192912 | 5 | 161192912 | [A/G] |
| M5c161395832 | 5 | 161395832 | [A/C] |
| M5c162008360 | 5 | 162008360 | [A/G] |
| M5c162059218 | 5 | 162059218 | [T/C] |
| M5c162515826 | 5 | 162515826 | [T/C] |
| M5c162895542 | 5 | 162895542 | [A/G] |
| M5c163412997 | 5 | 163412997 | [T/C] |
| M5c163611610 | 5 | 163611610 | [T/C] |
| M5c163940933 | 5 | 163940933 | [T/G] |
| M5c164366006 | 5 | 164366006 | [T/G] |
| M5c165320067 | 5 | 165320067 | [T/G] |
| M5c165653908 | 5 | 165653908 | [T/C] |
| M5c165976711 | 5 | 165976711 | [T/G] |
| M5c166411388 | 5 | 166411388 | [T/C] |
| M5c166572455 | 5 | 166572455 | [T/G] |
| M5c166856517 | 5 | 166856517 | [A/G] |
| M5c167250191 | 5 | 167250191 | [A/C] |
| M5c167585648 | 5 | 167585648 | [A/G] |
| M5c167969385 | 5 | 167969385 | [A/G] |
| M5c168224869 | 5 | 168224869 | [T/C] |
| M5c168886091 | 5 | 168886091 | [T/G] |
| M5c169232780 | 5 | 169232780 | [A/G] |
| M5c169457935 | 5 | 169457935 | [A/G] |
| M5c169668222 | 5 | 169668222 | [A/G] |
| M5c170065271 | 5 | 170065271 | [A/G] |
| M5c170401968 | 5 | 170401968 | [T/C] |
| M5c171276541 | 5 | 171276541 | [T/G] |
| M5c171366701 | 5 | 171366701 | [T/C] |
| M5c172290454 | 5 | 172290454 | [T/G] |
| M5c172475369 | 5 | 172475369 | [A/G] |
| M5c173693292 | 5 | 173693292 | [A/G] |
| M5c173801671 | 5 | 173801671 | [T/C] |
| M5c174366544 | 5 | 174366544 | [A/C] |
| M5c174411617 | 5 | 174411617 | [T/C] |
| M5c175181814 | 5 | 175181814 | [T/C] |
| M5c175616382 | 5 | 175616382 | [T/C] |
| M5c176038263 | 5 | 176038263 | [A/G] |
| M5c176215669 | 5 | 176215669 | [A/C] |
| M5c176591486 | 5 | 176591486 | [A/C] |
| M5c177450522 | 5 | 177450522 | [A/C] |
| M5c178116758 | 5 | 178116758 | [T/G] |
| M5c178828178 | 5 | 178828178 | [T/C] |
| M5c179153205 | 5 | 179153205 | [T/C] |
| M5c179392567 | 5 | 179392567 | [T/C] |
| M5c179543968 | 5 | 179543968 | [A/C] |
| M5c179928953 | 5 | 179928953 | [T/C] |
| M5c180370727 | 5 | 180370727 | [A/G] |
| M5c180737118 | 5 | 180737118 | [T/C] |
| M5c181003365 | 5 | 181003365 | [T/C] |
| M5c181588954 | 5 | 181588954 | [T/C] |
| M5c182450672 | 5 | 182450672 | [A/G] |
| M5c183155294 | 5 | 183155294 | [T/G] |
| M5c183482161 | 5 | 183482161 | [A/G] |
| M5c183882582 | 5 | 183882582 | [T/C] |
| M5c184234139 | 5 | 184234139 | [A/C] |
| M5c184473381 | 5 | 184473381 | [T/G] |
| M5c184992394 | 5 | 184992394 | [T/C] |
| M5c185769538 | 5 | 185769538 | [T/C] |
| M5c186507410 | 5 | 186507410 | [A/G] |
| M5c187007654 | 5 | 187007654 | [A/G] |
| M5c187200246 | 5 | 187200246 | [A/G] |
| M5c187661445 | 5 | 187661445 | [A/G] |
| M5c188105179 | 5 | 188105179 | [T/C] |
| M5c188389404 | 5 | 188389404 | [T/C] |
| M5c188463898 | 5 | 188463898 | [T/C] |
| M5c189041919 | 5 | 189041919 | [T/G] |
| M5c189400564 | 5 | 189400564 | [A/G] |
| M5c189771809 | 5 | 189771809 | [A/G] |
| M5c189879873 | 5 | 189879873 | [A/G] |
| M5c190734054 | 5 | 190734054 | [T/C] |
| M5c191094406 | 5 | 191094406 | [A/G] |
| M5c191485086 | 5 | 191485086 | [T/G] |
| M5c191588655 | 5 | 191588655 | [T/C] |
| M5c191992245 | 5 | 191992245 | [T/C] |
| M5c192263164 | 5 | 192263164 | [A/G] |
| M5c192702837 | 5 | 192702837 | [T/C] |
| M5c193161213 | 5 | 193161213 | [T/C] |
| M5c193554706 | 5 | 193554706 | [A/C] |
| M5c193728445 | 5 | 193728445 | [T/C] |
| M5c193970016 | 5 | 193970016 | [T/C] |
| M5c194399064 | 5 | 194399064 | [A/G] |
| M5c194707930 | 5 | 194707930 | [A/G] |
| M5c195305780 | 5 | 195305780 | [A/G] |
| M5c195443613 | 5 | 195443613 | [T/C] |
| M5c196142601 | 5 | 196142601 | [T/C] |
| M5c196510823 | 5 | 196510823 | [T/C] |
| M5c196801925 | 5 | 196801925 | [T/C] |
| M5c198030693 | 5 | 198030693 | [T/C] |
| M5c198577649 | 5 | 198577649 | [A/C] |
| M5c198818482 | 5 | 198818482 | [A/G] |
| M5c199229239 | 5 | 199229239 | [A/G] |
| M5c199762148 | 5 | 199762148 | [T/C] |
| M5c200114137 | 5 | 200114137 | [A/C] |
| M5c200435300 | 5 | 200435300 | [A/G] |
| M5c200686830 | 5 | 200686830 | [T/G] |
| M5c200877874 | 5 | 200877874 | [T/G] |
| M5c201227667 | 5 | 201227667 | [T/C] |
| M5c201669846 | 5 | 201669846 | [A/G] |
| M5c202086146 | 5 | 202086146 | [T/C] |
| M5c202416360 | 5 | 202416360 | [T/C] |
| M5c202811864 | 5 | 202811864 | [T/C] |
| M5c203314299 | 5 | 203314299 | [T/C] |
| M5c203819976 | 5 | 203819976 | [T/C] |
| M5c203936934 | 5 | 203936934 | [T/C] |
| M5c204549411 | 5 | 204549411 | [A/G] |
| M5c204625103 | 5 | 204625103 | [T/C] |
| M5c205041167 | 5 | 205041167 | [T/G] |
| M5c205340963 | 5 | 205340963 | [A/G] |
| M5c205725779 | 5 | 205725779 | [A/G] |
| M5c206014632 | 5 | 206014632 | [A/G] |
| M5c206571801 | 5 | 206571801 | [A/G] |
| M5c206890351 | 5 | 206890351 | [T/C] |
| M5c207031234 | 5 | 207031234 | [T/C] |
| M5c207515369 | 5 | 207515369 | [T/C] |
| M5c207705154 | 5 | 207705154 | [T/C] |
| M5c208262001 | 5 | 208262001 | [T/C] |
| M5c208429010 | 5 | 208429010 | [T/C] |
| M5c208741702 | 5 | 208741702 | [T/C] |
| M5c209150290 | 5 | 209150290 | [T/C] |
| M5c209685412 | 5 | 209685412 | [A/G] |
| M5c209872309 | 5 | 209872309 | [A/G] |
| M5c210304858 | 5 | 210304858 | [A/G] |
| M5c210503431 | 5 | 210503431 | [T/C] |
| M5c210883864 | 5 | 210883864 | [T/C] |
| M5c211179931 | 5 | 211179931 | [T/G] |
| M5c212049063 | 5 | 212049063 | [T/C] |
| M5c212650775 | 5 | 212650775 | [A/G] |
| M5c213002141 | 5 | 213002141 | [A/C] |
| M5c213285549 | 5 | 213285549 | [T/G] |
| M5c213945845 | 5 | 213945845 | [T/C] |
| M5c214531411 | 5 | 214531411 | [T/C] |
| M5c214762037 | 5 | 214762037 | [T/C] |
| M5c215026856 | 5 | 215026856 | [T/C] |
| M5c215061412 | 5 | 215061412 | [T/C] |
| M5c215259706 | 5 | 215259706 | [A/G] |
| M5c215478547 | 5 | 215478547 | [T/C] |
| M5c215786179 | 5 | 215786179 | [T/G] |
| M5c215836310 | 5 | 215836310 | [A/G] |
| M5c215977124 | 5 | 215977124 | [A/C] |
| M5c215991421 | 5 | 215991421 | [T/G] |
| M5c216620742 | 5 | 216620742 | [T/G] |
| M5c217415206 | 5 | 217415206 | [T/C] |
| M5c217627742 | 5 | 217627742 | [T/C] |
| M6c372092 | 6 | 372092 | [A/G] |
| M6c662608 | 6 | 662608 | [A/G] |
| M6c798829 | 6 | 798829 | [T/C] |
| M6c1239818 | 6 | 1239818 | [A/G] |
| M6c1712762 | 6 | 1712762 | [T/C] |
| M6c2043933 | 6 | 2043933 | [T/C] |
| M6c2439634 | 6 | 2439634 | [A/G] |
| M6c2590531 | 6 | 2590531 | [T/G] |
| M6c3053714 | 6 | 3053714 | [T/C] |
| M6c3193962 | 6 | 3193962 | [A/G] |
| M6c3973841 | 6 | 3973841 | [T/C] |
| M6c4802108 | 6 | 4802108 | [A/G] |
| M6c5160521 | 6 | 5160521 | [A/G] |
| M6c5300547 | 6 | 5300547 | [T/C] |
| M6c5644062 | 6 | 5644062 | [A/G] |
| M6c6269443 | 6 | 6269443 | [A/C] |
| M6c6514321 | 6 | 6514321 | [A/C] |
| M6c6807559 | 6 | 6807559 | [A/G] |
| M6c7046840 | 6 | 7046840 | [A/C] |
| M6c7621828 | 6 | 7621828 | [A/G] |
| M6c7780961 | 6 | 7780961 | [A/G] |
| M6c8132560 | 6 | 8132560 | [A/G] |
| M6c8389526 | 6 | 8389526 | [T/C] |
| M6c9206066 | 6 | 9206066 | [A/G] |
| M6c9494025 | 6 | 9494025 | [T/C] |
| M6c9857455 | 6 | 9857455 | [T/G] |
| M6c10278673 | 6 | 10278673 | [T/C] |
| M6c10489931 | 6 | 10489931 | [A/C] |
| M6c10911577 | 6 | 10911577 | [A/C] |
| M6c11432668 | 6 | 11432668 | [A/C] |
| M6c12424091 | 6 | 12424091 | [A/C] |
| M6c12460471 | 6 | 12460471 | [T/G] |
| M6c12952313 | 6 | 12952313 | [A/G] |
| M6c13225721 | 6 | 13225721 | [T/C] |
| M6c13805402 | 6 | 13805402 | [A/G] |
| M6c13858772 | 6 | 13858772 | [A/G] |
| M6c14462004 | 6 | 14462004 | [T/C] |
| M6c14732417 | 6 | 14732417 | [A/G] |
| M6c14866452 | 6 | 14866452 | [A/G] |
| M6c15506977 | 6 | 15506977 | [T/C] |
| M6c15644406 | 6 | 15644406 | [A/C] |
| M6c16179092 | 6 | 16179092 | [T/C] |
| M6c16293036 | 6 | 16293036 | [A/G] |
| M6c16802079 | 6 | 16802079 | [T/C] |
| M6c17650064 | 6 | 17650064 | [T/C] |
| M6c17960951 | 6 | 17960951 | [T/G] |
| M6c18535896 | 6 | 18535896 | [T/G] |
| M6c18990291 | 6 | 18990291 | [A/C] |
| M6c19368529 | 6 | 19368529 | [A/G] |
| M6c19980743 | 6 | 19980743 | [T/C] |
| M6c20565051 | 6 | 20565051 | [A/C] |
| M6c21001223 | 6 | 21001223 | [T/C] |
| M6c21184851 | 6 | 21184851 | [A/C] |
| M6c21377346 | 6 | 21377346 | [T/G] |
| M6c22002697 | 6 | 22002697 | [A/G] |
| M6c22307104 | 6 | 22307104 | [T/C] |
| M6c22562090 | 6 | 22562090 | [A/C] |
| M6c22913319 | 6 | 22913319 | [A/G] |
| M6c23356731 | 6 | 23356731 | [T/C] |
| M6c23725628 | 6 | 23725628 | [T/C] |
| M6c23828204 | 6 | 23828204 | [A/G] |
| M6c24551637 | 6 | 24551637 | [A/G] |
| M6c25703710 | 6 | 25703710 | [A/G] |
| M6c26243119 | 6 | 26243119 | [A/G] |
| M6c26668014 | 6 | 26668014 | [T/C] |
| M6c27112567 | 6 | 27112567 | [A/G] |
| M6c27500967 | 6 | 27500967 | [T/C] |
| M6c27551710 | 6 | 27551710 | [A/G] |
| M6c28225073 | 6 | 28225073 | [A/G] |
| M6c28231426 | 6 | 28231426 | [T/C] |
| M6c28883653 | 6 | 28883653 | [T/C] |
| M6c28988697 | 6 | 28988697 | [A/G] |
| M6c29351068 | 6 | 29351068 | [A/G] |
| M6c29638814 | 6 | 29638814 | [A/G] |
| M6c30185766 | 6 | 30185766 | [T/G] |
| M6c30523505 | 6 | 30523505 | [A/G] |
| M6c30823963 | 6 | 30823963 | [A/G] |
| M6c31567780 | 6 | 31567780 | [T/C] |
| M6c31707820 | 6 | 31707820 | [A/G] |
| M6c32188244 | 6 | 32188244 | [A/C] |
| M6c32467045 | 6 | 32467045 | [T/C] |
| M6c32891604 | 6 | 32891604 | [A/G] |
| M6c33247550 | 6 | 33247550 | [A/G] |
| M6c33639195 | 6 | 33639195 | [T/C] |
| M6c33792358 | 6 | 33792358 | [T/C] |
| M6c34123987 | 6 | 34123987 | [T/C] |
| M6c34889179 | 6 | 34889179 | [T/C] |
| M6c35314668 | 6 | 35314668 | [A/G] |
| M6c35680813 | 6 | 35680813 | [T/C] |
| M6c35896073 | 6 | 35896073 | [T/G] |
| M6c36317995 | 6 | 36317995 | [A/G] |
| M6c36624353 | 6 | 36624353 | [A/G] |
| M6c36946692 | 6 | 36946692 | [A/G] |
| M6c37490733 | 6 | 37490733 | [T/G] |
| M6c37597071 | 6 | 37597071 | [T/C] |
| M6c38086280 | 6 | 38086280 | [T/C] |
| M6c38428663 | 6 | 38428663 | [T/C] |
| M6c38792386 | 6 | 38792386 | [A/C] |
| M6c39092546 | 6 | 39092546 | [A/G] |
| M6c39273159 | 6 | 39273159 | [A/G] |
| M6c39565437 | 6 | 39565437 | [A/G] |
| M6c39898281 | 6 | 39898281 | [A/C] |
| M6c40311394 | 6 | 40311394 | [T/C] |
| M6c40622939 | 6 | 40622939 | [A/G] |
| M6c41189663 | 6 | 41189663 | [T/G] |
| M6c41892257 | 6 | 41892257 | [T/C] |
| M6c42079904 | 6 | 42079904 | [T/C] |
| M6c42597121 | 6 | 42597121 | [A/G] |
| M6c42731679 | 6 | 42731679 | [T/G] |
| M6c44156217 | 6 | 44156217 | [A/G] |
| M6c44600228 | 6 | 44600228 | [A/G] |
| M6c44717137 | 6 | 44717137 | [T/C] |
| M6c45276471 | 6 | 45276471 | [A/G] |
| M6c45708709 | 6 | 45708709 | [T/G] |
| M6c45922975 | 6 | 45922975 | [A/C] |
| M6c46111110 | 6 | 46111110 | [A/G] |
| M6c46657140 | 6 | 46657140 | [A/C] |
| M6c46775297 | 6 | 46775297 | [T/C] |
| M6c48717105 | 6 | 48717105 | [T/C] |
| M6c48888792 | 6 | 48888792 | [A/G] |
| M6c49749058 | 6 | 49749058 | [T/C] |
| M6c49864140 | 6 | 49864140 | [A/G] |
| M6c50393470 | 6 | 50393470 | [A/G] |
| M6c50854527 | 6 | 50854527 | [A/G] |
| M6c51441234 | 6 | 51441234 | [T/G] |
| M6c51976914 | 6 | 51976914 | [A/G] |
| M6c52516710 | 6 | 52516710 | [T/C] |
| M6c52887498 | 6 | 52887498 | [A/G] |
| M6c52976625 | 6 | 52976625 | [T/C] |
| M6c53338189 | 6 | 53338189 | [A/G] |
| M6c53723914 | 6 | 53723914 | [T/G] |
| M6c54073707 | 6 | 54073707 | [A/G] |
| M6c54402835 | 6 | 54402835 | [T/G] |
| M6c54775883 | 6 | 54775883 | [A/G] |
| M6c55254281 | 6 | 55254281 | [T/G] |
| M6c55673214 | 6 | 55673214 | [A/G] |
| M6c55885011 | 6 | 55885011 | [T/C] |
| M6c56218680 | 6 | 56218680 | [A/G] |
| M6c56539412 | 6 | 56539412 | [A/C] |
| M6c57281858 | 6 | 57281858 | [A/C] |
| M6c57578862 | 6 | 57578862 | [T/C] |
| M6c57749203 | 6 | 57749203 | [A/G] |
| M6c58446775 | 6 | 58446775 | [T/G] |
| M6c59467790 | 6 | 59467790 | [T/C] |
| M6c59897388 | 6 | 59897388 | [A/G] |
| M6c60177832 | 6 | 60177832 | [A/G] |
| M6c60981497 | 6 | 60981497 | [T/G] |
| M6c61182730 | 6 | 61182730 | [T/C] |
| M6c61740963 | 6 | 61740963 | [A/G] |
| M6c62180974 | 6 | 62180974 | [T/C] |
| M6c62451254 | 6 | 62451254 | [T/C] |
| M6c62557439 | 6 | 62557439 | [A/G] |
| M6c63117686 | 6 | 63117686 | [A/G] |
| M6c63453596 | 6 | 63453596 | [T/C] |
| M6c63699585 | 6 | 63699585 | [A/G] |
| M6c63949629 | 6 | 63949629 | [A/G] |
| M6c64523360 | 6 | 64523360 | [A/G] |
| M6c65265768 | 6 | 65265768 | [A/G] |
| M6c65560961 | 6 | 65560961 | [A/C] |
| M6c65926072 | 6 | 65926072 | [T/G] |
| M6c66065370 | 6 | 66065370 | [T/C] |
| M6c66897824 | 6 | 66897824 | [T/C] |
| M6c67258537 | 6 | 67258537 | [T/G] |
| M6c67860223 | 6 | 67860223 | [A/G] |
| M6c68232782 | 6 | 68232782 | [T/G] |
| M6c68813220 | 6 | 68813220 | [T/C] |
| M6c69262160 | 6 | 69262160 | [A/C] |
| M6c69726072 | 6 | 69726072 | [A/G] |
| M6c69866995 | 6 | 69866995 | [T/C] |
| M6c70292528 | 6 | 70292528 | [A/G] |
| M6c71014292 | 6 | 71014292 | [A/G] |
| M6c71234825 | 6 | 71234825 | [A/G] |
| M6c71733640 | 6 | 71733640 | [A/G] |
| M6c71866928 | 6 | 71866928 | [T/C] |
| M6c72476476 | 6 | 72476476 | [A/G] |
| M6c72871051 | 6 | 72871051 | [A/G] |
| M6c73200772 | 6 | 73200772 | [A/G] |
| M6c73857855 | 6 | 73857855 | [A/C] |
| M6c74072474 | 6 | 74072474 | [T/C] |
| M6c74463114 | 6 | 74463114 | [T/C] |
| M6c74967604 | 6 | 74967604 | [T/G] |
| M6c75289437 | 6 | 75289437 | [A/G] |
| M6c75744975 | 6 | 75744975 | [A/G] |
| M6c76236112 | 6 | 76236112 | [A/G] |
| M6c76491915 | 6 | 76491915 | [A/G] |
| M6c76817241 | 6 | 76817241 | [A/C] |
| M6c77002452 | 6 | 77002452 | [T/C] |
| M6c77465624 | 6 | 77465624 | [A/G] |
| M6c77714029 | 6 | 77714029 | [A/G] |
| M6c78098938 | 6 | 78098938 | [T/G] |
| M6c78624201 | 6 | 78624201 | [T/C] |
| M6c78835605 | 6 | 78835605 | [A/G] |
| M6c79459531 | 6 | 79459531 | [A/G] |
| M6c80641056 | 6 | 80641056 | [A/G] |
| M6c80979748 | 6 | 80979748 | [A/G] |
| M6c81089214 | 6 | 81089214 | [A/G] |
| M6c81693352 | 6 | 81693352 | [A/G] |
| M6c81917585 | 6 | 81917585 | [A/C] |
| M6c82186516 | 6 | 82186516 | [A/G] |
| M6c82620288 | 6 | 82620288 | [A/C] |
| M6c82967363 | 6 | 82967363 | [T/C] |
| M6c83218783 | 6 | 83218783 | [A/G] |
| M6c83618154 | 6 | 83618154 | [A/G] |
| M6c83834987 | 6 | 83834987 | [A/G] |
| M6c84378989 | 6 | 84378989 | [A/G] |
| M6c84674762 | 6 | 84674762 | [A/G] |
| M6c85160399 | 6 | 85160399 | [A/C] |
| M6c85206564 | 6 | 85206564 | [T/G] |
| M6c85797329 | 6 | 85797329 | [A/G] |
| M6c86051055 | 6 | 86051055 | [T/C] |
| M6c86782890 | 6 | 86782890 | [T/C] |
| M6c87120700 | 6 | 87120700 | [T/C] |
| M6c87398930 | 6 | 87398930 | [A/G] |
| M6c87798893 | 6 | 87798893 | [A/G] |
| M6c88152214 | 6 | 88152214 | [T/G] |
| M6c88592932 | 6 | 88592932 | [T/C] |
| M6c88898370 | 6 | 88898370 | [A/G] |
| M6c88993367 | 6 | 88993367 | [T/C] |
| M6c89464926 | 6 | 89464926 | [A/G] |
| M6c89885787 | 6 | 89885787 | [T/G] |
| M6c90306459 | 6 | 90306459 | [A/G] |
| M6c90531590 | 6 | 90531590 | [A/C] |
| M6c90734666 | 6 | 90734666 | [A/G] |
| M6c91150330 | 6 | 91150330 | [T/G] |
| M6c91644334 | 6 | 91644334 | [T/C] |
| M6c91991460 | 6 | 91991460 | [A/C] |
| M6c92304502 | 6 | 92304502 | [A/G] |
| M6c92396681 | 6 | 92396681 | [A/C] |
| M6c93000683 | 6 | 93000683 | [T/G] |
| M6c93327800 | 6 | 93327800 | [T/C] |
| M6c93501111 | 6 | 93501111 | [A/G] |
| M6c93928107 | 6 | 93928107 | [A/G] |
| M6c94422847 | 6 | 94422847 | [T/C] |
| M6c94714266 | 6 | 94714266 | [T/C] |
| M6c94992494 | 6 | 94992494 | [A/C] |
| M6c95318771 | 6 | 95318771 | [A/G] |
| M6c95609789 | 6 | 95609789 | [A/C] |
| M6c95846377 | 6 | 95846377 | [A/C] |
| M6c96215014 | 6 | 96215014 | [A/G] |
| M6c96698265 | 6 | 96698265 | [T/C] |
| M6c96910766 | 6 | 96910766 | [A/G] |
| M6c97272935 | 6 | 97272935 | [A/G] |
| M6c97631149 | 6 | 97631149 | [A/G] |
| M6c98254432 | 6 | 98254432 | [A/G] |
| M6c98574753 | 6 | 98574753 | [T/C] |
| M6c99128125 | 6 | 99128125 | [T/C] |
| M6c99261906 | 6 | 99261906 | [T/C] |
| M6c99771241 | 6 | 99771241 | [A/C] |
| M6c99953711 | 6 | 99953711 | [A/G] |
| M6c100744568 | 6 | 100744568 | [T/C] |
| M6c101003156 | 6 | 101003156 | [T/C] |
| M6c102203771 | 6 | 102203771 | [T/C] |
| M6c102597765 | 6 | 102597765 | [A/G] |
| M6c102755092 | 6 | 102755092 | [A/G] |
| M6c103606785 | 6 | 103606785 | [T/G] |
| M6c103988928 | 6 | 103988928 | [A/G] |
| M6c104276011 | 6 | 104276011 | [T/C] |
| M6c104676914 | 6 | 104676914 | [A/G] |
| M6c104981653 | 6 | 104981653 | [A/G] |
| M6c105098420 | 6 | 105098420 | [T/C] |
| M6c105538436 | 6 | 105538436 | [T/C] |
| M6c106069077 | 6 | 106069077 | [T/C] |
| M6c106343142 | 6 | 106343142 | [T/C] |
| M6c106645093 | 6 | 106645093 | [A/C] |
| M6c107038517 | 6 | 107038517 | [A/G] |
| M6c107363338 | 6 | 107363338 | [T/C] |
| M6c107809678 | 6 | 107809678 | [A/G] |
| M6c108121379 | 6 | 108121379 | [A/C] |
| M6c108342025 | 6 | 108342025 | [A/G] |
| M6c109098023 | 6 | 109098023 | [A/G] |
| M6c109294235 | 6 | 109294235 | [A/G] |
| M6c109880360 | 6 | 109880360 | [T/C] |
| M6c110014458 | 6 | 110014458 | [T/C] |
| M6c110270082 | 6 | 110270082 | [A/G] |
| M6c111250500 | 6 | 111250500 | [A/G] |
| M6c111558772 | 6 | 111558772 | [A/G] |
| M6c111767615 | 6 | 111767615 | [A/G] |
| M6c111966409 | 6 | 111966409 | [A/G] |
| M6c112358553 | 6 | 112358553 | [T/C] |
| M6c113559728 | 6 | 113559728 | [T/C] |
| M6c113781674 | 6 | 113781674 | [A/C] |
| M6c114096906 | 6 | 114096906 | [T/C] |
| M6c114572757 | 6 | 114572757 | [A/G] |
| M6c114942421 | 6 | 114942421 | [A/G] |
| M6c115130607 | 6 | 115130607 | [A/G] |
| M6c115754148 | 6 | 115754148 | [T/C] |
| M6c116330584 | 6 | 116330584 | [T/G] |
| M6c116477790 | 6 | 116477790 | [A/G] |
| M6c117014189 | 6 | 117014189 | [T/C] |
| M6c117227295 | 6 | 117227295 | [T/C] |
| M6c117586086 | 6 | 117586086 | [A/G] |
| M6c117803575 | 6 | 117803575 | [T/C] |
| M6c118314115 | 6 | 118314115 | [A/G] |
| M6c118604688 | 6 | 118604688 | [T/C] |
| M6c118863220 | 6 | 118863220 | [T/G] |
| M6c119158886 | 6 | 119158886 | [T/C] |
| M6c119692070 | 6 | 119692070 | [A/G] |
| M6c119876370 | 6 | 119876370 | [T/C] |
| M6c120281601 | 6 | 120281601 | [A/G] |
| M6c120737800 | 6 | 120737800 | [A/C] |
| M6c120965170 | 6 | 120965170 | [A/G] |
| M6c121390584 | 6 | 121390584 | [T/C] |
| M6c121671402 | 6 | 121671402 | [A/G] |
| M6c121944679 | 6 | 121944679 | [A/G] |
| M6c122730103 | 6 | 122730103 | [A/G] |
| M6c123201565 | 6 | 123201565 | [A/G] |
| M6c123766950 | 6 | 123766950 | [A/G] |
| M6c124151732 | 6 | 124151732 | [T/C] |
| M6c124299981 | 6 | 124299981 | [T/G] |
| M6c124966467 | 6 | 124966467 | [T/C] |
| M6c125187777 | 6 | 125187777 | [A/G] |
| M6c125593444 | 6 | 125593444 | [T/G] |
| M6c125881396 | 6 | 125881396 | [T/C] |
| M6c126198400 | 6 | 126198400 | [A/G] |
| M6c126429346 | 6 | 126429346 | [T/C] |
| M6c126957402 | 6 | 126957402 | [A/G] |
| M6c128064689 | 6 | 128064689 | [T/C] |
| M6c128160736 | 6 | 128160736 | [T/G] |
| M6c128798059 | 6 | 128798059 | [T/C] |
| M6c129139474 | 6 | 129139474 | [T/C] |
| M6c129514090 | 6 | 129514090 | [A/G] |
| M6c129966634 | 6 | 129966634 | [T/C] |
| M6c130349418 | 6 | 130349418 | [A/G] |
| M6c130599671 | 6 | 130599671 | [T/G] |
| M6c130927669 | 6 | 130927669 | [A/C] |
| M6c131426353 | 6 | 131426353 | [T/C] |
| M6c132130395 | 6 | 132130395 | [T/C] |
| M6c132189856 | 6 | 132189856 | [T/C] |
| M6c133208338 | 6 | 133208338 | [T/G] |
| M6c133355261 | 6 | 133355261 | [A/C] |
| M6c133662143 | 6 | 133662143 | [T/G] |
| M6c134098356 | 6 | 134098356 | [T/C] |
| M6c134516552 | 6 | 134516552 | [T/C] |
| M6c135000336 | 6 | 135000336 | [T/C] |
| M6c135320518 | 6 | 135320518 | [A/G] |
| M6c135877240 | 6 | 135877240 | [T/C] |
| M6c136283202 | 6 | 136283202 | [T/C] |
| M6c136361394 | 6 | 136361394 | [T/C] |
| M6c136913745 | 6 | 136913745 | [T/G] |
| M6c137269300 | 6 | 137269300 | [A/G] |
| M6c137641298 | 6 | 137641298 | [T/C] |
| M6c138489488 | 6 | 138489488 | [A/G] |
| M6c138709055 | 6 | 138709055 | [A/G] |
| M6c139877385 | 6 | 139877385 | [T/C] |
| M6c140196875 | 6 | 140196875 | [A/C] |
| M6c140725788 | 6 | 140725788 | [A/G] |
| M6c141281114 | 6 | 141281114 | [A/C] |
| M6c141511381 | 6 | 141511381 | [A/G] |
| M6c143134652 | 6 | 143134652 | [T/C] |
| M6c143407253 | 6 | 143407253 | [A/G] |
| M6c143696195 | 6 | 143696195 | [T/C] |
| M6c143953856 | 6 | 143953856 | [T/C] |
| M6c144127409 | 6 | 144127409 | [A/C] |
| M6c144337293 | 6 | 144337293 | [T/C] |
| M6c145167033 | 6 | 145167033 | [T/G] |
| M6c145319662 | 6 | 145319662 | [A/C] |
| M6c145644151 | 6 | 145644151 | [A/G] |
| M6c145990751 | 6 | 145990751 | [T/C] |
| M6c146499659 | 6 | 146499659 | [A/G] |
| M6c146895157 | 6 | 146895157 | [T/C] |
| M6c147024676 | 6 | 147024676 | [T/G] |
| M6c147444344 | 6 | 147444344 | [T/C] |
| M6c147656847 | 6 | 147656847 | [T/G] |
| M6c148592727 | 6 | 148592727 | [T/C] |
| M6c148720269 | 6 | 148720269 | [T/C] |
| M6c149125656 | 6 | 149125656 | [A/G] |
| M6c149470209 | 6 | 149470209 | [A/G] |
| M6c150015512 | 6 | 150015512 | [A/C] |
| M6c150519316 | 6 | 150519316 | [T/C] |
| M6c150844304 | 6 | 150844304 | [T/C] |
| M6c151228549 | 6 | 151228549 | [T/C] |
| M6c151823581 | 6 | 151823581 | [T/G] |
| M6c152186634 | 6 | 152186634 | [T/C] |
| M6c152972908 | 6 | 152972908 | [A/C] |
| M6c153308161 | 6 | 153308161 | [A/G] |
| M6c153498090 | 6 | 153498090 | [T/G] |
| M6c154779907 | 6 | 154779907 | [T/C] |
| M6c155105540 | 6 | 155105540 | [T/C] |
| M6c155457919 | 6 | 155457919 | [A/G] |
| M6c155647497 | 6 | 155647497 | [T/C] |
| M6c155987552 | 6 | 155987552 | [A/G] |
| M6c156253128 | 6 | 156253128 | [A/G] |
| M6c156594452 | 6 | 156594452 | [T/C] |
| M6c157027050 | 6 | 157027050 | [T/C] |
| M6c157567743 | 6 | 157567743 | [A/G] |
| M6c157890854 | 6 | 157890854 | [T/G] |
| M6c157991729 | 6 | 157991729 | [A/G] |
| M6c158652589 | 6 | 158652589 | [T/C] |
| M6c159260067 | 6 | 159260067 | [A/G] |
| M6c159627451 | 6 | 159627451 | [A/G] |
| M6c159774074 | 6 | 159774074 | [T/C] |
| M6c160238273 | 6 | 160238273 | [A/G] |
| M6c160607952 | 6 | 160607952 | [A/G] |
| M6c160684473 | 6 | 160684473 | [A/G] |
| M6c161012832 | 6 | 161012832 | [A/G] |
| M6c161556408 | 6 | 161556408 | [A/G] |
| M6c161556974 | 6 | 161556974 | [T/C] |
| M6c161895213 | 6 | 161895213 | [T/C] |
| M6c161967139 | 6 | 161967139 | [A/C] |
| M6c162288778 | 6 | 162288778 | [T/G] |
| M6c162548386 | 6 | 162548386 | [A/G] |
| M6c162931814 | 6 | 162931814 | [T/C] |
| M6c163178351 | 6 | 163178351 | [T/C] |
| M6c163628025 | 6 | 163628025 | [A/G] |
| M6c163988618 | 6 | 163988618 | [T/C] |
| M6c164141982 | 6 | 164141982 | [T/C] |
| M6c164497639 | 6 | 164497639 | [T/C] |
| M6c164891789 | 6 | 164891789 | [A/C] |
| M6c165089848 | 6 | 165089848 | [T/C] |
| M6c165306938 | 6 | 165306938 | [A/G] |
| M6c165390165 | 6 | 165390165 | [A/C] |
| M6c165534841 | 6 | 165534841 | [T/C] |
| M6c165806991 | 6 | 165806991 | [T/C] |
| M6c165944788 | 6 | 165944788 | [T/C] |
| M6c166054503 | 6 | 166054503 | [T/G] |
| M6c166168043 | 6 | 166168043 | [A/C] |
| M6c167113389 | 6 | 167113389 | [T/G] |
| M6c167429690 | 6 | 167429690 | [A/G] |
| M6c167845461 | 6 | 167845461 | [T/G] |
| M6c168100038 | 6 | 168100038 | [A/G] |
| M6c168975400 | 6 | 168975400 | [T/C] |
| M7c305828 | 7 | 305828 | [A/G] |
| M7c572703 | 7 | 572703 | [A/G] |
| M7c922176 | 7 | 922176 | [A/C] |
| M7c1226773 | 7 | 1226773 | [A/G] |
| M7c1519419 | 7 | 1519419 | [A/G] |
| M7c1789731 | 7 | 1789731 | [A/G] |
| M7c2140961 | 7 | 2140961 | [T/G] |
| M7c2776648 | 7 | 2776648 | [A/G] |
| M7c2952590 | 7 | 2952590 | [A/G] |
| M7c3190675 | 7 | 3190675 | [T/C] |
| M7c3669841 | 7 | 3669841 | [T/G] |
| M7c4161483 | 7 | 4161483 | [A/G] |
| M7c4239033 | 7 | 4239033 | [T/C] |
| M7c4703507 | 7 | 4703507 | [A/G] |
| M7c5075308 | 7 | 5075308 | [T/C] |
| M7c5095978 | 7 | 5095978 | [T/C] |
| M7c5563738 | 7 | 5563738 | [A/G] |
| M7c6139066 | 7 | 6139066 | [T/G] |
| M7c6539886 | 7 | 6539886 | [T/C] |
| M7c7118226 | 7 | 7118226 | [A/G] |
| M7c7507002 | 7 | 7507002 | [A/G] |
| M7c7955099 | 7 | 7955099 | [T/C] |
| M7c8178591 | 7 | 8178591 | [T/C] |
| M7c8572056 | 7 | 8572056 | [A/G] |
| M7c9330613 | 7 | 9330613 | [T/C] |
| M7c9914353 | 7 | 9914353 | [A/C] |
| M7c10288416 | 7 | 10288416 | [A/G] |
| M7c10900877 | 7 | 10900877 | [A/G] |
| M7c11149106 | 7 | 11149106 | [T/C] |
| M7c11920630 | 7 | 11920630 | [T/C] |
| M7c12079399 | 7 | 12079399 | [T/G] |
| M7c12523342 | 7 | 12523342 | [T/G] |
| M7c12870062 | 7 | 12870062 | [T/G] |
| M7c13206797 | 7 | 13206797 | [A/G] |
| M7c13578006 | 7 | 13578006 | [A/C] |
| M7c13852099 | 7 | 13852099 | [T/C] |
| M7c14465762 | 7 | 14465762 | [T/C] |
| M7c14617716 | 7 | 14617716 | [T/C] |
| M7c15076259 | 7 | 15076259 | [A/G] |
| M7c15281209 | 7 | 15281209 | [T/C] |
| M7c15660185 | 7 | 15660185 | [T/C] |
| M7c15897519 | 7 | 15897519 | [T/G] |
| M7c16466667 | 7 | 16466667 | [T/C] |
| M7c17182053 | 7 | 17182053 | [T/G] |
| M7c17254577 | 7 | 17254577 | [A/C] |
| M7c17704034 | 7 | 17704034 | [A/G] |
| M7c18222537 | 7 | 18222537 | [A/G] |
| M7c18427575 | 7 | 18427575 | [A/G] |
| M7c19255138 | 7 | 19255138 | [T/C] |
| M7c19752438 | 7 | 19752438 | [T/C] |
| M7c20197145 | 7 | 20197145 | [T/G] |
| M7c20584652 | 7 | 20584652 | [A/C] |
| M7c20857452 | 7 | 20857452 | [T/C] |
| M7c21189532 | 7 | 21189532 | [T/C] |
| M7c21514201 | 7 | 21514201 | [A/C] |
| M7c22014933 | 7 | 22014933 | [A/G] |
| M7c22160099 | 7 | 22160099 | [A/G] |
| M7c22518293 | 7 | 22518293 | [T/C] |
| M7c23234673 | 7 | 23234673 | [T/C] |
| M7c23537916 | 7 | 23537916 | [A/G] |
| M7c23759654 | 7 | 23759654 | [A/C] |
| M7c24413022 | 7 | 24413022 | [A/G] |
| M7c24563199 | 7 | 24563199 | [A/C] |
| M7c24795393 | 7 | 24795393 | [A/C] |
| M7c25202565 | 7 | 25202565 | [A/C] |
| M7c25493811 | 7 | 25493811 | [A/G] |
| M7c25804557 | 7 | 25804557 | [T/G] |
| M7c26228809 | 7 | 26228809 | [A/G] |
| M7c26774052 | 7 | 26774052 | [A/G] |
| M7c27420345 | 7 | 27420345 | [A/G] |
| M7c27660250 | 7 | 27660250 | [T/C] |
| M7c27982827 | 7 | 27982827 | [T/G] |
| M7c28399473 | 7 | 28399473 | [A/G] |
| M7c28590239 | 7 | 28590239 | [A/G] |
| M7c29168190 | 7 | 29168190 | [A/G] |
| M7c29396729 | 7 | 29396729 | [A/C] |
| M7c29655541 | 7 | 29655541 | [T/C] |
| M7c30057357 | 7 | 30057357 | [A/C] |
| M7c30504976 | 7 | 30504976 | [T/C] |
| M7c30674510 | 7 | 30674510 | [A/G] |
| M7c31144580 | 7 | 31144580 | [A/G] |
| M7c31305158 | 7 | 31305158 | [T/G] |
| M7c31911899 | 7 | 31911899 | [T/C] |
| M7c32243730 | 7 | 32243730 | [A/G] |
| M7c32632766 | 7 | 32632766 | [A/G] |
| M7c32735013 | 7 | 32735013 | [T/C] |
| M7c33228368 | 7 | 33228368 | [A/G] |
| M7c33446700 | 7 | 33446700 | [A/G] |
| M7c33950296 | 7 | 33950296 | [A/G] |
| M7c34374171 | 7 | 34374171 | [A/G] |
| M7c34634253 | 7 | 34634253 | [T/C] |
| M7c35010999 | 7 | 35010999 | [T/C] |
| M7c35081157 | 7 | 35081157 | [A/C] |
| M7c35782485 | 7 | 35782485 | [T/G] |
| M7c36208843 | 7 | 36208843 | [A/G] |
| M7c36629687 | 7 | 36629687 | [T/C] |
| M7c37025245 | 7 | 37025245 | [T/C] |
| M7c38083323 | 7 | 38083323 | [T/C] |
| M7c38207980 | 7 | 38207980 | [A/G] |
| M7c38665813 | 7 | 38665813 | [T/C] |
| M7c38898534 | 7 | 38898534 | [T/C] |
| M7c39524731 | 7 | 39524731 | [A/G] |
| M7c39628924 | 7 | 39628924 | [T/C] |
| M7c40114236 | 7 | 40114236 | [A/G] |
| M7c40379321 | 7 | 40379321 | [A/G] |
| M7c40579599 | 7 | 40579599 | [T/C] |
| M7c41170942 | 7 | 41170942 | [A/G] |
| M7c41539519 | 7 | 41539519 | [A/C] |
| M7c41731799 | 7 | 41731799 | [T/C] |
| M7c42228994 | 7 | 42228994 | [T/C] |
| M7c42479106 | 7 | 42479106 | [A/G] |
| M7c42761113 | 7 | 42761113 | [A/G] |
| M7c43121603 | 7 | 43121603 | [A/G] |
| M7c43499911 | 7 | 43499911 | [A/G] |
| M7c43705583 | 7 | 43705583 | [A/C] |
| M7c44179133 | 7 | 44179133 | [T/C] |
| M7c44452930 | 7 | 44452930 | [T/C] |
| M7c45129377 | 7 | 45129377 | [A/G] |
| M7c45787712 | 7 | 45787712 | [T/C] |
| M7c46335111 | 7 | 46335111 | [T/C] |
| M7c46481989 | 7 | 46481989 | [T/C] |
| M7c47005344 | 7 | 47005344 | [T/G] |
| M7c47749540 | 7 | 47749540 | [A/G] |
| M7c47927273 | 7 | 47927273 | [T/G] |
| M7c48201844 | 7 | 48201844 | [T/C] |
| M7c48540127 | 7 | 48540127 | [T/C] |
| M7c49099759 | 7 | 49099759 | [A/G] |
| M7c49732128 | 7 | 49732128 | [T/C] |
| M7c50150298 | 7 | 50150298 | [A/G] |
| M7c50482640 | 7 | 50482640 | [T/C] |
| M7c50521609 | 7 | 50521609 | [T/C] |
| M7c51471492 | 7 | 51471492 | [T/C] |
| M7c51650460 | 7 | 51650460 | [A/C] |
| M7c52005546 | 7 | 52005546 | [T/C] |
| M7c52281569 | 7 | 52281569 | [A/C] |
| M7c52679385 | 7 | 52679385 | [A/C] |
| M7c53021984 | 7 | 53021984 | [T/C] |
| M7c53268038 | 7 | 53268038 | [A/C] |
| M7c53667599 | 7 | 53667599 | [T/C] |
| M7c54195456 | 7 | 54195456 | [T/C] |
| M7c54484556 | 7 | 54484556 | [T/C] |
| M7c54631383 | 7 | 54631383 | [T/C] |
| M7c55275001 | 7 | 55275001 | [T/C] |
| M7c55612340 | 7 | 55612340 | [A/C] |
| M7c56068056 | 7 | 56068056 | [T/C] |
| M7c56420894 | 7 | 56420894 | [T/C] |
| M7c56864568 | 7 | 56864568 | [T/C] |
| M7c57146561 | 7 | 57146561 | [T/C] |
| M7c57627369 | 7 | 57627369 | [T/C] |
| M7c58467238 | 7 | 58467238 | [T/C] |
| M7c58926739 | 7 | 58926739 | [A/G] |
| M7c59635640 | 7 | 59635640 | [A/G] |
| M7c60103076 | 7 | 60103076 | [T/C] |
| M7c60252863 | 7 | 60252863 | [A/G] |
| M7c60626578 | 7 | 60626578 | [A/G] |
| M7c60886780 | 7 | 60886780 | [T/C] |
| M7c61292890 | 7 | 61292890 | [T/C] |
| M7c61731777 | 7 | 61731777 | [T/G] |
| M7c62117753 | 7 | 62117753 | [T/C] |
| M7c62320676 | 7 | 62320676 | [A/G] |
| M7c62627533 | 7 | 62627533 | [T/G] |
| M7c63025170 | 7 | 63025170 | [A/C] |
| M7c63311886 | 7 | 63311886 | [T/C] |
| M7c63976081 | 7 | 63976081 | [T/C] |
| M7c64235830 | 7 | 64235830 | [A/G] |
| M7c64724006 | 7 | 64724006 | [A/G] |
| M7c65214253 | 7 | 65214253 | [T/C] |
| M7c65442481 | 7 | 65442481 | [T/C] |
| M7c66433087 | 7 | 66433087 | [A/G] |
| M7c66951608 | 7 | 66951608 | [A/G] |
| M7c67069599 | 7 | 67069599 | [T/C] |
| M7c67745153 | 7 | 67745153 | [A/G] |
| M7c68866257 | 7 | 68866257 | [T/C] |
| M7c69214494 | 7 | 69214494 | [T/C] |
| M7c69698152 | 7 | 69698152 | [A/G] |
| M7c69790389 | 7 | 69790389 | [T/G] |
| M7c70199305 | 7 | 70199305 | [A/C] |
| M7c70712557 | 7 | 70712557 | [T/C] |
| M7c70758391 | 7 | 70758391 | [A/G] |
| M7c71176655 | 7 | 71176655 | [A/G] |
| M7c72145180 | 7 | 72145180 | [A/C] |
| M7c73026495 | 7 | 73026495 | [A/G] |
| M7c73567363 | 7 | 73567363 | [T/G] |
| M7c74117551 | 7 | 74117551 | [T/C] |
| M7c75489466 | 7 | 75489466 | [A/G] |
| M7c75571503 | 7 | 75571503 | [A/G] |
| M7c76008491 | 7 | 76008491 | [A/G] |
| M7c76293342 | 7 | 76293342 | [T/C] |
| M7c76744080 | 7 | 76744080 | [A/C] |
| M7c77176908 | 7 | 77176908 | [A/C] |
| M7c77458314 | 7 | 77458314 | [T/C] |
| M7c77866948 | 7 | 77866948 | [T/C] |
| M7c78343239 | 7 | 78343239 | [T/C] |
| M7c78722037 | 7 | 78722037 | [A/G] |
| M7c79008298 | 7 | 79008298 | [T/C] |
| M7c79335317 | 7 | 79335317 | [T/G] |
| M7c79701745 | 7 | 79701745 | [T/C] |
| M7c80166143 | 7 | 80166143 | [A/C] |
| M7c80682263 | 7 | 80682263 | [A/G] |
| M7c80799838 | 7 | 80799838 | [A/C] |
| M7c81273712 | 7 | 81273712 | [T/C] |
| M7c81618468 | 7 | 81618468 | [T/C] |
| M7c81845978 | 7 | 81845978 | [A/G] |
| M7c82334320 | 7 | 82334320 | [A/G] |
| M7c83048703 | 7 | 83048703 | [T/G] |
| M7c83147843 | 7 | 83147843 | [T/C] |
| M7c83767156 | 7 | 83767156 | [T/C] |
| M7c83936319 | 7 | 83936319 | [A/G] |
| M7c84368559 | 7 | 84368559 | [A/C] |
| M7c84814015 | 7 | 84814015 | [T/C] |
| M7c84958618 | 7 | 84958618 | [A/G] |
| M7c85162888 | 7 | 85162888 | [A/C] |
| M7c85638796 | 7 | 85638796 | [T/C] |
| M7c85852147 | 7 | 85852147 | [A/C] |
| M7c86434311 | 7 | 86434311 | [A/G] |
| M7c86731047 | 7 | 86731047 | [T/C] |
| M7c87192984 | 7 | 87192984 | [T/C] |
| M7c87515357 | 7 | 87515357 | [T/C] |
| M7c88107200 | 7 | 88107200 | [T/C] |
| M7c88527393 | 7 | 88527393 | [T/G] |
| M7c88736562 | 7 | 88736562 | [T/C] |
| M7c89036166 | 7 | 89036166 | [T/C] |
| M7c89711996 | 7 | 89711996 | [T/C] |
| M7c89963912 | 7 | 89963912 | [A/G] |
| M7c90546267 | 7 | 90546267 | [A/G] |
| M7c91642186 | 7 | 91642186 | [T/C] |
| M7c91973933 | 7 | 91973933 | [T/C] |
| M7c92078793 | 7 | 92078793 | [A/C] |
| M7c92392276 | 7 | 92392276 | [T/C] |
| M7c92943233 | 7 | 92943233 | [T/G] |
| M7c93227389 | 7 | 93227389 | [T/C] |
| M7c93643148 | 7 | 93643148 | [T/C] |
| M7c94022641 | 7 | 94022641 | [T/C] |
| M7c94250285 | 7 | 94250285 | [T/G] |
| M7c95086786 | 7 | 95086786 | [T/C] |
| M7c95447274 | 7 | 95447274 | [A/C] |
| M7c95696378 | 7 | 95696378 | [T/G] |
| M7c96050282 | 7 | 96050282 | [A/C] |
| M7c96469464 | 7 | 96469464 | [A/G] |
| M7c97346665 | 7 | 97346665 | [A/C] |
| M7c97744991 | 7 | 97744991 | [A/G] |
| M7c98249390 | 7 | 98249390 | [A/G] |
| M7c98556687 | 7 | 98556687 | [A/G] |
| M7c99090174 | 7 | 99090174 | [T/C] |
| M7c100005800 | 7 | 100005800 | [T/C] |
| M7c100797113 | 7 | 100797113 | [T/C] |
| M7c101576003 | 7 | 101576003 | [T/C] |
| M7c101741593 | 7 | 101741593 | [T/C] |
| M7c102008101 | 7 | 102008101 | [A/G] |
| M7c102651338 | 7 | 102651338 | [T/C] |
| M7c102869329 | 7 | 102869329 | [A/G] |
| M7c103277338 | 7 | 103277338 | [T/C] |
| M7c103505090 | 7 | 103505090 | [A/G] |
| M7c103946899 | 7 | 103946899 | [T/C] |
| M7c104259673 | 7 | 104259673 | [A/G] |
| M7c104400605 | 7 | 104400605 | [T/C] |
| M7c104884259 | 7 | 104884259 | [A/C] |
| M7c105610977 | 7 | 105610977 | [T/C] |
| M7c105764579 | 7 | 105764579 | [T/C] |
| M7c106231008 | 7 | 106231008 | [A/G] |
| M7c106749932 | 7 | 106749932 | [A/C] |
| M7c106998922 | 7 | 106998922 | [T/C] |
| M7c107233560 | 7 | 107233560 | [A/C] |
| M7c107650274 | 7 | 107650274 | [T/C] |
| M7c107841613 | 7 | 107841613 | [A/G] |
| M7c108481433 | 7 | 108481433 | [A/G] |
| M7c108535388 | 7 | 108535388 | [T/C] |
| M7c109048037 | 7 | 109048037 | [A/G] |
| M7c109326608 | 7 | 109326608 | [T/C] |
| M7c109329336 | 7 | 109329336 | [T/G] |
| M7c110051699 | 7 | 110051699 | [A/C] |
| M7c110298597 | 7 | 110298597 | [A/C] |
| M7c111130665 | 7 | 111130665 | [A/G] |
| M7c111341996 | 7 | 111341996 | [A/G] |
| M7c111664706 | 7 | 111664706 | [A/G] |
| M7c112085841 | 7 | 112085841 | [T/C] |
| M7c112405199 | 7 | 112405199 | [T/C] |
| M7c112875744 | 7 | 112875744 | [A/C] |
| M7c113165595 | 7 | 113165595 | [A/C] |
| M7c113705584 | 7 | 113705584 | [A/G] |
| M7c114347369 | 7 | 114347369 | [T/G] |
| M7c114986415 | 7 | 114986415 | [T/G] |
| M7c115183983 | 7 | 115183983 | [A/G] |
| M7c115693602 | 7 | 115693602 | [T/G] |
| M7c116288821 | 7 | 116288821 | [A/G] |
| M7c116382760 | 7 | 116382760 | [T/C] |
| M7c117017969 | 7 | 117017969 | [A/G] |
| M7c117238053 | 7 | 117238053 | [A/C] |
| M7c117701400 | 7 | 117701400 | [T/C] |
| M7c117982889 | 7 | 117982889 | [A/C] |
| M7c118427335 | 7 | 118427335 | [T/C] |
| M7c118582946 | 7 | 118582946 | [T/C] |
| M7c119042583 | 7 | 119042583 | [A/C] |
| M7c119617887 | 7 | 119617887 | [A/G] |
| M7c119950563 | 7 | 119950563 | [T/C] |
| M7c120205996 | 7 | 120205996 | [T/C] |
| M7c120537733 | 7 | 120537733 | [T/C] |
| M7c121016162 | 7 | 121016162 | [T/C] |
| M7c121577528 | 7 | 121577528 | [A/G] |
| M7c121986629 | 7 | 121986629 | [T/C] |
| M7c122331140 | 7 | 122331140 | [T/C] |
| M7c122829624 | 7 | 122829624 | [T/C] |
| M7c123695954 | 7 | 123695954 | [A/G] |
| M7c124211662 | 7 | 124211662 | [T/C] |
| M7c124386720 | 7 | 124386720 | [A/G] |
| M7c124754106 | 7 | 124754106 | [T/C] |
| M7c125103532 | 7 | 125103532 | [T/C] |
| M7c125973999 | 7 | 125973999 | [A/G] |
| M7c126222588 | 7 | 126222588 | [A/G] |
| M7c126446881 | 7 | 126446881 | [A/G] |
| M7c126911957 | 7 | 126911957 | [A/G] |
| M7c127248316 | 7 | 127248316 | [A/C] |
| M7c127576648 | 7 | 127576648 | [T/C] |
| M7c127858112 | 7 | 127858112 | [A/G] |
| M7c128434862 | 7 | 128434862 | [T/C] |
| M7c128896749 | 7 | 128896749 | [T/G] |
| M7c129450890 | 7 | 129450890 | [T/C] |
| M7c129967901 | 7 | 129967901 | [T/C] |
| M7c130242120 | 7 | 130242120 | [A/C] |
| M7c130784233 | 7 | 130784233 | [T/C] |
| M7c131195688 | 7 | 131195688 | [A/G] |
| M7c132057910 | 7 | 132057910 | [A/G] |
| M7c132164876 | 7 | 132164876 | [T/C] |
| M7c133051248 | 7 | 133051248 | [T/C] |
| M7c133564691 | 7 | 133564691 | [T/C] |
| M7c134127332 | 7 | 134127332 | [T/C] |
| M7c134226995 | 7 | 134226995 | [A/G] |
| M7c134843614 | 7 | 134843614 | [A/G] |
| M7c134971250 | 7 | 134971250 | [T/C] |
| M7c135557732 | 7 | 135557732 | [T/C] |
| M7c135836595 | 7 | 135836595 | [A/C] |
| M7c136267813 | 7 | 136267813 | [A/G] |
| M7c136571499 | 7 | 136571499 | [T/G] |
| M7c136947032 | 7 | 136947032 | [A/G] |
| M7c137168930 | 7 | 137168930 | [T/C] |
| M7c137455293 | 7 | 137455293 | [T/C] |
| M7c137701653 | 7 | 137701653 | [T/C] |
| M7c138098415 | 7 | 138098415 | [A/G] |
| M7c138735403 | 7 | 138735403 | [A/G] |
| M7c139356056 | 7 | 139356056 | [A/G] |
| M7c139761005 | 7 | 139761005 | [A/G] |
| M7c140104604 | 7 | 140104604 | [T/C] |
| M7c140419060 | 7 | 140419060 | [T/C] |
| M7c140818910 | 7 | 140818910 | [T/C] |
| M7c141641765 | 7 | 141641765 | [A/G] |
| M7c141901759 | 7 | 141901759 | [A/G] |
| M7c142407403 | 7 | 142407403 | [A/G] |
| M7c142726091 | 7 | 142726091 | [T/C] |
| M7c142854109 | 7 | 142854109 | [A/G] |
| M7c143425558 | 7 | 143425558 | [T/C] |
| M7c143747435 | 7 | 143747435 | [T/C] |
| M7c144129724 | 7 | 144129724 | [T/C] |
| M7c144504054 | 7 | 144504054 | [A/G] |
| M7c144658109 | 7 | 144658109 | [T/C] |
| M7c144865074 | 7 | 144865074 | [A/G] |
| M7c145304415 | 7 | 145304415 | [T/C] |
| M7c145682191 | 7 | 145682191 | [T/C] |
| M7c145969407 | 7 | 145969407 | [T/C] |
| M7c146492350 | 7 | 146492350 | [A/G] |
| M7c146596371 | 7 | 146596371 | [T/C] |
| M7c147133123 | 7 | 147133123 | [A/G] |
| M7c147582156 | 7 | 147582156 | [A/G] |
| M7c147773647 | 7 | 147773647 | [T/C] |
| M7c148175527 | 7 | 148175527 | [A/G] |
| M7c148623193 | 7 | 148623193 | [T/G] |
| M7c148850510 | 7 | 148850510 | [T/C] |
| M7c149489645 | 7 | 149489645 | [A/G] |
| M7c149858610 | 7 | 149858610 | [A/G] |
| M7c150040898 | 7 | 150040898 | [T/C] |
| M7c150455008 | 7 | 150455008 | [T/C] |
| M7c150879186 | 7 | 150879186 | [A/G] |
| M7c151242710 | 7 | 151242710 | [T/C] |
| M7c151409024 | 7 | 151409024 | [A/G] |
| M7c151853568 | 7 | 151853568 | [T/G] |
| M7c152065328 | 7 | 152065328 | [A/C] |
| M7c152692236 | 7 | 152692236 | [A/C] |
| M7c152952264 | 7 | 152952264 | [A/G] |
| M7c153285819 | 7 | 153285819 | [A/G] |
| M7c153734680 | 7 | 153734680 | [A/G] |
| M7c153821238 | 7 | 153821238 | [A/G] |
| M7c154468297 | 7 | 154468297 | [A/C] |
| M7c155071955 | 7 | 155071955 | [T/C] |
| M7c155341864 | 7 | 155341864 | [T/C] |
| M7c155592075 | 7 | 155592075 | [A/G] |
| M7c156026560 | 7 | 156026560 | [A/C] |
| M7c157160236 | 7 | 157160236 | [T/G] |
| M7c157574880 | 7 | 157574880 | [T/C] |
| M7c158044296 | 7 | 158044296 | [A/C] |
| M7c158464926 | 7 | 158464926 | [T/C] |
| M7c158748821 | 7 | 158748821 | [T/C] |
| M7c159139233 | 7 | 159139233 | [A/G] |
| M7c159236499 | 7 | 159236499 | [A/C] |
| M7c159467059 | 7 | 159467059 | [A/G] |
| M7c159593500 | 7 | 159593500 | [A/C] |
| M7c159593975 | 7 | 159593975 | [A/G] |
| M7c159783022 | 7 | 159783022 | [T/C] |
| M7c160292721 | 7 | 160292721 | [T/C] |
| M7c160816094 | 7 | 160816094 | [A/G] |
| M7c161129382 | 7 | 161129382 | [T/C] |
| M7c161424654 | 7 | 161424654 | [T/C] |
| M7c162669897 | 7 | 162669897 | [A/G] |
| M7c163003155 | 7 | 163003155 | [A/G] |
| M7c163058975 | 7 | 163058975 | [T/C] |
| M7c164241660 | 7 | 164241660 | [A/G] |
| M7c164567706 | 7 | 164567706 | [T/G] |
| M7c164993518 | 7 | 164993518 | [T/C] |
| M7c165243614 | 7 | 165243614 | [T/C] |
| M7c165463878 | 7 | 165463878 | [A/G] |
| M7c165988142 | 7 | 165988142 | [A/G] |
| M7c166388278 | 7 | 166388278 | [T/C] |
| M7c166568198 | 7 | 166568198 | [A/G] |
| M7c167096478 | 7 | 167096478 | [T/C] |
| M7c167345524 | 7 | 167345524 | [T/G] |
| M7c167713959 | 7 | 167713959 | [T/C] |
| M7c167908370 | 7 | 167908370 | [T/C] |
| M7c168290649 | 7 | 168290649 | [T/C] |
| M7c168567037 | 7 | 168567037 | [T/G] |
| M7c169203221 | 7 | 169203221 | [T/C] |
| M7c169263528 | 7 | 169263528 | [T/C] |
| M7c169741705 | 7 | 169741705 | [T/C] |
| M7c170033029 | 7 | 170033029 | [T/C] |
| M7c170246122 | 7 | 170246122 | [T/C] |
| M7c170420168 | 7 | 170420168 | [A/C] |
| M7c170420228 | 7 | 170420228 | [A/G] |
| M7c170436669 | 7 | 170436669 | [A/G] |
| M7c170643801 | 7 | 170643801 | [A/G] |
| M7c170871499 | 7 | 170871499 | [A/C] |
| M7c170955173 | 7 | 170955173 | [A/G] |
| M7c170977014 | 7 | 170977014 | [T/C] |
| M7c171565887 | 7 | 171565887 | [T/C] |
| M7c172119527 | 7 | 172119527 | [T/C] |
| M7c172712657 | 7 | 172712657 | [A/C] |
| M7c173194363 | 7 | 173194363 | [A/G] |
| M7c173448496 | 7 | 173448496 | [A/G] |
| M7c173808340 | 7 | 173808340 | [T/G] |
| M7c174091458 | 7 | 174091458 | [A/C] |
| M7c174225147 | 7 | 174225147 | [A/G] |
| M7c174507110 | 7 | 174507110 | [A/G] |
| M7c174515585 | 7 | 174515585 | [A/C] |
| M7c174745079 | 7 | 174745079 | [A/G] |
| M7c175205654 | 7 | 175205654 | [A/C] |
| M7c175640848 | 7 | 175640848 | [T/G] |
| M7c175864385 | 7 | 175864385 | [T/C] |
| M7c176136715 | 7 | 176136715 | [A/G] |
| M8c172154 | 8 | 172154 | [A/C] |
| M8c502092 | 8 | 502092 | [T/C] |
| M8c870565 | 8 | 870565 | [T/G] |
| M8c1219670 | 8 | 1219670 | [A/C] |
| M8c1448516 | 8 | 1448516 | [A/G] |
| M8c2350307 | 8 | 2350307 | [T/C] |
| M8c2518217 | 8 | 2518217 | [A/G] |
| M8c3203216 | 8 | 3203216 | [T/C] |
| M8c3576462 | 8 | 3576462 | [A/G] |
| M8c3805303 | 8 | 3805303 | [T/G] |
| M8c4282195 | 8 | 4282195 | [A/C] |
| M8c4800966 | 8 | 4800966 | [T/C] |
| M8c4809402 | 8 | 4809402 | [A/G] |
| M8c5161033 | 8 | 5161033 | [T/C] |
| M8c5598954 | 8 | 5598954 | [A/G] |
| M8c6023496 | 8 | 6023496 | [A/G] |
| M8c6480599 | 8 | 6480599 | [T/C] |
| M8c6855751 | 8 | 6855751 | [A/G] |
| M8c6893344 | 8 | 6893344 | [T/C] |
| M8c7431220 | 8 | 7431220 | [A/C] |
| M8c7750230 | 8 | 7750230 | [A/G] |
| M8c7981630 | 8 | 7981630 | [A/G] |
| M8c8293779 | 8 | 8293779 | [T/C] |
| M8c8615653 | 8 | 8615653 | [T/C] |
| M8c8922468 | 8 | 8922468 | [A/C] |
| M8c9450591 | 8 | 9450591 | [T/C] |
| M8c9900802 | 8 | 9900802 | [A/G] |
| M8c10082378 | 8 | 10082378 | [T/C] |
| M8c10298512 | 8 | 10298512 | [A/G] |
| M8c10855837 | 8 | 10855837 | [A/G] |
| M8c11302915 | 8 | 11302915 | [T/C] |
| M8c11535591 | 8 | 11535591 | [A/G] |
| M8c11799562 | 8 | 11799562 | [A/C] |
| M8c12286269 | 8 | 12286269 | [A/C] |
| M8c12555754 | 8 | 12555754 | [T/C] |
| M8c13198203 | 8 | 13198203 | [A/G] |
| M8c13616058 | 8 | 13616058 | [A/G] |
| M8c14087628 | 8 | 14087628 | [A/G] |
| M8c14796286 | 8 | 14796286 | [A/G] |
| M8c15274430 | 8 | 15274430 | [A/G] |
| M8c15549302 | 8 | 15549302 | [A/G] |
| M8c15977698 | 8 | 15977698 | [T/C] |
| M8c16335152 | 8 | 16335152 | [T/C] |
| M8c16691892 | 8 | 16691892 | [A/C] |
| M8c17058319 | 8 | 17058319 | [A/G] |
| M8c17671141 | 8 | 17671141 | [A/G] |
| M8c17872765 | 8 | 17872765 | [T/G] |
| M8c18776391 | 8 | 18776391 | [T/G] |
| M8c19234754 | 8 | 19234754 | [A/G] |
| M8c19805664 | 8 | 19805664 | [A/G] |
| M8c20084476 | 8 | 20084476 | [T/C] |
| M8c20309158 | 8 | 20309158 | [A/C] |
| M8c20974799 | 8 | 20974799 | [T/G] |
| M8c21449015 | 8 | 21449015 | [A/G] |
| M8c21876658 | 8 | 21876658 | [A/G] |
| M8c22502081 | 8 | 22502081 | [A/G] |
| M8c23801897 | 8 | 23801897 | [T/C] |
| M8c24343661 | 8 | 24343661 | [T/C] |
| M8c24411669 | 8 | 24411669 | [A/G] |
| M8c24766999 | 8 | 24766999 | [A/G] |
| M8c26066369 | 8 | 26066369 | [A/G] |
| M8c26583799 | 8 | 26583799 | [A/G] |
| M8c26861642 | 8 | 26861642 | [A/C] |
| M8c27127778 | 8 | 27127778 | [A/G] |
| M8c27650260 | 8 | 27650260 | [A/G] |
| M8c27806843 | 8 | 27806843 | [A/G] |
| M8c28287543 | 8 | 28287543 | [A/G] |
| M8c28654691 | 8 | 28654691 | [T/C] |
| M8c28852577 | 8 | 28852577 | [A/G] |
| M8c29230860 | 8 | 29230860 | [A/G] |
| M8c29732108 | 8 | 29732108 | [T/C] |
| M8c29995043 | 8 | 29995043 | [A/G] |
| M8c30209986 | 8 | 30209986 | [T/C] |
| M8c30746947 | 8 | 30746947 | [T/C] |
| M8c31604670 | 8 | 31604670 | [A/G] |
| M8c32093223 | 8 | 32093223 | [A/G] |
| M8c32570959 | 8 | 32570959 | [T/C] |
| M8c32752092 | 8 | 32752092 | [A/G] |
| M8c32964140 | 8 | 32964140 | [T/G] |
| M8c33452065 | 8 | 33452065 | [T/C] |
| M8c33889441 | 8 | 33889441 | [T/C] |
| M8c34579727 | 8 | 34579727 | [T/G] |
| M8c34664391 | 8 | 34664391 | [A/G] |
| M8c35223185 | 8 | 35223185 | [A/G] |
| M8c35447273 | 8 | 35447273 | [A/G] |
| M8c35814906 | 8 | 35814906 | [A/C] |
| M8c36506674 | 8 | 36506674 | [T/C] |
| M8c36831650 | 8 | 36831650 | [A/G] |
| M8c37223428 | 8 | 37223428 | [T/C] |
| M8c37411521 | 8 | 37411521 | [A/G] |
| M8c37759556 | 8 | 37759556 | [A/G] |
| M8c38488301 | 8 | 38488301 | [A/G] |
| M8c38521846 | 8 | 38521846 | [T/G] |
| M8c39018373 | 8 | 39018373 | [A/G] |
| M8c39210398 | 8 | 39210398 | [A/G] |
| M8c39459157 | 8 | 39459157 | [T/C] |
| M8c40264066 | 8 | 40264066 | [T/G] |
| M8c40678684 | 8 | 40678684 | [T/C] |
| M8c40955129 | 8 | 40955129 | [T/G] |
| M8c41365506 | 8 | 41365506 | [T/C] |
| M8c41701315 | 8 | 41701315 | [T/C] |
| M8c42010780 | 8 | 42010780 | [A/G] |
| M8c42809937 | 8 | 42809937 | [T/G] |
| M8c43079733 | 8 | 43079733 | [T/C] |
| M8c43288844 | 8 | 43288844 | [T/C] |
| M8c43847909 | 8 | 43847909 | [T/G] |
| M8c43977375 | 8 | 43977375 | [T/C] |
| M8c44401700 | 8 | 44401700 | [T/C] |
| M8c45262229 | 8 | 45262229 | [A/C] |
| M8c45441770 | 8 | 45441770 | [T/C] |
| M8c45768991 | 8 | 45768991 | [T/C] |
| M8c46223463 | 8 | 46223463 | [A/G] |
| M8c46593269 | 8 | 46593269 | [A/G] |
| M8c46695597 | 8 | 46695597 | [A/G] |
| M8c47072628 | 8 | 47072628 | [T/C] |
| M8c47622119 | 8 | 47622119 | [T/C] |
| M8c48154647 | 8 | 48154647 | [A/C] |
| M8c48490428 | 8 | 48490428 | [T/C] |
| M8c48874246 | 8 | 48874246 | [T/C] |
| M8c49202862 | 8 | 49202862 | [T/C] |
| M8c49592208 | 8 | 49592208 | [A/G] |
| M8c50074483 | 8 | 50074483 | [T/C] |
| M8c50114393 | 8 | 50114393 | [T/C] |
| M8c51064648 | 8 | 51064648 | [T/C] |
| M8c51418264 | 8 | 51418264 | [A/C] |
| M8c52299672 | 8 | 52299672 | [A/G] |
| M8c52903262 | 8 | 52903262 | [T/G] |
| M8c53329919 | 8 | 53329919 | [T/G] |
| M8c53862197 | 8 | 53862197 | [A/G] |
| M8c54419526 | 8 | 54419526 | [A/G] |
| M8c54607682 | 8 | 54607682 | [T/C] |
| M8c55020791 | 8 | 55020791 | [A/G] |
| M8c55249417 | 8 | 55249417 | [T/C] |
| M8c55721079 | 8 | 55721079 | [A/C] |
| M8c55965516 | 8 | 55965516 | [A/G] |
| M8c56585409 | 8 | 56585409 | [T/C] |
| M8c56662602 | 8 | 56662602 | [T/C] |
| M8c57211276 | 8 | 57211276 | [T/G] |
| M8c57452886 | 8 | 57452886 | [T/C] |
| M8c57888395 | 8 | 57888395 | [A/G] |
| M8c58203691 | 8 | 58203691 | [A/C] |
| M8c58586408 | 8 | 58586408 | [T/C] |
| M8c59470888 | 8 | 59470888 | [T/C] |
| M8c59974937 | 8 | 59974937 | [A/G] |
| M8c60875629 | 8 | 60875629 | [A/G] |
| M8c61389740 | 8 | 61389740 | [T/C] |
| M8c61520980 | 8 | 61520980 | [A/G] |
| M8c62099541 | 8 | 62099541 | [A/G] |
| M8c62285865 | 8 | 62285865 | [T/G] |
| M8c62515892 | 8 | 62515892 | [A/G] |
| M8c62833681 | 8 | 62833681 | [T/C] |
| M8c63268859 | 8 | 63268859 | [A/G] |
| M8c63859252 | 8 | 63859252 | [A/G] |
| M8c64166535 | 8 | 64166535 | [A/G] |
| M8c64660721 | 8 | 64660721 | [T/C] |
| M8c65100497 | 8 | 65100497 | [T/C] |
| M8c65244756 | 8 | 65244756 | [T/C] |
| M8c65784891 | 8 | 65784891 | [A/G] |
| M8c66097903 | 8 | 66097903 | [T/C] |
| M8c66560917 | 8 | 66560917 | [A/G] |
| M8c66665225 | 8 | 66665225 | [T/C] |
| M8c67385005 | 8 | 67385005 | [T/G] |
| M8c67723175 | 8 | 67723175 | [T/C] |
| M8c68087671 | 8 | 68087671 | [A/G] |
| M8c68362579 | 8 | 68362579 | [A/G] |
| M8c68692350 | 8 | 68692350 | [T/G] |
| M8c69130783 | 8 | 69130783 | [A/G] |
| M8c69594106 | 8 | 69594106 | [A/G] |
| M8c69754674 | 8 | 69754674 | [A/G] |
| M8c70136485 | 8 | 70136485 | [T/C] |
| M8c70603420 | 8 | 70603420 | [A/G] |
| M8c70971475 | 8 | 70971475 | [A/G] |
| M8c71278522 | 8 | 71278522 | [A/G] |
| M8c71636309 | 8 | 71636309 | [T/C] |
| M8c71793999 | 8 | 71793999 | [A/G] |
| M8c72067330 | 8 | 72067330 | [T/C] |
| M8c72627942 | 8 | 72627942 | [T/C] |
| M8c72976876 | 8 | 72976876 | [A/G] |
| M8c73090479 | 8 | 73090479 | [T/C] |
| M8c73735651 | 8 | 73735651 | [A/G] |
| M8c73854282 | 8 | 73854282 | [A/C] |
| M8c74437182 | 8 | 74437182 | [T/C] |
| M8c74903169 | 8 | 74903169 | [A/C] |
| M8c75177510 | 8 | 75177510 | [A/G] |
| M8c75599159 | 8 | 75599159 | [A/G] |
| M8c76129277 | 8 | 76129277 | [T/C] |
| M8c76319665 | 8 | 76319665 | [T/C] |
| M8c76643773 | 8 | 76643773 | [A/G] |
| M8c77089526 | 8 | 77089526 | [A/G] |
| M8c77198200 | 8 | 77198200 | [T/C] |
| M8c77752009 | 8 | 77752009 | [A/G] |
| M8c78079487 | 8 | 78079487 | [A/G] |
| M8c78406716 | 8 | 78406716 | [T/C] |
| M8c78646062 | 8 | 78646062 | [A/G] |
| M8c79075248 | 8 | 79075248 | [A/G] |
| M8c79934432 | 8 | 79934432 | [T/C] |
| M8c80226202 | 8 | 80226202 | [T/C] |
| M8c80293263 | 8 | 80293263 | [T/G] |
| M8c80684981 | 8 | 80684981 | [A/G] |
| M8c81246152 | 8 | 81246152 | [A/C] |
| M8c81873888 | 8 | 81873888 | [T/C] |
| M8c82060785 | 8 | 82060785 | [A/G] |
| M8c82378487 | 8 | 82378487 | [T/C] |
| M8c82967102 | 8 | 82967102 | [T/C] |
| M8c83059570 | 8 | 83059570 | [A/C] |
| M8c83438909 | 8 | 83438909 | [T/C] |
| M8c83921245 | 8 | 83921245 | [A/G] |
| M8c84070612 | 8 | 84070612 | [T/C] |
| M8c84608377 | 8 | 84608377 | [A/G] |
| M8c84965089 | 8 | 84965089 | [A/C] |
| M8c85147029 | 8 | 85147029 | [A/G] |
| M8c85670381 | 8 | 85670381 | [T/C] |
| M8c85847000 | 8 | 85847000 | [A/G] |
| M8c86403100 | 8 | 86403100 | [A/G] |
| M8c86746756 | 8 | 86746756 | [T/C] |
| M8c87031101 | 8 | 87031101 | [A/G] |
| M8c87238943 | 8 | 87238943 | [A/C] |
| M8c88043662 | 8 | 88043662 | [T/C] |
| M8c88509295 | 8 | 88509295 | [T/C] |
| M8c88546562 | 8 | 88546562 | [A/C] |
| M8c89432129 | 8 | 89432129 | [T/C] |
| M8c90833852 | 8 | 90833852 | [A/G] |
| M8c90966443 | 8 | 90966443 | [T/C] |
| M8c91391388 | 8 | 91391388 | [A/G] |
| M8c91616503 | 8 | 91616503 | [T/C] |
| M8c92116968 | 8 | 92116968 | [T/C] |
| M8c92615561 | 8 | 92615561 | [T/C] |
| M8c92700876 | 8 | 92700876 | [T/C] |
| M8c93221045 | 8 | 93221045 | [T/G] |
| M8c93492337 | 8 | 93492337 | [T/C] |
| M8c93927788 | 8 | 93927788 | [A/G] |
| M8c94437746 | 8 | 94437746 | [A/G] |
| M8c95014435 | 8 | 95014435 | [A/G] |
| M8c95096811 | 8 | 95096811 | [A/G] |
| M8c95715286 | 8 | 95715286 | [A/G] |
| M8c95775581 | 8 | 95775581 | [A/C] |
| M8c96270058 | 8 | 96270058 | [A/G] |
| M8c96630540 | 8 | 96630540 | [T/C] |
| M8c97177521 | 8 | 97177521 | [A/G] |
| M8c97777183 | 8 | 97777183 | [T/G] |
| M8c98029337 | 8 | 98029337 | [A/G] |
| M8c98420651 | 8 | 98420651 | [A/G] |
| M8c98598093 | 8 | 98598093 | [A/G] |
| M8c98995088 | 8 | 98995088 | [T/G] |
| M8c99160143 | 8 | 99160143 | [T/G] |
| M8c99753107 | 8 | 99753107 | [A/G] |
| M8c99875713 | 8 | 99875713 | [A/G] |
| M8c100397006 | 8 | 100397006 | [T/C] |
| M8c100869494 | 8 | 100869494 | [T/C] |
| M8c100960678 | 8 | 100960678 | [A/G] |
| M8c100991704 | 8 | 100991704 | [T/C] |
| M8c101416842 | 8 | 101416842 | [A/G] |
| M8c101756699 | 8 | 101756699 | [T/C] |
| M8c101958403 | 8 | 101958403 | [A/G] |
| M8c102404170 | 8 | 102404170 | [A/G] |
| M8c102702708 | 8 | 102702708 | [A/G] |
| M8c103187595 | 8 | 103187595 | [T/C] |
| M8c103593691 | 8 | 103593691 | [T/C] |
| M8c103704257 | 8 | 103704257 | [A/G] |
| M8c104281609 | 8 | 104281609 | [T/C] |
| M8c104625009 | 8 | 104625009 | [A/G] |
| M8c104663885 | 8 | 104663885 | [A/G] |
| M8c104771075 | 8 | 104771075 | [A/G] |
| M8c105322519 | 8 | 105322519 | [T/C] |
| M8c105567853 | 8 | 105567853 | [T/C] |
| M8c105781102 | 8 | 105781102 | [A/G] |
| M8c106208165 | 8 | 106208165 | [T/C] |
| M8c106675160 | 8 | 106675160 | [T/C] |
| M8c106734047 | 8 | 106734047 | [A/G] |
| M8c107224728 | 8 | 107224728 | [A/G] |
| M8c107497952 | 8 | 107497952 | [T/C] |
| M8c107878959 | 8 | 107878959 | [T/C] |
| M8c108322074 | 8 | 108322074 | [T/C] |
| M8c108494127 | 8 | 108494127 | [A/G] |
| M8c109012220 | 8 | 109012220 | [A/G] |
| M8c109164234 | 8 | 109164234 | [A/G] |
| M8c109934768 | 8 | 109934768 | [A/G] |
| M8c110214943 | 8 | 110214943 | [T/G] |
| M8c110988703 | 8 | 110988703 | [T/G] |
| M8c111175828 | 8 | 111175828 | [T/C] |
| M8c112150371 | 8 | 112150371 | [T/C] |
| M8c112311194 | 8 | 112311194 | [T/C] |
| M8c112713081 | 8 | 112713081 | [A/G] |
| M8c112902020 | 8 | 112902020 | [A/C] |
| M8c113347217 | 8 | 113347217 | [A/G] |
| M8c114015081 | 8 | 114015081 | [T/C] |
| M8c114430712 | 8 | 114430712 | [A/G] |
| M8c114643826 | 8 | 114643826 | [T/C] |
| M8c115129154 | 8 | 115129154 | [A/G] |
| M8c115638510 | 8 | 115638510 | [A/C] |
| M8c116024315 | 8 | 116024315 | [T/G] |
| M8c116442228 | 8 | 116442228 | [T/C] |
| M8c117166216 | 8 | 117166216 | [A/G] |
| M8c117573108 | 8 | 117573108 | [A/C] |
| M8c117869841 | 8 | 117869841 | [T/C] |
| M8c118190281 | 8 | 118190281 | [T/C] |
| M8c118530560 | 8 | 118530560 | [T/C] |
| M8c118778230 | 8 | 118778230 | [T/C] |
| M8c119255879 | 8 | 119255879 | [T/C] |
| M8c119494453 | 8 | 119494453 | [T/C] |
| M8c119745467 | 8 | 119745467 | [T/C] |
| M8c120241582 | 8 | 120241582 | [A/G] |
| M8c120875063 | 8 | 120875063 | [T/C] |
| M8c121436050 | 8 | 121436050 | [T/G] |
| M8c121764423 | 8 | 121764423 | [T/C] |
| M8c121895690 | 8 | 121895690 | [A/G] |
| M8c122146557 | 8 | 122146557 | [A/G] |
| M8c122753764 | 8 | 122753764 | [T/C] |
| M8c123512667 | 8 | 123512667 | [A/G] |
| M8c123607700 | 8 | 123607700 | [A/G] |
| M8c123912576 | 8 | 123912576 | [T/C] |
| M8c124462202 | 8 | 124462202 | [T/C] |
| M8c124797701 | 8 | 124797701 | [T/G] |
| M8c124938918 | 8 | 124938918 | [T/C] |
| M8c125298160 | 8 | 125298160 | [T/C] |
| M8c126083118 | 8 | 126083118 | [T/C] |
| M8c126491865 | 8 | 126491865 | [A/G] |
| M8c126701658 | 8 | 126701658 | [A/C] |
| M8c126992864 | 8 | 126992864 | [T/G] |
| M8c127965254 | 8 | 127965254 | [T/C] |
| M8c128128151 | 8 | 128128151 | [A/G] |
| M8c128547329 | 8 | 128547329 | [T/C] |
| M8c128992054 | 8 | 128992054 | [T/C] |
| M8c129291459 | 8 | 129291459 | [T/C] |
| M8c129504901 | 8 | 129504901 | [T/C] |
| M8c130211116 | 8 | 130211116 | [T/C] |
| M8c130512645 | 8 | 130512645 | [A/G] |
| M8c131041704 | 8 | 131041704 | [A/G] |
| M8c131171351 | 8 | 131171351 | [A/G] |
| M8c131519246 | 8 | 131519246 | [A/G] |
| M8c131799218 | 8 | 131799218 | [A/C] |
| M8c132171120 | 8 | 132171120 | [A/C] |
| M8c132519801 | 8 | 132519801 | [T/G] |
| M8c132877552 | 8 | 132877552 | [T/G] |
| M8c133181850 | 8 | 133181850 | [T/C] |
| M8c133566290 | 8 | 133566290 | [T/C] |
| M8c134741415 | 8 | 134741415 | [T/C] |
| M8c135062783 | 8 | 135062783 | [T/C] |
| M8c136415591 | 8 | 136415591 | [A/G] |
| M8c136659701 | 8 | 136659701 | [A/G] |
| M8c136971423 | 8 | 136971423 | [A/G] |
| M8c137467990 | 8 | 137467990 | [A/C] |
| M8c137758795 | 8 | 137758795 | [T/C] |
| M8c138142972 | 8 | 138142972 | [A/G] |
| M8c138514768 | 8 | 138514768 | [A/C] |
| M8c138668436 | 8 | 138668436 | [T/G] |
| M8c139105125 | 8 | 139105125 | [T/C] |
| M8c139888272 | 8 | 139888272 | [T/C] |
| M8c140183830 | 8 | 140183830 | [T/C] |
| M8c140385924 | 8 | 140385924 | [A/G] |
| M8c140752324 | 8 | 140752324 | [T/G] |
| M8c141255108 | 8 | 141255108 | [A/G] |
| M8c141393041 | 8 | 141393041 | [T/G] |
| M8c141990105 | 8 | 141990105 | [T/C] |
| M8c142236003 | 8 | 142236003 | [A/G] |
| M8c142500505 | 8 | 142500505 | [T/C] |
| M8c142843188 | 8 | 142843188 | [T/C] |
| M8c143158637 | 8 | 143158637 | [T/C] |
| M8c143449539 | 8 | 143449539 | [T/C] |
| M8c144021136 | 8 | 144021136 | [T/C] |
| M8c144374871 | 8 | 144374871 | [T/C] |
| M8c144539873 | 8 | 144539873 | [T/G] |
| M8c144880796 | 8 | 144880796 | [A/G] |
| M8c145280826 | 8 | 145280826 | [A/G] |
| M8c145798714 | 8 | 145798714 | [T/G] |
| M8c146030749 | 8 | 146030749 | [A/C] |
| M8c146187405 | 8 | 146187405 | [A/G] |
| M8c146617849 | 8 | 146617849 | [A/G] |
| M8c146990749 | 8 | 146990749 | [A/G] |
| M8c147292989 | 8 | 147292989 | [A/C] |
| M8c147726125 | 8 | 147726125 | [T/G] |
| M8c148086023 | 8 | 148086023 | [T/C] |
| M8c148480071 | 8 | 148480071 | [A/C] |
| M8c148676361 | 8 | 148676361 | [A/G] |
| M8c149037804 | 8 | 149037804 | [T/G] |
| M8c149926124 | 8 | 149926124 | [A/G] |
| M8c150137873 | 8 | 150137873 | [T/G] |
| M8c150448806 | 8 | 150448806 | [T/C] |
| M8c151312347 | 8 | 151312347 | [A/G] |
| M8c151740869 | 8 | 151740869 | [A/G] |
| M8c152144735 | 8 | 152144735 | [A/C] |
| M8c152593017 | 8 | 152593017 | [A/C] |
| M8c152705529 | 8 | 152705529 | [T/C] |
| M8c153089567 | 8 | 153089567 | [A/G] |
| M8c153595674 | 8 | 153595674 | [A/G] |
| M8c153794835 | 8 | 153794835 | [T/C] |
| M8c154246272 | 8 | 154246272 | [A/G] |
| M8c154736699 | 8 | 154736699 | [T/C] |
| M8c155652608 | 8 | 155652608 | [T/G] |
| M8c156039730 | 8 | 156039730 | [A/G] |
| M8c156441541 | 8 | 156441541 | [A/G] |
| M8c156774171 | 8 | 156774171 | [T/C] |
| M8c156822646 | 8 | 156822646 | [T/C] |
| M8c157469391 | 8 | 157469391 | [T/C] |
| M8c157667379 | 8 | 157667379 | [A/G] |
| M8c158117438 | 8 | 158117438 | [A/G] |
| M8c158356334 | 8 | 158356334 | [A/C] |
| M8c158568418 | 8 | 158568418 | [T/G] |
| M8c158967131 | 8 | 158967131 | [A/G] |
| M8c159678152 | 8 | 159678152 | [T/C] |
| M8c159884698 | 8 | 159884698 | [A/G] |
| M8c160237274 | 8 | 160237274 | [T/C] |
| M8c160597909 | 8 | 160597909 | [T/C] |
| M8c161069595 | 8 | 161069595 | [A/G] |
| M8c161523161 | 8 | 161523161 | [T/G] |
| M8c162223926 | 8 | 162223926 | [A/G] |
| M8c162511070 | 8 | 162511070 | [T/C] |
| M8c162756046 | 8 | 162756046 | [A/C] |
| M8c163113635 | 8 | 163113635 | [T/G] |
| M8c163654083 | 8 | 163654083 | [A/G] |
| M8c163905839 | 8 | 163905839 | [T/G] |
| M8c164082982 | 8 | 164082982 | [T/G] |
| M8c164764024 | 8 | 164764024 | [A/G] |
| M8c165280650 | 8 | 165280650 | [T/C] |
| M8c165633914 | 8 | 165633914 | [A/C] |
| M8c165725512 | 8 | 165725512 | [A/G] |
| M8c166080418 | 8 | 166080418 | [T/C] |
| M8c166415625 | 8 | 166415625 | [T/G] |
| M8c167087028 | 8 | 167087028 | [T/C] |
| M8c167154560 | 8 | 167154560 | [A/C] |
| M8c167774380 | 8 | 167774380 | [A/G] |
| M8c168076907 | 8 | 168076907 | [T/C] |
| M8c168272512 | 8 | 168272512 | [T/C] |
| M8c168480084 | 8 | 168480084 | [A/G] |
| M8c169138120 | 8 | 169138120 | [T/C] |
| M8c169781805 | 8 | 169781805 | [T/C] |
| M8c169912932 | 8 | 169912932 | [A/G] |
| M8c170062609 | 8 | 170062609 | [T/G] |
| M8c170434394 | 8 | 170434394 | [T/G] |
| M8c170434652 | 8 | 170434652 | [A/C] |
| M8c170626219 | 8 | 170626219 | [T/C] |
| M8c170861946 | 8 | 170861946 | [A/C] |
| M8c171170184 | 8 | 171170184 | [T/C] |
| M8c171190391 | 8 | 171190391 | [T/C] |
| M8c171439446 | 8 | 171439446 | [T/G] |
| M8c171594852 | 8 | 171594852 | [T/C] |
| M8c171761349 | 8 | 171761349 | [T/C] |
| M8c171894293 | 8 | 171894293 | [T/C] |
| M8c172127404 | 8 | 172127404 | [T/G] |
| M8c173057507 | 8 | 173057507 | [A/G] |
| M8c173081211 | 8 | 173081211 | [T/C] |
| M8c173343649 | 8 | 173343649 | [A/G] |
| M8c173599140 | 8 | 173599140 | [T/C] |
| M8c173654602 | 8 | 173654602 | [T/C] |
| M8c174288029 | 8 | 174288029 | [T/C] |
| M8c174910874 | 8 | 174910874 | [A/G] |
| M8c175068297 | 8 | 175068297 | [A/G] |
| M8c175361260 | 8 | 175361260 | [A/C] |
| M9c67801 | 9 | 67801 | [T/C] |
| M9c479709 | 9 | 479709 | [T/C] |
| M9c562510 | 9 | 562510 | [T/C] |
| M9c1095354 | 9 | 1095354 | [A/G] |
| M9c1347226 | 9 | 1347226 | [A/G] |
| M9c1745799 | 9 | 1745799 | [T/G] |
| M9c2119994 | 9 | 2119994 | [A/G] |
| M9c2485142 | 9 | 2485142 | [T/G] |
| M9c2831674 | 9 | 2831674 | [T/C] |
| M9c3279494 | 9 | 3279494 | [T/G] |
| M9c3899397 | 9 | 3899397 | [A/G] |
| M9c4335560 | 9 | 4335560 | [T/C] |
| M9c4841096 | 9 | 4841096 | [T/C] |
| M9c5077412 | 9 | 5077412 | [T/G] |
| M9c5654837 | 9 | 5654837 | [T/G] |
| M9c5997452 | 9 | 5997452 | [A/G] |
| M9c6053129 | 9 | 6053129 | [A/C] |
| M9c6573303 | 9 | 6573303 | [T/C] |
| M9c7308398 | 9 | 7308398 | [T/C] |
| M9c7723691 | 9 | 7723691 | [A/G] |
| M9c7959566 | 9 | 7959566 | [A/G] |
| M9c8414792 | 9 | 8414792 | [A/G] |
| M9c8779931 | 9 | 8779931 | [T/G] |
| M9c9357565 | 9 | 9357565 | [A/G] |
| M9c9722701 | 9 | 9722701 | [T/C] |
| M9c10012037 | 9 | 10012037 | [T/C] |
| M9c10803367 | 9 | 10803367 | [T/C] |
| M9c11076455 | 9 | 11076455 | [A/G] |
| M9c11398040 | 9 | 11398040 | [A/G] |
| M9c11711229 | 9 | 11711229 | [A/G] |
| M9c12023808 | 9 | 12023808 | [T/C] |
| M9c12795758 | 9 | 12795758 | [A/C] |
| M9c13150872 | 9 | 13150872 | [A/C] |
| M9c13433384 | 9 | 13433384 | [A/G] |
| M9c13912204 | 9 | 13912204 | [A/C] |
| M9c14538449 | 9 | 14538449 | [T/G] |
| M9c14907658 | 9 | 14907658 | [A/G] |
| M9c15163450 | 9 | 15163450 | [T/C] |
| M9c15372004 | 9 | 15372004 | [A/G] |
| M9c16182590 | 9 | 16182590 | [T/C] |
| M9c16682333 | 9 | 16682333 | [T/G] |
| M9c16718980 | 9 | 16718980 | [T/C] |
| M9c17315561 | 9 | 17315561 | [A/G] |
| M9c17397845 | 9 | 17397845 | [A/G] |
| M9c17724716 | 9 | 17724716 | [T/G] |
| M9c18318460 | 9 | 18318460 | [A/G] |
| M9c18684300 | 9 | 18684300 | [A/G] |
| M9c18781600 | 9 | 18781600 | [T/C] |
| M9c19232299 | 9 | 19232299 | [T/C] |
| M9c19533883 | 9 | 19533883 | [T/C] |
| M9c19805727 | 9 | 19805727 | [T/C] |
| M9c20693891 | 9 | 20693891 | [A/C] |
| M9c20969816 | 9 | 20969816 | [T/C] |
| M9c21425349 | 9 | 21425349 | [A/G] |
| M9c21727188 | 9 | 21727188 | [A/G] |
| M9c22102983 | 9 | 22102983 | [T/C] |
| M9c22480651 | 9 | 22480651 | [T/C] |
| M9c22655765 | 9 | 22655765 | [A/G] |
| M9c23754492 | 9 | 23754492 | [T/C] |
| M9c24068782 | 9 | 24068782 | [T/C] |
| M9c24482626 | 9 | 24482626 | [A/G] |
| M9c25040208 | 9 | 25040208 | [A/C] |
| M9c25522467 | 9 | 25522467 | [A/C] |
| M9c25819181 | 9 | 25819181 | [A/G] |
| M9c26187805 | 9 | 26187805 | [A/C] |
| M9c26603259 | 9 | 26603259 | [T/C] |
| M9c26821670 | 9 | 26821670 | [T/C] |
| M9c27222601 | 9 | 27222601 | [T/C] |
| M9c27434238 | 9 | 27434238 | [A/G] |
| M9c28004374 | 9 | 28004374 | [T/C] |
| M9c28007620 | 9 | 28007620 | [A/C] |
| M9c28508618 | 9 | 28508618 | [T/C] |
| M9c29155789 | 9 | 29155789 | [A/G] |
| M9c29403622 | 9 | 29403622 | [T/G] |
| M9c30051161 | 9 | 30051161 | [A/G] |
| M9c30075031 | 9 | 30075031 | [T/C] |
| M9c30644791 | 9 | 30644791 | [T/C] |
| M9c31206633 | 9 | 31206633 | [T/G] |
| M9c31698317 | 9 | 31698317 | [T/C] |
| M9c31907526 | 9 | 31907526 | [A/G] |
| M9c32237338 | 9 | 32237338 | [T/C] |
| M9c32579148 | 9 | 32579148 | [T/C] |
| M9c33138320 | 9 | 33138320 | [T/C] |
| M9c33371828 | 9 | 33371828 | [A/G] |
| M9c33840125 | 9 | 33840125 | [A/C] |
| M9c34242075 | 9 | 34242075 | [T/C] |
| M9c34629499 | 9 | 34629499 | [T/C] |
| M9c34889507 | 9 | 34889507 | [T/C] |
| M9c35447668 | 9 | 35447668 | [T/G] |
| M9c36005376 | 9 | 36005376 | [A/G] |
| M9c36546632 | 9 | 36546632 | [A/C] |
| M9c36849805 | 9 | 36849805 | [T/C] |
| M9c37264484 | 9 | 37264484 | [T/G] |
| M9c37410968 | 9 | 37410968 | [A/G] |
| M9c37700467 | 9 | 37700467 | [T/C] |
| M9c38283941 | 9 | 38283941 | [A/G] |
| M9c38919626 | 9 | 38919626 | [T/C] |
| M9c39001186 | 9 | 39001186 | [T/C] |
| M9c39392879 | 9 | 39392879 | [A/G] |
| M9c39674336 | 9 | 39674336 | [T/C] |
| M9c40205505 | 9 | 40205505 | [A/C] |
| M9c40581446 | 9 | 40581446 | [T/C] |
| M9c40808030 | 9 | 40808030 | [T/C] |
| M9c41353748 | 9 | 41353748 | [T/G] |
| M9c41673533 | 9 | 41673533 | [A/G] |
| M9c41754591 | 9 | 41754591 | [A/G] |
| M9c42118057 | 9 | 42118057 | [A/G] |
| M9c42524405 | 9 | 42524405 | [A/G] |
| M9c42838459 | 9 | 42838459 | [T/C] |
| M9c43241000 | 9 | 43241000 | [T/G] |
| M9c43680761 | 9 | 43680761 | [A/G] |
| M9c43930463 | 9 | 43930463 | [T/C] |
| M9c44379755 | 9 | 44379755 | [A/G] |
| M9c44931092 | 9 | 44931092 | [T/C] |
| M9c45390040 | 9 | 45390040 | [A/G] |
| M9c46065493 | 9 | 46065493 | [A/G] |
| M9c46431485 | 9 | 46431485 | [A/G] |
| M9c46651928 | 9 | 46651928 | [T/C] |
| M9c47213328 | 9 | 47213328 | [T/C] |
| M9c47448529 | 9 | 47448529 | [A/C] |
| M9c48441449 | 9 | 48441449 | [A/G] |
| M9c48738988 | 9 | 48738988 | [T/C] |
| M9c49189690 | 9 | 49189690 | [A/C] |
| M9c50412095 | 9 | 50412095 | [T/C] |
| M9c50717118 | 9 | 50717118 | [T/C] |
| M9c51043623 | 9 | 51043623 | [T/G] |
| M9c51341287 | 9 | 51341287 | [A/G] |
| M9c52397826 | 9 | 52397826 | [A/G] |
| M9c52838588 | 9 | 52838588 | [T/G] |
| M9c53125112 | 9 | 53125112 | [A/G] |
| M9c53953823 | 9 | 53953823 | [T/G] |
| M9c54180421 | 9 | 54180421 | [T/C] |
| M9c54430538 | 9 | 54430538 | [T/C] |
| M9c55094123 | 9 | 55094123 | [A/G] |
| M9c55513159 | 9 | 55513159 | [T/C] |
| M9c55864006 | 9 | 55864006 | [A/G] |
| M9c56422962 | 9 | 56422962 | [T/C] |
| M9c56930897 | 9 | 56930897 | [A/C] |
| M9c57351216 | 9 | 57351216 | [T/C] |
| M9c57848940 | 9 | 57848940 | [A/G] |
| M9c58143137 | 9 | 58143137 | [T/C] |
| M9c59155807 | 9 | 59155807 | [T/C] |
| M9c59402186 | 9 | 59402186 | [T/C] |
| M9c59940232 | 9 | 59940232 | [A/C] |
| M9c60452120 | 9 | 60452120 | [T/G] |
| M9c60761924 | 9 | 60761924 | [T/C] |
| M9c60948993 | 9 | 60948993 | [T/G] |
| M9c62258038 | 9 | 62258038 | [A/C] |
| M9c62377329 | 9 | 62377329 | [A/G] |
| M9c62750164 | 9 | 62750164 | [A/G] |
| M9c63302657 | 9 | 63302657 | [T/C] |
| M9c63556073 | 9 | 63556073 | [T/C] |
| M9c64030069 | 9 | 64030069 | [T/C] |
| M9c64472734 | 9 | 64472734 | [T/C] |
| M9c64968050 | 9 | 64968050 | [T/C] |
| M9c65284468 | 9 | 65284468 | [T/G] |
| M9c65523692 | 9 | 65523692 | [T/G] |
| M9c65962177 | 9 | 65962177 | [A/C] |
| M9c66405185 | 9 | 66405185 | [A/G] |
| M9c66701060 | 9 | 66701060 | [T/C] |
| M9c66906485 | 9 | 66906485 | [T/C] |
| M9c67244120 | 9 | 67244120 | [A/G] |
| M9c67488695 | 9 | 67488695 | [A/C] |
| M9c68025200 | 9 | 68025200 | [T/C] |
| M9c69117140 | 9 | 69117140 | [A/G] |
| M9c69215031 | 9 | 69215031 | [T/G] |
| M9c69569795 | 9 | 69569795 | [T/G] |
| M9c69868619 | 9 | 69868619 | [A/G] |
| M9c70245054 | 9 | 70245054 | [A/C] |
| M9c70793981 | 9 | 70793981 | [T/C] |
| M9c71812082 | 9 | 71812082 | [A/G] |
| M9c71986325 | 9 | 71986325 | [T/C] |
| M9c72920085 | 9 | 72920085 | [A/G] |
| M9c73229468 | 9 | 73229468 | [A/G] |
| M9c73474920 | 9 | 73474920 | [A/G] |
| M9c73710638 | 9 | 73710638 | [T/C] |
| M9c74171133 | 9 | 74171133 | [T/G] |
| M9c74582911 | 9 | 74582911 | [T/C] |
| M9c74694345 | 9 | 74694345 | [A/C] |
| M9c75059600 | 9 | 75059600 | [A/G] |
| M9c75647630 | 9 | 75647630 | [A/G] |
| M9c75779172 | 9 | 75779172 | [A/G] |
| M9c76348680 | 9 | 76348680 | [T/C] |
| M9c76537100 | 9 | 76537100 | [A/G] |
| M9c77026331 | 9 | 77026331 | [T/G] |
| M9c77311403 | 9 | 77311403 | [T/C] |
| M9c77699484 | 9 | 77699484 | [T/C] |
| M9c77907897 | 9 | 77907897 | [T/C] |
| M9c78336287 | 9 | 78336287 | [T/C] |
| M9c78740101 | 9 | 78740101 | [A/G] |
| M9c78815951 | 9 | 78815951 | [A/C] |
| M9c79414309 | 9 | 79414309 | [A/C] |
| M9c80489821 | 9 | 80489821 | [T/G] |
| M9c80810491 | 9 | 80810491 | [A/G] |
| M9c81160787 | 9 | 81160787 | [A/G] |
| M9c81299894 | 9 | 81299894 | [A/G] |
| M9c81656013 | 9 | 81656013 | [T/C] |
| M9c82088725 | 9 | 82088725 | [T/C] |
| M9c82284479 | 9 | 82284479 | [T/C] |
| M9c82817706 | 9 | 82817706 | [T/C] |
| M9c83797540 | 9 | 83797540 | [A/G] |
| M9c84224486 | 9 | 84224486 | [T/C] |
| M9c84871908 | 9 | 84871908 | [A/G] |
| M9c85232157 | 9 | 85232157 | [A/G] |
| M9c85570556 | 9 | 85570556 | [T/G] |
| M9c85953859 | 9 | 85953859 | [T/C] |
| M9c86325747 | 9 | 86325747 | [A/G] |
| M9c86405934 | 9 | 86405934 | [T/C] |
| M9c86866892 | 9 | 86866892 | [T/G] |
| M9c87257534 | 9 | 87257534 | [T/C] |
| M9c87565972 | 9 | 87565972 | [A/C] |
| M9c87942669 | 9 | 87942669 | [A/G] |
| M9c88589046 | 9 | 88589046 | [T/G] |
| M9c88926836 | 9 | 88926836 | [A/C] |
| M9c89332838 | 9 | 89332838 | [A/G] |
| M9c89706634 | 9 | 89706634 | [A/G] |
| M9c90366747 | 9 | 90366747 | [A/G] |
| M9c90766028 | 9 | 90766028 | [A/G] |
| M9c90837517 | 9 | 90837517 | [T/C] |
| M9c91791404 | 9 | 91791404 | [A/G] |
| M9c92014109 | 9 | 92014109 | [T/C] |
| M9c92340446 | 9 | 92340446 | [A/G] |
| M9c92664752 | 9 | 92664752 | [T/C] |
| M9c92870076 | 9 | 92870076 | [T/C] |
| M9c93812403 | 9 | 93812403 | [A/G] |
| M9c94005573 | 9 | 94005573 | [A/G] |
| M9c94254261 | 9 | 94254261 | [T/C] |
| M9c94805463 | 9 | 94805463 | [T/G] |
| M9c95129323 | 9 | 95129323 | [T/G] |
| M9c95443467 | 9 | 95443467 | [A/G] |
| M9c96001102 | 9 | 96001102 | [T/C] |
| M9c96523862 | 9 | 96523862 | [T/C] |
| M9c96704836 | 9 | 96704836 | [A/G] |
| M9c97283498 | 9 | 97283498 | [T/C] |
| M9c97392843 | 9 | 97392843 | [A/G] |
| M9c98281761 | 9 | 98281761 | [T/C] |
| M9c98435374 | 9 | 98435374 | [A/G] |
| M9c99293080 | 9 | 99293080 | [A/C] |
| M9c99440783 | 9 | 99440783 | [T/C] |
| M9c99863225 | 9 | 99863225 | [T/C] |
| M9c100304876 | 9 | 100304876 | [A/C] |
| M9c100421628 | 9 | 100421628 | [A/G] |
| M9c101271301 | 9 | 101271301 | [T/C] |
| M9c101751787 | 9 | 101751787 | [A/C] |
| M9c101901924 | 9 | 101901924 | [T/C] |
| M9c102354287 | 9 | 102354287 | [T/C] |
| M9c102787943 | 9 | 102787943 | [A/C] |
| M9c102962681 | 9 | 102962681 | [T/C] |
| M9c103351448 | 9 | 103351448 | [A/G] |
| M9c103689861 | 9 | 103689861 | [T/G] |
| M9c104031858 | 9 | 104031858 | [A/C] |
| M9c104485867 | 9 | 104485867 | [A/G] |
| M9c104590128 | 9 | 104590128 | [A/G] |
| M9c105254865 | 9 | 105254865 | [A/C] |
| M9c105691846 | 9 | 105691846 | [A/C] |
| M9c106224632 | 9 | 106224632 | [A/G] |
| M9c106407250 | 9 | 106407250 | [A/G] |
| M9c106621408 | 9 | 106621408 | [A/G] |
| M9c107418847 | 9 | 107418847 | [T/G] |
| M9c107627382 | 9 | 107627382 | [T/C] |
| M9c108206665 | 9 | 108206665 | [T/C] |
| M9c108658005 | 9 | 108658005 | [A/C] |
| M9c109124619 | 9 | 109124619 | [T/C] |
| M9c109546729 | 9 | 109546729 | [T/C] |
| M9c109887072 | 9 | 109887072 | [T/G] |
| M9c111117374 | 9 | 111117374 | [T/C] |
| M9c111606512 | 9 | 111606512 | [T/C] |
| M9c112007544 | 9 | 112007544 | [T/C] |
| M9c112249891 | 9 | 112249891 | [T/C] |
| M9c112487354 | 9 | 112487354 | [A/G] |
| M9c112882741 | 9 | 112882741 | [A/G] |
| M9c113421280 | 9 | 113421280 | [T/C] |
| M9c113706194 | 9 | 113706194 | [T/C] |
| M9c113787513 | 9 | 113787513 | [T/C] |
| M9c114176736 | 9 | 114176736 | [T/G] |
| M9c114551765 | 9 | 114551765 | [A/G] |
| M9c114920512 | 9 | 114920512 | [T/G] |
| M9c115378545 | 9 | 115378545 | [T/G] |
| M9c115496986 | 9 | 115496986 | [T/C] |
| M9c115887355 | 9 | 115887355 | [A/G] |
| M9c116751615 | 9 | 116751615 | [T/G] |
| M9c116954765 | 9 | 116954765 | [A/G] |
| M9c117330059 | 9 | 117330059 | [T/G] |
| M9c117825973 | 9 | 117825973 | [T/C] |
| M9c118179084 | 9 | 118179084 | [T/C] |
| M9c118258419 | 9 | 118258419 | [A/G] |
| M9c118864323 | 9 | 118864323 | [A/G] |
| M9c118940679 | 9 | 118940679 | [T/C] |
| M9c119863019 | 9 | 119863019 | [T/C] |
| M9c120881924 | 9 | 120881924 | [T/C] |
| M9c121170553 | 9 | 121170553 | [T/C] |
| M9c121589717 | 9 | 121589717 | [A/G] |
| M9c121934740 | 9 | 121934740 | [T/C] |
| M9c122225918 | 9 | 122225918 | [A/G] |
| M9c122516842 | 9 | 122516842 | [A/G] |
| M9c122827261 | 9 | 122827261 | [T/G] |
| M9c123239782 | 9 | 123239782 | [A/G] |
| M9c123408778 | 9 | 123408778 | [T/G] |
| M9c123410637 | 9 | 123410637 | [T/C] |
| M9c124235889 | 9 | 124235889 | [T/C] |
| M9c124567952 | 9 | 124567952 | [A/C] |
| M9c125128384 | 9 | 125128384 | [A/G] |
| M9c125688807 | 9 | 125688807 | [A/G] |
| M9c125895099 | 9 | 125895099 | [T/C] |
| M9c126340595 | 9 | 126340595 | [A/G] |
| M9c126771620 | 9 | 126771620 | [A/C] |
| M9c127002672 | 9 | 127002672 | [T/C] |
| M9c127216479 | 9 | 127216479 | [T/C] |
| M9c127521836 | 9 | 127521836 | [T/C] |
| M9c128120205 | 9 | 128120205 | [A/G] |
| M9c128120585 | 9 | 128120585 | [T/C] |
| M9c128316291 | 9 | 128316291 | [A/G] |
| M9c128726943 | 9 | 128726943 | [T/C] |
| M9c129006784 | 9 | 129006784 | [A/G] |
| M9c129277973 | 9 | 129277973 | [A/G] |
| M9c129766757 | 9 | 129766757 | [A/C] |
| M9c130241408 | 9 | 130241408 | [T/C] |
| M9c131584697 | 9 | 131584697 | [T/C] |
| M9c131914243 | 9 | 131914243 | [A/G] |
| M9c132033320 | 9 | 132033320 | [T/C] |
| M9c132551091 | 9 | 132551091 | [T/G] |
| M9c133586192 | 9 | 133586192 | [A/G] |
| M9c133907668 | 9 | 133907668 | [A/G] |
| M9c134290305 | 9 | 134290305 | [T/G] |
| M9c134545759 | 9 | 134545759 | [A/G] |
| M9c135270601 | 9 | 135270601 | [T/C] |
| M9c135459610 | 9 | 135459610 | [A/G] |
| M9c135877273 | 9 | 135877273 | [A/G] |
| M9c136392759 | 9 | 136392759 | [T/C] |
| M9c136616403 | 9 | 136616403 | [T/C] |
| M9c136868603 | 9 | 136868603 | [A/G] |
| M9c137763592 | 9 | 137763592 | [T/C] |
| M9c138243599 | 9 | 138243599 | [T/C] |
| M9c138321109 | 9 | 138321109 | [T/G] |
| M9c138888612 | 9 | 138888612 | [A/G] |
| M9c139383322 | 9 | 139383322 | [A/G] |
| M9c139516425 | 9 | 139516425 | [T/C] |
| M9c140346496 | 9 | 140346496 | [A/G] |
| M9c140945744 | 9 | 140945744 | [T/C] |
| M9c141334638 | 9 | 141334638 | [A/C] |
| M9c141847024 | 9 | 141847024 | [T/C] |
| M9c142205835 | 9 | 142205835 | [A/C] |
| M9c142293132 | 9 | 142293132 | [A/G] |
| M9c142807662 | 9 | 142807662 | [T/C] |
| M9c143353438 | 9 | 143353438 | [A/G] |
| M9c144308202 | 9 | 144308202 | [T/C] |
| M9c144798156 | 9 | 144798156 | [T/C] |
| M9c145062776 | 9 | 145062776 | [A/G] |
| M9c145528742 | 9 | 145528742 | [A/G] |
| M9c145880632 | 9 | 145880632 | [T/G] |
| M9c146228335 | 9 | 146228335 | [A/G] |
| M9c146458765 | 9 | 146458765 | [A/C] |
| M9c146872957 | 9 | 146872957 | [T/G] |
| M9c147056175 | 9 | 147056175 | [T/C] |
| M9c147371952 | 9 | 147371952 | [T/C] |
| M9c147491750 | 9 | 147491750 | [T/C] |
| M9c147640619 | 9 | 147640619 | [T/C] |
| M9c147801511 | 9 | 147801511 | [T/C] |
| M9c147848282 | 9 | 147848282 | [A/G] |
| M9c148365188 | 9 | 148365188 | [T/C] |
| M9c148668335 | 9 | 148668335 | [A/C] |
| M9c149041431 | 9 | 149041431 | [T/C] |
| M9c149277381 | 9 | 149277381 | [A/G] |
| M9c149647935 | 9 | 149647935 | [A/C] |
| M9c149990631 | 9 | 149990631 | [A/G] |
| M9c150237694 | 9 | 150237694 | [T/C] |
| M9c150716195 | 9 | 150716195 | [A/G] |
| M9c151277505 | 9 | 151277505 | [A/C] |
| M9c151683547 | 9 | 151683547 | [T/C] |
| M9c152049500 | 9 | 152049500 | [A/G] |
| M9c152461145 | 9 | 152461145 | [A/G] |
| M9c152858889 | 9 | 152858889 | [T/C] |
| M9c153035514 | 9 | 153035514 | [T/C] |
| M9c153037207 | 9 | 153037207 | [A/G] |
| M9c153737190 | 9 | 153737190 | [T/G] |
| M9c153852495 | 9 | 153852495 | [T/G] |
| M9c153918177 | 9 | 153918177 | [T/C] |
| M9c153942107 | 9 | 153942107 | [A/G] |
| M9c154247743 | 9 | 154247743 | [T/G] |
| M9c154780829 | 9 | 154780829 | [T/C] |
| M9c155285404 | 9 | 155285404 | [T/G] |
| M9c155569996 | 9 | 155569996 | [T/G] |
| M9c155766253 | 9 | 155766253 | [T/C] |
| M9c156200679 | 9 | 156200679 | [A/G] |
| M10c635478 | 10 | 635478 | [T/C] |
| M10c1146832 | 10 | 1146832 | [A/C] |
| M10c1540929 | 10 | 1540929 | [T/C] |
| M10c1718544 | 10 | 1718544 | [T/G] |
| M10c2040152 | 10 | 2040152 | [T/G] |
| M10c2460975 | 10 | 2460975 | [T/C] |
| M10c2987731 | 10 | 2987731 | [T/C] |
| M10c3273852 | 10 | 3273852 | [A/G] |
| M10c4018308 | 10 | 4018308 | [A/C] |
| M10c4059265 | 10 | 4059265 | [T/G] |
| M10c4592159 | 10 | 4592159 | [T/C] |
| M10c4749661 | 10 | 4749661 | [A/G] |
| M10c5874720 | 10 | 5874720 | [A/G] |
| M10c6352259 | 10 | 6352259 | [A/G] |
| M10c6678670 | 10 | 6678670 | [A/C] |
| M10c7107284 | 10 | 7107284 | [T/C] |
| M10c7400953 | 10 | 7400953 | [T/C] |
| M10c7471175 | 10 | 7471175 | [T/C] |
| M10c7869104 | 10 | 7869104 | [A/G] |
| M10c8276909 | 10 | 8276909 | [T/C] |
| M10c9077274 | 10 | 9077274 | [T/C] |
| M10c9473036 | 10 | 9473036 | [A/G] |
| M10c9540329 | 10 | 9540329 | [T/C] |
| M10c10136715 | 10 | 10136715 | [T/C] |
| M10c10284160 | 10 | 10284160 | [A/G] |
| M10c10605596 | 10 | 10605596 | [T/G] |
| M10c11195560 | 10 | 11195560 | [A/C] |
| M10c11404170 | 10 | 11404170 | [T/C] |
| M10c11904525 | 10 | 11904525 | [A/G] |
| M10c12349291 | 10 | 12349291 | [T/C] |
| M10c12925342 | 10 | 12925342 | [A/G] |
| M10c13162543 | 10 | 13162543 | [T/G] |
| M10c13554464 | 10 | 13554464 | [T/C] |
| M10c13784062 | 10 | 13784062 | [T/C] |
| M10c14084690 | 10 | 14084690 | [T/C] |
| M10c14446076 | 10 | 14446076 | [A/C] |
| M10c14976129 | 10 | 14976129 | [T/C] |
| M10c15224313 | 10 | 15224313 | [T/C] |
| M10c15395362 | 10 | 15395362 | [A/G] |
| M10c15717562 | 10 | 15717562 | [T/C] |
| M10c16360875 | 10 | 16360875 | [A/G] |
| M10c16504699 | 10 | 16504699 | [T/C] |
| M10c16867515 | 10 | 16867515 | [T/C] |
| M10c17679150 | 10 | 17679150 | [T/G] |
| M10c17887527 | 10 | 17887527 | [T/C] |
| M10c18125785 | 10 | 18125785 | [A/C] |
| M10c18666706 | 10 | 18666706 | [A/G] |
| M10c18863119 | 10 | 18863119 | [T/C] |
| M10c19237891 | 10 | 19237891 | [A/G] |
| M10c19480108 | 10 | 19480108 | [A/G] |
| M10c19851929 | 10 | 19851929 | [T/G] |
| M10c20384013 | 10 | 20384013 | [A/G] |
| M10c21019080 | 10 | 21019080 | [T/C] |
| M10c21499887 | 10 | 21499887 | [T/G] |
| M10c21823409 | 10 | 21823409 | [A/C] |
| M10c22092917 | 10 | 22092917 | [A/G] |
| M10c22365430 | 10 | 22365430 | [T/C] |
| M10c22754552 | 10 | 22754552 | [A/G] |
| M10c22973239 | 10 | 22973239 | [T/G] |
| M10c23491162 | 10 | 23491162 | [T/C] |
| M10c24209871 | 10 | 24209871 | [A/G] |
| M10c24444109 | 10 | 24444109 | [A/G] |
| M10c24651119 | 10 | 24651119 | [T/C] |
| M10c25300847 | 10 | 25300847 | [T/G] |
| M10c25555847 | 10 | 25555847 | [T/C] |
| M10c25775006 | 10 | 25775006 | [A/G] |
| M10c26135086 | 10 | 26135086 | [T/C] |
| M10c26357897 | 10 | 26357897 | [A/G] |
| M10c26985847 | 10 | 26985847 | [T/C] |
| M10c27318222 | 10 | 27318222 | [A/G] |
| M10c27715037 | 10 | 27715037 | [T/G] |
| M10c28644890 | 10 | 28644890 | [T/C] |
| M10c28772088 | 10 | 28772088 | [A/G] |
| M10c29535932 | 10 | 29535932 | [A/C] |
| M10c29884487 | 10 | 29884487 | [T/C] |
| M10c30191169 | 10 | 30191169 | [T/C] |
| M10c30657383 | 10 | 30657383 | [T/C] |
| M10c30862631 | 10 | 30862631 | [A/G] |
| M10c31309836 | 10 | 31309836 | [T/C] |
| M10c31723614 | 10 | 31723614 | [T/C] |
| M10c32142080 | 10 | 32142080 | [A/G] |
| M10c32194971 | 10 | 32194971 | [T/G] |
| M10c32531836 | 10 | 32531836 | [A/G] |
| M10c33165305 | 10 | 33165305 | [A/G] |
| M10c33348977 | 10 | 33348977 | [A/C] |
| M10c33744550 | 10 | 33744550 | [A/G] |
| M10c34151685 | 10 | 34151685 | [T/G] |
| M10c34502588 | 10 | 34502588 | [T/C] |
| M10c34913111 | 10 | 34913111 | [A/G] |
| M10c34930756 | 10 | 34930756 | [A/G] |
| M10c35534387 | 10 | 35534387 | [T/C] |
| M10c35800647 | 10 | 35800647 | [T/C] |
| M10c36029908 | 10 | 36029908 | [A/G] |
| M10c36289827 | 10 | 36289827 | [T/C] |
| M10c36779715 | 10 | 36779715 | [T/C] |
| M10c37625748 | 10 | 37625748 | [T/C] |
| M10c37909172 | 10 | 37909172 | [A/G] |
| M10c38375228 | 10 | 38375228 | [A/C] |
| M10c38918622 | 10 | 38918622 | [A/G] |
| M10c39185214 | 10 | 39185214 | [A/C] |
| M10c39590245 | 10 | 39590245 | [A/G] |
| M10c39952669 | 10 | 39952669 | [T/C] |
| M10c40330446 | 10 | 40330446 | [T/C] |
| M10c40590217 | 10 | 40590217 | [A/G] |
| M10c40952768 | 10 | 40952768 | [T/C] |
| M10c41284095 | 10 | 41284095 | [T/G] |
| M10c41656152 | 10 | 41656152 | [A/C] |
| M10c42035858 | 10 | 42035858 | [T/C] |
| M10c42246352 | 10 | 42246352 | [A/G] |
| M10c42593372 | 10 | 42593372 | [T/C] |
| M10c42884089 | 10 | 42884089 | [A/G] |
| M10c43331623 | 10 | 43331623 | [T/C] |
| M10c43589689 | 10 | 43589689 | [T/C] |
| M10c44004360 | 10 | 44004360 | [A/G] |
| M10c44516060 | 10 | 44516060 | [A/G] |
| M10c44731485 | 10 | 44731485 | [T/C] |
| M10c45030785 | 10 | 45030785 | [A/G] |
| M10c45333350 | 10 | 45333350 | [A/G] |
| M10c45704273 | 10 | 45704273 | [T/G] |
| M10c46060173 | 10 | 46060173 | [A/G] |
| M10c46267003 | 10 | 46267003 | [A/G] |
| M10c46874843 | 10 | 46874843 | [T/G] |
| M10c47013045 | 10 | 47013045 | [T/G] |
| M10c47751394 | 10 | 47751394 | [A/G] |
| M10c48602604 | 10 | 48602604 | [T/G] |
| M10c49273075 | 10 | 49273075 | [A/G] |
| M10c49375319 | 10 | 49375319 | [T/C] |
| M10c49719461 | 10 | 49719461 | [T/C] |
| M10c50476952 | 10 | 50476952 | [T/C] |
| M10c51436407 | 10 | 51436407 | [A/G] |
| M10c52208064 | 10 | 52208064 | [A/G] |
| M10c53039030 | 10 | 53039030 | [T/C] |
| M10c53109492 | 10 | 53109492 | [T/C] |
| M10c53538592 | 10 | 53538592 | [T/C] |
| M10c53789928 | 10 | 53789928 | [A/G] |
| M10c54432827 | 10 | 54432827 | [A/G] |
| M10c54936447 | 10 | 54936447 | [A/C] |
| M10c55465011 | 10 | 55465011 | [A/G] |
| M10c55753549 | 10 | 55753549 | [T/G] |
| M10c55989354 | 10 | 55989354 | [A/G] |
| M10c56217696 | 10 | 56217696 | [T/C] |
| M10c56719781 | 10 | 56719781 | [T/G] |
| M10c57118410 | 10 | 57118410 | [T/C] |
| M10c57543894 | 10 | 57543894 | [T/C] |
| M10c57756592 | 10 | 57756592 | [A/G] |
| M10c57991733 | 10 | 57991733 | [T/C] |
| M10c58300463 | 10 | 58300463 | [A/G] |
| M10c59115236 | 10 | 59115236 | [T/C] |
| M10c59545851 | 10 | 59545851 | [T/C] |
| M10c59660713 | 10 | 59660713 | [A/G] |
| M10c60085668 | 10 | 60085668 | [T/C] |
| M10c60480911 | 10 | 60480911 | [T/C] |
| M10c60654872 | 10 | 60654872 | [T/C] |
| M10c61002261 | 10 | 61002261 | [T/G] |
| M10c61382520 | 10 | 61382520 | [T/C] |
| M10c61802184 | 10 | 61802184 | [A/C] |
| M10c62157839 | 10 | 62157839 | [A/G] |
| M10c62371912 | 10 | 62371912 | [T/G] |
| M10c63034738 | 10 | 63034738 | [T/G] |
| M10c63065942 | 10 | 63065942 | [A/G] |
| M10c63400788 | 10 | 63400788 | [A/G] |
| M10c63815517 | 10 | 63815517 | [T/C] |
| M10c64426354 | 10 | 64426354 | [A/G] |
| M10c65341098 | 10 | 65341098 | [A/C] |
| M10c65746892 | 10 | 65746892 | [T/C] |
| M10c66015220 | 10 | 66015220 | [A/C] |
| M10c66311427 | 10 | 66311427 | [A/G] |
| M10c66637833 | 10 | 66637833 | [A/G] |
| M10c67010262 | 10 | 67010262 | [T/C] |
| M10c67808176 | 10 | 67808176 | [T/C] |
| M10c68022174 | 10 | 68022174 | [T/C] |
| M10c68338845 | 10 | 68338845 | [T/C] |
| M10c68725554 | 10 | 68725554 | [A/C] |
| M10c68925257 | 10 | 68925257 | [T/C] |
| M10c69295407 | 10 | 69295407 | [T/G] |
| M10c70278949 | 10 | 70278949 | [T/C] |
| M10c70920639 | 10 | 70920639 | [A/G] |
| M10c71162483 | 10 | 71162483 | [A/G] |
| M10c71463532 | 10 | 71463532 | [T/C] |
| M10c71863846 | 10 | 71863846 | [T/C] |
| M10c72298705 | 10 | 72298705 | [A/G] |
| M10c72621523 | 10 | 72621523 | [T/G] |
| M10c72705964 | 10 | 72705964 | [A/G] |
| M10c73133995 | 10 | 73133995 | [A/C] |
| M10c73510804 | 10 | 73510804 | [A/G] |
| M10c73701000 | 10 | 73701000 | [A/C] |
| M10c74301843 | 10 | 74301843 | [A/C] |
| M10c74518422 | 10 | 74518422 | [A/G] |
| M10c74850456 | 10 | 74850456 | [T/C] |
| M10c75669538 | 10 | 75669538 | [A/G] |
| M10c75814843 | 10 | 75814843 | [T/C] |
| M10c76421781 | 10 | 76421781 | [T/C] |
| M10c77108995 | 10 | 77108995 | [A/C] |
| M10c77677349 | 10 | 77677349 | [T/C] |
| M10c78134503 | 10 | 78134503 | [T/G] |
| M10c78320681 | 10 | 78320681 | [T/C] |
| M10c79135350 | 10 | 79135350 | [A/G] |
| M10c79412969 | 10 | 79412969 | [A/G] |
| M10c79753358 | 10 | 79753358 | [A/G] |
| M10c80053613 | 10 | 80053613 | [T/C] |
| M10c80406216 | 10 | 80406216 | [A/G] |
| M10c80737744 | 10 | 80737744 | [T/C] |
| M10c81019839 | 10 | 81019839 | [T/C] |
| M10c81442611 | 10 | 81442611 | [T/C] |
| M10c82046994 | 10 | 82046994 | [A/G] |
| M10c82356840 | 10 | 82356840 | [T/C] |
| M10c82999293 | 10 | 82999293 | [A/G] |
| M10c83593772 | 10 | 83593772 | [T/C] |
| M10c83672313 | 10 | 83672313 | [T/G] |
| M10c84240546 | 10 | 84240546 | [T/C] |
| M10c84431555 | 10 | 84431555 | [A/G] |
| M10c84760641 | 10 | 84760641 | [T/C] |
| M10c85011513 | 10 | 85011513 | [T/C] |
| M10c85499180 | 10 | 85499180 | [A/C] |
| M10c86101631 | 10 | 86101631 | [A/C] |
| M10c86423321 | 10 | 86423321 | [T/C] |
| M10c86985240 | 10 | 86985240 | [T/C] |
| M10c87084977 | 10 | 87084977 | [A/C] |
| M10c87475404 | 10 | 87475404 | [A/G] |
| M10c87961459 | 10 | 87961459 | [T/C] |
| M10c88199245 | 10 | 88199245 | [A/C] |
| M10c88611489 | 10 | 88611489 | [A/G] |
| M10c88859038 | 10 | 88859038 | [T/C] |
| M10c89437855 | 10 | 89437855 | [T/C] |
| M10c89546659 | 10 | 89546659 | [A/G] |
| M10c89983054 | 10 | 89983054 | [A/G] |
| M10c90221665 | 10 | 90221665 | [A/C] |
| M10c90816809 | 10 | 90816809 | [A/G] |
| M10c90932180 | 10 | 90932180 | [A/G] |
| M10c91480586 | 10 | 91480586 | [A/G] |
| M10c91858693 | 10 | 91858693 | [A/C] |
| M10c92134701 | 10 | 92134701 | [A/G] |
| M10c92537364 | 10 | 92537364 | [T/C] |
| M10c92790950 | 10 | 92790950 | [A/C] |
| M10c93025374 | 10 | 93025374 | [A/G] |
| M10c93396465 | 10 | 93396465 | [T/C] |
| M10c93685769 | 10 | 93685769 | [T/C] |
| M10c94250954 | 10 | 94250954 | [A/G] |
| M10c94591068 | 10 | 94591068 | [A/C] |
| M10c94698918 | 10 | 94698918 | [A/G] |
| M10c95212311 | 10 | 95212311 | [T/C] |
| M10c95914964 | 10 | 95914964 | [T/C] |
| M10c96321785 | 10 | 96321785 | [A/G] |
| M10c96330876 | 10 | 96330876 | [A/G] |
| M10c96759800 | 10 | 96759800 | [T/C] |
| M10c97069503 | 10 | 97069503 | [T/G] |
| M10c97381903 | 10 | 97381903 | [T/C] |
| M10c97726713 | 10 | 97726713 | [A/G] |
| M10c98368399 | 10 | 98368399 | [A/C] |
| M10c98698308 | 10 | 98698308 | [T/C] |
| M10c98998972 | 10 | 98998972 | [A/G] |
| M10c99111257 | 10 | 99111257 | [A/G] |
| M10c99471661 | 10 | 99471661 | [A/G] |
| M10c100028102 | 10 | 100028102 | [A/C] |
| M10c100132980 | 10 | 100132980 | [T/C] |
| M10c101121574 | 10 | 101121574 | [A/C] |
| M10c101933970 | 10 | 101933970 | [A/G] |
| M10c102371360 | 10 | 102371360 | [T/C] |
| M10c102588166 | 10 | 102588166 | [T/G] |
| M10c102870280 | 10 | 102870280 | [A/G] |
| M10c103216473 | 10 | 103216473 | [A/C] |
| M10c103632968 | 10 | 103632968 | [A/G] |
| M10c103961931 | 10 | 103961931 | [T/C] |
| M10c104481509 | 10 | 104481509 | [T/C] |
| M10c104593842 | 10 | 104593842 | [T/C] |
| M10c105299905 | 10 | 105299905 | [A/C] |
| M10c106270266 | 10 | 106270266 | [T/C] |
| M10c106313274 | 10 | 106313274 | [A/G] |
| M10c106842914 | 10 | 106842914 | [T/C] |
| M10c107228342 | 10 | 107228342 | [T/C] |
| M10c107429451 | 10 | 107429451 | [A/C] |
| M10c107811029 | 10 | 107811029 | [A/G] |
| M10c108061474 | 10 | 108061474 | [A/G] |
| M10c108377991 | 10 | 108377991 | [A/G] |
| M10c108999262 | 10 | 108999262 | [T/G] |
| M10c109342990 | 10 | 109342990 | [A/G] |
| M10c109800938 | 10 | 109800938 | [A/G] |
| M10c110199639 | 10 | 110199639 | [T/G] |
| M10c111240117 | 10 | 111240117 | [A/G] |
| M10c111431197 | 10 | 111431197 | [T/G] |
| M10c112093436 | 10 | 112093436 | [T/C] |
| M10c112394357 | 10 | 112394357 | [A/G] |
| M10c112569938 | 10 | 112569938 | [T/C] |
| M10c112939197 | 10 | 112939197 | [A/C] |
| M10c113161797 | 10 | 113161797 | [T/C] |
| M10c113519562 | 10 | 113519562 | [T/C] |
| M10c113831432 | 10 | 113831432 | [T/G] |
| M10c114195601 | 10 | 114195601 | [A/G] |
| M10c114834127 | 10 | 114834127 | [A/C] |
| M10c115149333 | 10 | 115149333 | [T/C] |
| M10c115833537 | 10 | 115833537 | [T/C] |
| M10c116057066 | 10 | 116057066 | [T/G] |
| M10c116507242 | 10 | 116507242 | [A/G] |
| M10c116615216 | 10 | 116615216 | [A/C] |
| M10c116894888 | 10 | 116894888 | [A/G] |
| M10c117025499 | 10 | 117025499 | [T/C] |
| M10c117559336 | 10 | 117559336 | [T/C] |
| M10c117785671 | 10 | 117785671 | [T/C] |
| M10c117991308 | 10 | 117991308 | [A/G] |
| M10c118509465 | 10 | 118509465 | [T/C] |
| M10c119012395 | 10 | 119012395 | [T/C] |
| M10c119414454 | 10 | 119414454 | [A/C] |
| M10c119905598 | 10 | 119905598 | [T/C] |
| M10c120030104 | 10 | 120030104 | [T/C] |
| M10c120648677 | 10 | 120648677 | [T/C] |
| M10c120742822 | 10 | 120742822 | [A/G] |
| M10c121691775 | 10 | 121691775 | [T/G] |
| M10c121724611 | 10 | 121724611 | [A/G] |
| M10c122523048 | 10 | 122523048 | [T/G] |
| M10c122808698 | 10 | 122808698 | [A/G] |
| M10c123231441 | 10 | 123231441 | [T/G] |
| M10c123685470 | 10 | 123685470 | [A/G] |
| M10c123814808 | 10 | 123814808 | [A/C] |
| M10c124191841 | 10 | 124191841 | [A/G] |
| M10c124532265 | 10 | 124532265 | [T/C] |
| M10c125088457 | 10 | 125088457 | [T/G] |
| M10c125195458 | 10 | 125195458 | [T/C] |
| M10c125545225 | 10 | 125545225 | [A/G] |
| M10c126492232 | 10 | 126492232 | [T/C] |
| M10c126532347 | 10 | 126532347 | [A/G] |
| M10c127247334 | 10 | 127247334 | [A/G] |
| M10c127572178 | 10 | 127572178 | [T/G] |
| M10c127994745 | 10 | 127994745 | [A/G] |
| M10c128564779 | 10 | 128564779 | [T/C] |
| M10c128697002 | 10 | 128697002 | [A/C] |
| M10c129252335 | 10 | 129252335 | [T/G] |
| M10c129512689 | 10 | 129512689 | [T/C] |
| M10c129777165 | 10 | 129777165 | [A/G] |
| M10c130034895 | 10 | 130034895 | [A/G] |
| M10c130299291 | 10 | 130299291 | [T/C] |
| M10c131049944 | 10 | 131049944 | [T/C] |
| M10c131571379 | 10 | 131571379 | [A/G] |
| M10c131888304 | 10 | 131888304 | [T/C] |
| M10c132238779 | 10 | 132238779 | [A/G] |
| M10c132532921 | 10 | 132532921 | [A/G] |
| M10c132962221 | 10 | 132962221 | [A/C] |
| M10c133312916 | 10 | 133312916 | [A/C] |
| M10c133710249 | 10 | 133710249 | [A/G] |
| M10c134058374 | 10 | 134058374 | [T/C] |
| M10c134403307 | 10 | 134403307 | [A/G] |
| M10c134566914 | 10 | 134566914 | [A/G] |
| M10c134829043 | 10 | 134829043 | [T/G] |
| M10c135161879 | 10 | 135161879 | [A/C] |
| M10c135609157 | 10 | 135609157 | [T/C] |
| M10c135800785 | 10 | 135800785 | [T/C] |
| M10c136296547 | 10 | 136296547 | [A/G] |
| M10c136713611 | 10 | 136713611 | [T/G] |
| M10c137291157 | 10 | 137291157 | [A/G] |
| M10c137746026 | 10 | 137746026 | [A/G] |
| M10c138086034 | 10 | 138086034 | [T/G] |
| M10c138193098 | 10 | 138193098 | [T/C] |
| M10c139117657 | 10 | 139117657 | [T/C] |
| M10c139317036 | 10 | 139317036 | [T/C] |
| M10c139876850 | 10 | 139876850 | [T/G] |
| M10c140306724 | 10 | 140306724 | [A/G] |
| M10c140740153 | 10 | 140740153 | [A/G] |
| M10c140960766 | 10 | 140960766 | [A/G] |
| M10c141128570 | 10 | 141128570 | [T/C] |
| M10c141356823 | 10 | 141356823 | [A/G] |
| M10c141356830 | 10 | 141356830 | [A/G] |
| M10c141619861 | 10 | 141619861 | [A/G] |
| M10c141942500 | 10 | 141942500 | [T/C] |
| M10c141966035 | 10 | 141966035 | [T/C] |
| M10c141966150 | 10 | 141966150 | [A/G] |
| M10c142598476 | 10 | 142598476 | [A/C] |
| M10c142786371 | 10 | 142786371 | [A/G] |
| M10c143062782 | 10 | 143062782 | [T/C] |
| M10c143378210 | 10 | 143378210 | [T/C] |
| M10c143739002 | 10 | 143739002 | [T/C] |
| M10c144135011 | 10 | 144135011 | [T/C] |
| M10c144445966 | 10 | 144445966 | [A/G] |
| M10c145194874 | 10 | 145194874 | [A/G] |
| M10c145464991 | 10 | 145464991 | [T/C] |
| M10c145934793 | 10 | 145934793 | [T/C] |
| M10c146183778 | 10 | 146183778 | [T/C] |
| M10c146467622 | 10 | 146467622 | [A/G] |
| M10c147000806 | 10 | 147000806 | [A/G] |
| M10c147012224 | 10 | 147012224 | [T/G] |
| M10c147134721 | 10 | 147134721 | [T/G] |
| M10c147152045 | 10 | 147152045 | [A/C] |
| M10c147330962 | 10 | 147330962 | [A/C] |
| M10c147594223 | 10 | 147594223 | [A/C] |
| M10c147608204 | 10 | 147608204 | [A/C] |
| M10c147845141 | 10 | 147845141 | [A/G] |
| M10c147845162 | 10 | 147845162 | [T/C] |
| M10c148514463 | 10 | 148514463 | [A/G] |
| M10c149029569 | 10 | 149029569 | [T/C] |
| M10c149597169 | 10 | 149597169 | [A/G] |
| M10c150077187 | 10 | 150077187 | [A/G] |

**Supplementary Table S4. The ten linkage groups for three genetic linkage maps of 220 × PH4CV, 220 × Y1518 and P9-10 × PH4CV, and a consensus linkage map.**

|  | Chromosomes | Chr1 | Chr2 | Chr3 | Chr4 | Chr5 | Chr6 | Chr7 | Chr8 | Chr9 | Chr10 | Total |
| --- | --- | --- | --- | --- | --- | --- | --- | --- | --- | --- | --- | --- |
| 220×PH4CV | Number of Markers | 181 | 204 | 84 | 99 | 150 | 124 | 133 | 161 | 127 | 119 | 1382 |
|  | Total length (cM) | 158.7 | 231.5 | 151.8 | 240.7 | 132.0 | 118.3 | 137.3 | 210.5 | 122.0 | 187.0 | 1689.8 |
|  | Average Length (cM) | 0.9 | 1.1 | 1.8 | 2.5 | 0.9 | 1.0 | 1.0 | 1.3 | 1.0 | 1.6 | - |
| 220×Y1518 | Number of Markers | 214 | 192 | 188 | 75 | 175 | 116 | 143 | 166 | 129 | 102 | 1500 |
|  | Total length (cM) | 233.9 | 239.1 | 147.7 | 194.5 | 157.6 | 111.7 | 151.0 | 204.0 | 128.1 | 173.6 | 1741.2 |
|  | Average Length (cM) | 1.1 | 1.3 | 0.8 | 2.6 | 0.9 | 1.0 | 1.1 | 1.2 | 1.0 | 1.7 | - |
| P9-10×PH4CV | Number of Markers | 168 | 187 | 174 | 56 | 169 | 147 | 138 | 166 | 76 | 138 | 1419 |
|  | Total length (cM) | 216.2 | 189.6 | 200.7 | 158.0 | 160.4 | 135.0 | 129.4 | 290.1 | 222.7 | 177.9 | 1880.0 |
|  | Average Length (cM) | 1.3 | 1.0 | 1.2 | 2.9 | 1.0 | 0.9 | 0.9 | 1.8 | 3.0 | 1.3 | - |
| Consensus linkage map | Number of Makers | 381 | 338 | 300 | 151 | 326 | 243 | 257 | 283 | 189 | 225 | 2693 |
|  | Total length (cM) | 428.7 | 271.7 | 324.4 | 238.9 | 287.0 | 223.3 | 253.1 | 306.3 | 174.6 | 306.4 | 2814.4 |
|  | Average Length (cM) | 1.13 | 0.80 | 1.08 | 1.58 | 0.88 | 0.92 | 0.98 | 1.08 | 0.92 | 1.36 | - |

**Supplementary Table S5. QTL identified for emergence ratio (ER), germination index (GI), total length (TL), root length (RL) and shoot length (SL) from population 220 × PH4CV, 220 × Y1518 and P9-10 × PH4CV.**

| QTL Name ^a^ | Flanking Makers ^b^ | LOD | PV(%) ^c^ | Add ^d^ | Dom ^e^ | CI1 (cM) ^f^ | CI2 (cM) ^g^ | Reference ^h^ |
| --- | --- | --- | --- | --- | --- | --- | --- | --- |
| 220×PH4CV | |  |  |  |  |  |  |  |
| ER |  |  |  |  |  |  |  |  |
| *qp1ER1-1* | M1c180017078- M1c184012303 | 18.68 | 10.18 | 0.17 | 0.05 | 51.79- 53.16 | 183.96- 192.17 | *Yracheboud et al., 2002;  Hund et al., 2004.* |
| *qp1ER1-2* | M1c195770152- M1c196129249 | 8.39 | 4.05 | -0.09 | 0.05 | 63.85- 64.08 | 250.37- 254.70 | *Yracheboud et al., 2002;* |
| *qp1ER5-1* | M5c136200206- M5c137852051 | 13.25 | 6.90 | -0.13 | 0.02 | 56.00- 56.25 | 129.21- 129.46 | *Presterl et al., 2007;  Leipner et al., 2008; Guerra-Peraze et al., 2011.* |
| *qp1ER5-2* | M5c154808379- M5c155959609 | 26.82 | 16.38 | 0.20 | -0.01 | 60.80- 61.93 | 133.67- 137.22 |  |
| *qp1ER9-1* | M9c127216479- M9c129277973 | 8.94 | 4.47 | 0.10 | 0.18 | 66.62- 67.30 | 84.19- 90.74 | *Allam et al., 2016; Revilla et al., 2016.* |
| GI |  |  |  |  |  |  |  |  |
| *qp1GI2-1* | M2c196386747- M2c199180638 | 25.22 | 18.21 | 1.01 | 0.28 | 116.80- 117.70 | 163.75- 165.86 | *Huang et al., 2013.* |
| *qp1GI3-1* | M3c110461378- M3c178049748 | 6.00 | 0.62 | 0.53 | -0.10 | 56.01- 86.6 | 17.92- 54.73 | *Jompuk et al., 2005; Presterl et al., 2007;  Leipner et al., 2008;  Allam et al., 2016;  Revilla et al., 2016.* |
| *qp1GI5-1* | M5c154808379- M5c155959609 | 7.37 | 0.74 | 0.55 | -0.08 | 60.80- 61.93 | 133.67- 137.22 |  |
| *qp1GI9-1* | M9c117330059- M9c119863019 | 8.12 | 0.82 | 0.57 | 0.09 | 64.81- 65.03 | 86.00- 86.22 |  |
| TL |  |  |  |  |  |  |  |  |
| *qp1TL1-1* | M1c179183309- M1c184012303 | 22.69 | 33.01 | 1.49 | 0.81 | 45.76- 53.16 | 170.34- 192.17 | *Yracheboud et al., 2002; Hund et al., 2004.* |
| *qp1TL10-1* | M10c5874720- M10c6678670 | 4.22 | 5.02 | -0.53 | -0.25 | 14.23- 20.11 | 19.58- 72.29 | *Presterl et al., 2007; Revilla et al., 2016.* |
| RL |  |  |  |  |  |  |  |  |
| *qp1RL1-1* | M1c179183309- M1c184012303 | 17.63 | 26.10 | 0.97 | 0.59 | 50.42- 53.16 | 183.96- 192.17 | *Yracheboud et al., 2002; Hund et al., 2004.* |
| *qp1RL4-1* | M4c170791705- M4c171126206 | 4.42 | 5.37 | 0.37 | -0.28 | 80.43- 81.56 | 88.76- 89.89 |  |
| *qp1RL10-1* | M10c5874720- M10c6678670 | 5.33 | 7.05 | -0.44 | -0.26 | 14.23- 20.11 | 19.58- 72.29 | *Presterl et al., 2007; Revilla et al., 2016.* |
| SL |  |  |  |  |  |  |  |  |
| *qp1SL1-1* | M1c18202377- M1c21758216 | 6.35 | 4.53 | -0.19 | 0.07 | 2.83- 6.27 | 78.07- 92.54 |  |
| *qp1SL1-2* | M1c45338592- M1c49392023 | 6.54 | 4.64 | -0.02 | 0.27 | 24.71- 27.69 | 115.76- 125.27 |  |
| *qp1SL1-3* | M1c180017078- M1c184012303 | 30.09 | 28.18 | 0.52 | 0.09 | 51.79- 53.16 | 183.96- 192.17 | *Yracheboud et al., 2002;  Hund et al., 2004.* |
| *qp1SL9-1* | M9c131914243- M9c132551091 | 4.64 | 3.18 | 0.16 | 0.22 | 67.76- 68.21 | 91.35- 91.5 |  |
| *qp1SL9-2* | M9c150237694- M9c151277505 | 4.41 | 3.02 | 0.01 | -0.03 | 102.63- 105.14 | 141.99- 146.48 | *Jompuk et al., 2005;  Allam et al., 2016;* |
| 220×Y1518 | |  |  |  |  |  |  |  |
| ER |  |  |  |  |  |  |  |  |
| *qp2ER2-1* | M2c193833217- M2c196386747 | 4.57 | 4.81 | 0.08 | 0.02 | 111.92- 115.51 | 163.75- 165.86 |  |
| *qp2ER3-1* | M3c17284235- M3c17608001 | 8.75 | 9.10 | 0.11 | 0.02 | 52.36- 53.54 | 248.64- 249.46 | *Allam et al., 2016.* |
| *qp2ER9-1* | M9c94805463- M9c96001102 | 12.90 | 13.73 | 0.14 | 0.04 | 57.81- 58.04 | 74.74- 74.97 | *Leipner et al., 2008.* |
| GI |  |  |  |  |  |  |  |  |
| *qp2GI2-1* | M2c157899241- M2c164975838 | 4.08 | 6.16 | 0.45 | 0.20 | 81.76- 81.28 | 126.94- 128.43 |  |
| *qp2GI3-1* | M3c17284235- M3c17608001 | 7.35 | 11.22 | 0.60 | -0.01 | 52.36- 53.54 | 248.64- 249.46 | *Allam et al., 2016.* |
| *qp2GI9-1* | M9c94805463- M9c96001102 | 13.42 | 21.46 | 0.85 | 0.17 | 57.81- 58.04 | 74.74- 74.97 | *Leipner et al., 2008.* |
| TL |  |  |  |  |  |  |  |  |
| *qp2TL2-1* | M2c190934119- M2c191002402 | 5.08 | 9.83 | 0.36 | 0.30 | 102.66- 104.08 | 149.20- 149.91 | *Presterl et al., 2007.* |
| RL |  |  |  |  |  |  |  |  |
| *qp2RL8-1* | M8c146990749- M8c149037804 | 36.28 | 17.97 | -0.95 | -0.01 | 129.75- 130.69 | 205.81- 206.94 | *Jompuk et al., 2005.* |
| *qp2RL8-2* | M8c152144735- M8c153794835 | 24.49 | 10.44 | 0.72 | -0.04 | 134.99- 136.17 | 211.24- 212.42 |  |
| *qp2RL9-1* | M9c138243599- M9c142205835 | 5.89 | 2.03 | 0.31 | 0.08 | 76.00- 81.78 | 96.76- 102.13 | *Presterl et al., 2007;  Guerra-Peraze et al., 2011;  Allam et al., 2016.* |
| SL |  |  |  |  |  |  |  |  |
| *qp2SL2-1* | M2c190934119- M2c191818014 | 8.10 | 12.31 | 0.15 | 0.02 | 104.08- 105.98 | 149.91- 153.89 | *Presterl et al., 2007.* |
| *qp2SL3-1* | M3c15427752- M3c17003825 | 9.84 | 15.17 | 0.17 | 0.01 | 51.18- 52.13 | 249.61- 250.19 |  |
| *qp2SL8-1* | M8c170062609- M8c170861946 | 4.62 | 6.72 | 0.11 | 0.01 | 180.87- 188.23 | 278.50- 284.21 | *Presterl et al., 2007;  Leipner et al., 2008;  Rodríguez et al., 2014.* |
| P9-10×PH4CV | |  |  |  |  |  |  |  |
| ER |  |  |  |  |  |  |  |  |
| *qp3ER10-1* | M10c121724611- M10c123685470 | 8.90 | 15.53 | 1.42 | 0.54 | 41.67- 41.89 | 105.70- 105.92 |  |
| GI |  |  |  |  |  |  |  |  |
| *qp3GI10-1* | M10c121724611- M10c123685470 | 7.90 | 9.89 | 0.59 | 0.09 | 41.67- 41.89 | 105.70- 105.92 |  |
| TL |  |  |  |  |  |  |  |  |
| *qp3TL1-1* | M1c3192090- M1c3468972 | 5.81 | 6.13 | -0.42 | 0.08 | 0.68- 3.18 | 32.96- 36.82 | *Presterl et al., 2007.* |
| *qp3TL1-2* | M1c174799835- M1c183301837 | 29.01 | 39.44 | 1.09 | 0.83 | 98.72- 103.38 | 164.57- 223.23 | *Yracheboud et al., 2002;  Hund et al., 2004;  Allam et al., 2016.* |
| *qp3TL9-1* | M9c149041431- M9c150237694 | 4.75 | 5.04 | 0.39 | -0.04 | 87.07- 94.38 | 129.89- 146.48 | *Allam et al., 2016;  Hu et al., 2016.* |
| RL |  |  |  |  |  |  |  |  |
| *qp3RL1-1* | M1c174799835- M1c183301837 | 11.87 | 18.98 | 0.23 | 0.21 | 98.72- 103.38 | 164.57- 223.23 | *Yracheboud et al., 2002;  Hund et al., 2004;  Allam et al., 2016.* |
| *qp3RL2-1* | M2c109876726- M2c112639588 | 4.51 | 6.64 | 0.15 | 0.01 | 101.83- 102.06 | 116.46- 116.68 |  |
| *qp3RL8-1* | M8c119494453- M8c120875063 | 5.41 | 7.89 | 0.15 | 0.09 | 110.83- 111.06 | 193.08- 192.74 |  |
| SL |  |  |  |  |  |  |  |  |
| *qp3SL1-1* | M1c3192090- M1c3468972 | 3.82 | 4.10 | -0.25 | 0.06 | 0.68- 3.18 | 32.96- 36.82 | *Presterl et al., 2007.* |
| *qp3SL1-2* | M1c174799835- M1c183301837 | 27.20 | 38.72 | 0.84 | 0.60 | 98.72- 103.38 | 164.57- 223.23 | *Yracheboud et al., 2002;  Hund et al., 2004;  Allam et al., 2016.* |
| *qp3SL9-1* | M9c149041431- M9c150237694 | 4.30 | 4.71 | 0.29 | -0.04 | 87.07- 94.38 | 129.89- 146.48 | *Allam et al., 2016;  Hu et al., 2016.* |

^a,^ QTL named following qtl (q) + population (p; p1, p2 and p3 represented population 220 × PH4CV, 220 × Y1518 and P9-10 × PH4CV, respectively) + trait + chromosome + '-' + order in this chromosome.

^b^, Marker named following marker (M) + chromosome number + 'c' + its physical position in B73 RefGen_v2;

^c^, PV represented phenotypic variation explained by individual QTL;

^d^, positive add value mean favor allele contributed by tolerant lines 220 or P9-10;

^e^, positive dom value means dominant allele contributed by tolerant lines 220 or P9-10;

^f^, CI1 indicated confidence interval in each linkage map;

^g^, CI2 indicated confidence interval in the consensus linkage map;

^h^, reference indicted that the QTL was overlapped by QTL identified in this study.

**References**

Allam, M., Revilla, P., Djemel, A., Tracy, W. F., Ordas, B. (2016). Identification of QTLs involved in cold tolerance in sweet × field corn. *Euphytica* 208, 353-365. doi:10.1007/s10681-015-1609-7

Fracheboud, Y., Ribaut, J. M., Vargas, M., Messmer, R., Stamp, P. (2002). Identification of quantitative trait loci for cold-tolerance of photosynthesis in maize (*Zea mays* L.). *J. Exp. Bot.* 53, 1967-1977. doi: 10.1093/jxb/erf040

Guerra-Peraza, O., Leipner, J., Reimer, R., Nguyen, H. T., Stamp, P., Fracheboud, Y. (2012). Temperature at night affects the genetic control of acclimation to cold in maize seedlings. *Maydica* 56 (4), 367-377.

Hu, S., Lübberstedt, T., Zhao, G., Lee, M. (2016). QTL mapping of low-temperature germination ability in the maize IBM syn4 RIL population. *PloS ONE* 11, e152795. doi: 10.1371/journal.pone.0152795

Huang, J., Zhang, J., Li, W., Hu, W., Duan, L., Feng, Y., Qiu, F. et al. (2013). Genome‐wide association analysis of ten chilling tolerance indices at the germination and seedling stages in maize. *J. Integr. Plant Biol.* 55, 735-744. doi: 10.1111/jipb.12051

Hund, A., Fracheboud, Y., Soldati, A., Frascaroli, E., Salvi, S., Stamp, P. (2004). QTL controlling root and shoot traits of maize seedlings under cold stress. *Theor. Appl. Genet.* 109, 618-629. doi: [10.1007/s00122-004-1665-1](https://doi.org/10.1007/s00122-004-1665-1)

Jompuk, C, Fracheboud, Y., Stamp, P, Leipner, J. (2005). Mapping of quantitative trait loci associated with chilling tolerance in maize (*Zea mays* L.) seedlings grown under field conditions. *J. Exp. Bot.* 56, 1153-1163. doi: 10.1093/jxb/eri108

Leipner, J., Jompuk, C., Camp, K., Stamp, P., Fracheboud, Y. (2008). QTL studies reveal little relevance of chilling-related seedling traits for yield in maize. *Theor. Appl. Genet.* 116, 555-562. doi: 10.1007/s00122-007-0690-2

Presterl, T, Ouzunova, M., Schmidt, W., Moller, E.M., Rober, F.K., Knaak, C., Ernst, K. et al. (2007). Quantitative trait loci for early plant vigour of maize grown in chilly environments. *Theor. Appl. Genet.* 114, 1059-1070. doi: 10.1007/s00122-006-0499-4

Revilla, P., Rodríguez, V. M., Ordas, A., Rincent, R., Charcosset, A., Giauffret, C., Melchinger, A. E. et al. (2016) Association mapping for cold tolerance in two large maize inbred panels. *BMC Plant Biol.* 16, 127. doi: 10.1186/s12870-016-0816-2

Rodríguez, V.M., Butrón, A., Rady, M.O.A., Soengas, P., Revilla, P. (2014). Identification of quantitative trait loci involved in the response to cold stress in maize (*Zea mays* L.). *Mol. Breeding* 33, 363-371. doi: 10.1007/s11032-013-9955-4
